# The Temporal Response Surface: A Novel Method for the Assessment of Delayed and Time-Cumulative Aquatic Ecosystem Risk

**DOI:** 10.1021/acs.est.4c14331

**Published:** 2025-05-19

**Authors:** Cath A. Neelamraju, Reinier M. Mann, Michael St J. Warne, Francisco Sanchez-Bayo, Ryan D.R. Turner

**Affiliations:** 1 The Reef Catchments Science Partnership, School of the Environment, The University of Queensland, St Lucia, Brisbane, Queensland 4067, Australia; 2 Queensland Department of Environment, Tourism, Science and Innovation, Ecosciences Precinct, 41 Boggo Rd, Dutton Park, Brisbane, Queensland 4102, Australia; 3 School of Life and Environmental Sciences, The University of Sydney, 1 Central Avenue, South Eveleigh, New South Wales 2015, Australia; 4 Sustainable Minerals Institute, The University of Queensland, St Lucia, Brisbane, Queensland 4067, Australia

**Keywords:** chronic exposure, ecosystem protection guidelines, Great Barrier Reef, imidacloprid, species sensitivity distribution, time-cumulative toxicity

## Abstract

In Australia, pesticide risk assessments in aquatic environments typically compare measured water concentrations to relevant ecosystem protection guidelines based on species sensitivity distributions (SSDs). These guidelines estimate concentrations that are protective against long-term (chronic) exposure but do not consider extended exposure to chemicals with cumulative toxicity, such as neonicotinoid insecticides. The Australian and New Zealand Guidelines caution against the application of default acute to chronic ratios (ACRs) for such chemicals but lack suitable alternatives. Addressing this gap, we introduce the Temporal Response Surface (TRS) method that incorporates exposure duration into SSDs via taxa-specific regression relationships. The TRS considers both toxicity and exposure duration, setting meaningful guidelines for aquatic ecosystem protection and facilitating probabilistic risk assessments. Applied to imidacloprid, concentrations that are protective of 99, 95, 90, and 80% of the ecosystem (PC99, PC95, PC90, and PC80, respectively) drop quickly within the first 30 days of continuous exposure, then progressively decline over longer exposure periods. This suggests that previous risk assessments may have underestimated the toxic effects of prolonged or repeated imidacloprid exposure. The TRS method offers a holistic solution, addressing policy and risk assessment gaps for chemicals with delayed or cumulative toxicity, further enhancing aquatic ecosystem protection in Australia and internationally.

## Graphical abstract

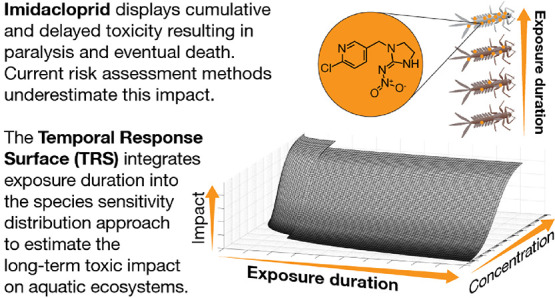

## Introduction

Globally, there is concern about the increasing detection of pesticides in waterbodies, as research has shown that these chemicals threaten aquatic ecosystem health and function.^10,23,29,38,56^ Neonicotinoid insecticides like imidacloprid have been implicated in aquatic macroinvertebrate decline, reduced biodiversity, disrupted food webs, and impaired ecosystem services.^42,60,66^ Neonicotinoids disrupt the normal transmission of nervous system impulses by irreversibly binding to, and continuously activating the nicotinic acetylcholine receptors (nAChR) embedded in neurons of insects and other sensitive organisms.^3,33^ As exposure continues, the increasing number of bound nAChRs results in a generalized inactivation of nerve function, hyperexcitation, convulsions, paralysis, and eventual death of the organism.^53^ These toxic effects can occur after a single extended exposure or repeated pulses of neonicotinoids. Over time, the aqueous concentration required to elicit a toxic effect reduces; a process that has been described as time-cumulative toxicity by Sánchez-Bayo and Tennekes.^54^ Therefore the frequency and magnitude of imidacloprid exposure require careful consideration in risk assessment, as the effects are cumulative and recovery is slow (S10,^18^). Research indicates that aquatic ecosystem impacts in the form of altered community structure and function are possible at imidacloprid concentrations that commonly occur in some parts of the Great Barrier Reef catchment area.^2,13,28,41,71^ For example, a mesocosm study by Merga and Van den Brink^36^ found that aquatic ecosystem community structure was significantly affected at a 112-day time weighted average imidacloprid concentration of ≥ 0.02 μg/L. Warne M St J, Turner et al.^69^ assessed imidacloprid concentrations in frequently sampled waterways discharging to the Great Barrier Reef from 2009 to 2017. The average annual concentration of imidacloprid exceeded 0.02 μg/L at nine of the 15 sites studied. To assess the impact of such exposures on aquatic ecosystems, it is necessary to estimate the biological effects of chronic or repeated exposure at sublethal concentrations. However, chronic imidacloprid toxicity data are often sparse due to the high cost and logistical constraints of bioassay tests.

The Australian and New Zealand Guidelines for Fresh and Marine Water Quality^5^ (henceforth termed the ANZ Guidelines) allow for supplementation of chronic species sensitivity distributions (SSDs) with acute toxicity data that have been converted to an estimate of chronic toxicity.^67^ Chronic toxicity herein refers to an effect that occurs following exposure to a chemical for a period of time that represents a substantial portion of an organism’s life span (greater than 10%) (see Table 1 of^67^). Conversion is achieved through the application of an acute to chronic ratio (ACR); i.e., the ratio of acute toxicity (LC50 or EC50) to chronic toxicity (the no observed effect concentration (NOEC), or EC10) for a particular chemical. One important consideration in the application of ACRs is whether they can estimate chronic effects with an appropriate level of confidence and at an appropriate time frame for chemicals that amplify in toxic effect over time.^24^ Of particular interest are toxicants that display delayed or cumulative toxicity, such as neonicotinoid insecticides, organophosphorus insecticides, and mercury.^21,26,43,54^ The ANZ Guidelines caution that applying ACRs to such chemicals could result in guidelines that provide insufficient ecosystem protection.^67^ It is, therefore, essential to develop techniques to better estimate chronic impacts for the establishment of meaningful ecosystem protection guidelines. One well-published approach involves the prediction of chronic lethality using regression analysis of time series acute bioassay data.^15,35,52,55^ For example, Kumar, Correll et al.^25^ developed regression relationships using acute experimental data that produced reliable predictions of chronic lethality (i.e., within the confidence intervals of concurrent chronic bioassay tests) for six pesticides to the freshwater shrimp *Paratya australiensis*. Three of these pesticides were insecticides known to exhibit cumulative toxicity: chlorpyrifos and dimethoate (both organophosphorus insecticides), and cypermethrin (a synthetic pyrethroid).^32,46^ They further propose that predictions of chronic toxicity from time series regression can be used in ecological risk assessments with reasonable confidence where no measured estimates of chronic toxicity are available. This approach should extend to neonicotinoids, which also exhibit time-cumulative toxicity. Although effective for individual species, there is a need to apply these relationships at the ecosystem level for the development of meaningful protection guidelines as mandated by the Australian Water Quality Management Framework.^7^

The main objectives of the current study were 3-fold: (i) to develop log–log regression relationships capable of estimating the chronic toxicity of imidacloprid to nontarget aquatic organisms, (ii) to develop a Temporal Response Surface (TRS) using these regression relationships for estimation of ecosystem level effects, and (iii) to assess the suitability of this approach compared with the ACR method. Regression relationships were developed for representatives of key taxa (Insecta, Malacostraca, and Branchiopoda) that were then used to derive temporal adjustment factors (TAFs) that were applied across all species in an SSD. This allowed for a third axis representing exposure duration to be incorporated into the traditional SSD approach,^44,62^ effectively creating a three-dimensional TRS. While initially applied to imidacloprid, the methodology is valuable for assessing chemical sensitivity under a range of additional variables, such as pH or temperature. The TRS addresses a gap in global risk assessment frameworks and demonstrates the broader utility of the method.

## Methods

Figure 1 illustrates the method steps and validation procedures utilized to build the TRS and will be referred to throughout the method section.

**Figure 1:**
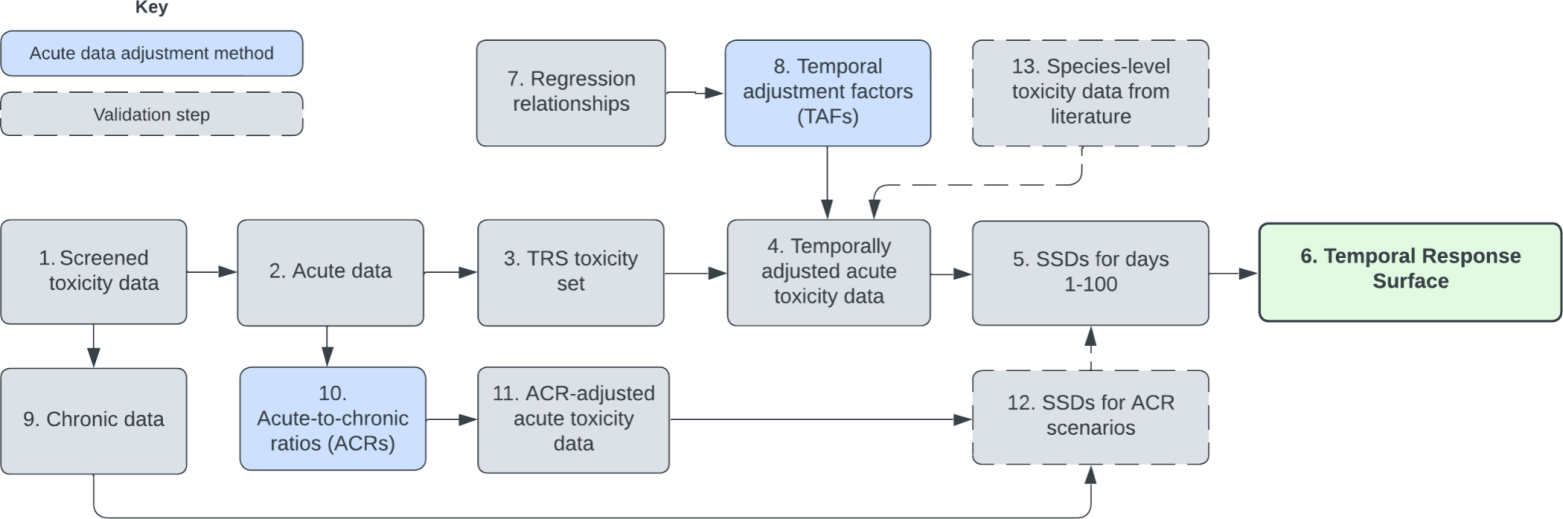
A conceptual diagram of the Temporal Response Surface method, showing the data types and methods used to validate the approach.

### Toxicity Data Collation and Quality Screening

Toxicity data were curated to support four separate processes, as outlined below:

1. Derivation of a chronic SSD in line with the ANZ Guideline method^67^ (Figure 1, step 12),
2. Development of log–log regression relationships between effect concentration and exposure duration (Figure 1, step 7),
3. Development of the toxicity data set underlying the TRS (Figure 1, step 3), and
4. Calculation of ACRs for comparison of the TRS protective concentrations with those derived using other available methods (Figure 1, steps 10–12).

Toxicity data were obtained through a comprehensive review of literature and the databases of USEPA ECOTOX,^63^ Office of the Pesticide Program,^64^ Australasian Ecotoxicology Database^70^ and the ANZECC and ARMCANZ toxicant database.^58^ Only toxicity data generated by exposing test organisms to a relatively pure form of imidacloprid (>80% active ingredient) were used. Toxicity data based on formulation products were not used. Data quality was assessed using the nationally endorsed ANZ Guideline method, which examines how each toxicity value was generated and awards a quality score on the basis of answers to a series of questions (see Table 3 and Appendix 1 of^67^). According to the ANZG method (^67^), only ‘high’ (≥80%) and ‘acceptable’ (≥50% to <80%) quality toxicity data may be used to derive water quality guidelines. Acute LC50 or EC50 values were converted to estimates of acute LC10 or EC10 values by dividing by 5, according to guidance in the nationally endorsed method.^67^ Where multiple toxicity estimates were available for a species these were geomeaned to calculate a single value prior to derivation of the SSDs.^67^ A summary of the final chronic and acute toxicity data that passed the quality assurance and screening processes are provided in the Supporting Information (S1).

### Calculation of Species Sensitivity Distributions and Protective Concentrations

Species sensitivity distributions and protective concentrations were derived according to the ANZ Guideline method^9,67^ using the ssdtools R package.^59^ Five unimodal distributions were fitted to the imidacloprid toxicity data: gamma, log-Gumbel (also known as inverse Weibull), log–logistic, log-normal, and Weibull. The SSD models were assessed visually using distribution plots and statistically using goodness-of-fit statistics. Upper and lower 95% confidence intervals were generated using parametric bootstrapping (1,000 iterations) and overlaid on the SSD to aid in a visual assessment of goodness-of-fit. Parametric bootstrapping (10,000 iterations) was used to generate the protective concentration (PC) values that would protect 99%, 95%, 90% and 80% of species (PC99, PC95, PC90 and PC80, respectively). This method was applied to derive SSDs and PC values for each time step (days 1–100) of the TRS. Chronic toxicity data (as defined by the classification system in Table 1 of^67^) were not considered in the TRS because the aim of the regression relationships is to convert toxicity estimates in daily time steps from 1 to 100 days of continuous exposure. Most chronic toxicity data were reproduction or development-based endpoints so it was deemed inappropriate to convert these to estimates of acute toxicity, as reproduction and development generally do not occur at this time frame. Therefore, seven chronic data points were not used in the TRS but were included in the toxicity data for the ACR-adjusted scenarios below. The remaining TRS toxicity data set contained 32 acute data points, all of them adjusted by regression relationships. These data will hereafter be referred to as the ‘TRS toxicity set’.

An additional four SSDs were derived to assess the suitability of regression relationships for adjustment of acute toxicity data, through application of various ACRs to convert acute toxicity data to estimates of chronic toxicity (Figure 1, steps 10–12):

1. Literature-derived, species-specific ACRs applied by taxonomic group (see S6),
2. A default ACR of 10 as recommended by the ANZ Guidelines,^67^
3. A default ACR of 50 as recommended by Calabrese and Baldwin,^12^ and
4. A default ACR of 100 as recommended by May, Drost et al..^34^

A total of 43 fresh and marine species were used to derive SSDs for each of the above ACR scenarios (where ‘scenario’ refers to application of a specific ACR type, as listed above), comprising a combination of chronic (18.6%) and acute (81.4%) toxicity data. The acute data were converted using the above ACRs (e.g., dividing the concentration value by 50 for the default ACR of 50) then combined with existing chronic toxicity data to derive ACR-adjusted SSDs and protective concentrations for each scenario.

### Development of Regression Relationships

Robust statistical relationships between effect concentration and exposure duration require time-series bioassay data on toxic effects across multiple time points for individual species (e.g., 4, 7, 11, and 14 days). According to OECD guidelines, a minimum of five data points is acceptable for the determination of a statistical relationship between a measured response (e.g., LC50) and the analyte concentration in a sample.^39^ Three separate regression relationships were developed (*Deleatidium sp.*, *Hyalella azteca*, and *Daphnia magna*), as per methods outlined in Sánchez-Bayo^52^ (Figure 1, step 7). All toxicity data passed the quality assessment process outlined in Warne M St J, Batley et al.,^67^ except for *Deleatidium* sp., which had poor control survivorship in one of the papers.^31^ These data were deemed suitable for the Insecta regression relationship (see discussion in S4: *Deleatidium* sp.); however, we recommend this process be repeated once better-quality data become available. All three data sets displayed less variance in the predictor (exposure duration) than the response (effect concentration); therefore, a Gaussian generalized linear model (GLM) was applied to the natural log of the two variables using the following formula:

lnEC=β×lnED+ε 1

where *ln EC* refers to the natural log of the effect concentration in μg/L, β is the regression slope estimate, *ln ED* is the natural log of the exposure duration in whole days, and ε is the residual error. An iterative approach was taken in which each model was assessed for goodness of fit, satisfaction of model assumptions, and influential points (if any) were removed. The final model for each species was chosen based on the fit of the model, diagnostic figures that examine the satisfaction of underlying GLM assumptions, and the D-squared value (the equivalent of an R^2^ for GLM models) (see S4). The predictive performance of the regression models was estimated using leave-one-out-cross-validation (LOOCV) and estimation of the Mean Square Error (MSE).^74^ An average MSE close to zero indicates high accuracy and minimal error between the observed and predicted values.^22^ The regression relationships were initially used to estimate the effect concentration in μg/L from days 1 to 365 for each of the modeled organisms using the predict function in R,^50^ which generates a point estimate and 95% confidence intervals using parametric bootstrapping (1000 iterations). The extrapolation of statistical relationships derived from relatively short durations (9.39 days for *D. magna*, 28 days each for *Deleatidium* sp. and *H. azteca*) to much longer durations (365 days) will carry inherent uncertainty. Inflection points were calculated to determine what portion of the 365-day exposure window would be reasonable to use, resulting in a 100-day exposure window for the development of the TRS. The inflection point method is detailed in the Supporting Information (S5).

### Calculation of Adjustment Factors Using Log–Log Regression

Much like ACRs, the regression predictions represent estimates of toxicity at different exposure durations and can be used to understand the relationship between acute and chronic toxicity in much the same way. However, where ACRs represent a ratio between specific time points (e.g., as a ratio between 4 days and 28 days of exposure), the modeled regression data are produced at continuous daily time steps. The ratios were termed Temporal Adjustment Factors (TAFs) to distinguish them from the ACR approach (Figure 1, step 8), and were calculated using the following formula:

$$TAF=\frac{{ECmodelled}_{x}}{ECmeasured}$$ 2

where *ECmodelled*_*x*_ is the predicted effect concentration for each reference species on Day *x*, and *ECmeasured* is the measured (observed) effect concentration of the toxicity data.

### Development of the Temporal Response Surface

Research has shown that closely related species have similar toxic responses.^16,17,20,51^ As a result, TAFs were applied to the acute toxicity data for each species in the TRS toxicity set according to their Classes using the following formula:

$$Adjusted\;EC_{x=}\frac{EC_{y}}{TAF_{x}}$$ 3

where *Adjusted EC*_*x*_ is the estimated chronic NOEC or EC10 effect concentration on Day *x* (e.g., day 50) for a given species, *EC*_*y*_ is the unadjusted effect concentration in the TRS toxicity set, and *TAF*_*x*_ is the temporal adjustment factor of the reference species on Day *x*. The models for *Deleatidium sp.*, *Hyalella azteca*, and *Daphnia magna* represent the Classes Insecta, Malacostraca, and Branchiopoda, respectively. The toxicity data were adjusted once for each day of exposure from 1 to 100 days to produce a single temporally adjusted toxicity estimate for each species and day. An SSD was then derived for each temporally adjusted data set using the same method as that used to generate the ACR-adjusted SSDs (Figure 1, step 5). The 100 temporally adjusted SSDs were plotted in order of exposure duration to form the TRS (Figure 1, step 6).

### Comparative Evaluation of Toxicity Estimates

The TRS method uses regression relationships to adjust acute toxicity data, rather than the application of ACRs as is the standard practice when supplementing chronic data for the derivation of water quality guidelines in Australia.^67^ As this is a new method, several approaches were used evaluate the reliability of the TRS approach (Figure 1, steps 12 and 13):

1. The regression-adjusted toxicity values for individual species were compared with those found in the literature where available,
2. The TRS protective concentrations were compared with those of SSDs derived using ACRs from the literature (S6),
3. The TRS protective concentrations were compared with those derived using default ACRs of 10 (ANZ Guideline method), 50, and 100 (S6), and
4. A literature review was conducted to compare TRS toxicity estimates with ecological impacts observed in meso- and microcosm studies (S10).

For approach number 2, three studies from the open literature had suitable data for the derivation of species-specific imidacloprid ACRs in two crustaceans and seven insect species (Table 4 of S6). These ACRs could not be used to derive water quality guidelines due to either low chemical purity, use of formulation product, or high mortality in the control treatments. However, the ACRs were deemed suitable for sanity-checking of the TRS protective concentrations as they should approximate the actual increase in toxicity over time.

## Results and Discussion

### Calculation of SSDs and Protective Concentrations

There were toxicity data for 43 arthropod species (five classes within one phyla) and 14 non-arthropod species (seven classes across six phyla) that passed the screening and quality assessment process. The toxicity data were split, and only arthropod toxicity data were used for the current study as per the recommendations of Warne M St J, Batley et al.^67^ and Oginah, Posthuma et al.^40^ (SI, S2). The use of less than four taxonomic groups (phylum) to derive SSDs is permissible when the data are bimodal and need to be split to only the more sensitive group of organisms – in this case, arthropods.^67^ The represented arthropod Classes were Maxillopoda (copepods and barnacles), Branchiopoda (water fleas and fairy shrimp), Insecta (insects such as beetles, mayflies, and mosquitos), Malacostraca (crabs, prawns, and shrimp), and Ostracoda (seed shrimp). Of the freshwater species, there were chronic NOEC or EC10 type toxicity data for six species and acute toxicity data for another 29 species. In marine waters there were chronic NOEC or EC10 type toxicity data for two species and acute toxicity data for six species. These 43 data points were pooled to derive SSDs and protective concentrations in line with the ANZ Guidelines method (using a default ACR of 10), as well as the derivation of other ACR-adjusted SSDs used for validation of the TRS (see SI, S2 and S6). The six freshwater and two marine chronic NOEC or EC10 type toxicity data were not used for the TRS, as it was deemed inappropriate to adjust the reproduction and development-based end points to estimates of acute toxicity. Toxicity data for three Ostracoda species (*Cypretta seurati*, *Cypridopsis vidua*, *Ilyocypris dentifera*) were excluded from the TRS toxicity data set due to insufficient data to build a reliable regression model for this Class. Development of the ANZ Guideline SSD both with and without these data points had very little impact on the SSD fit, reliability, and protective concentrations (S1 and S3).

### Regression Relationships

Toxicity data sets with sufficient temporally spaced endpoint statistics (i.e., LC50) were identified for three species: the mayfly *Deleatidium sp.*,^30,31^ the freshwater amphipod *Hyalella azteca*,^8,45^ and the water flea *Daphnia magna*.^19^ These species are members of classes Insecta, Malacostraca, and Branchiopoda, respectively. The selected data were mortality endpoints of at least 50% effect. The regression models were developed for each species using an iterative approach to assess goodness-of-fit, ensure model integrity, and address influential observations. All models validated well with high D^2^ values and low MSEs, indicating good predictive performance (S1 and Figure 2). The toxicity data used to develop the three regression models can be found in S12, and a comparison of modeled outcomes with those toxicokinetic/toxicodynamic (TK/TD) studies is provided in S10.

**Figure 2:**
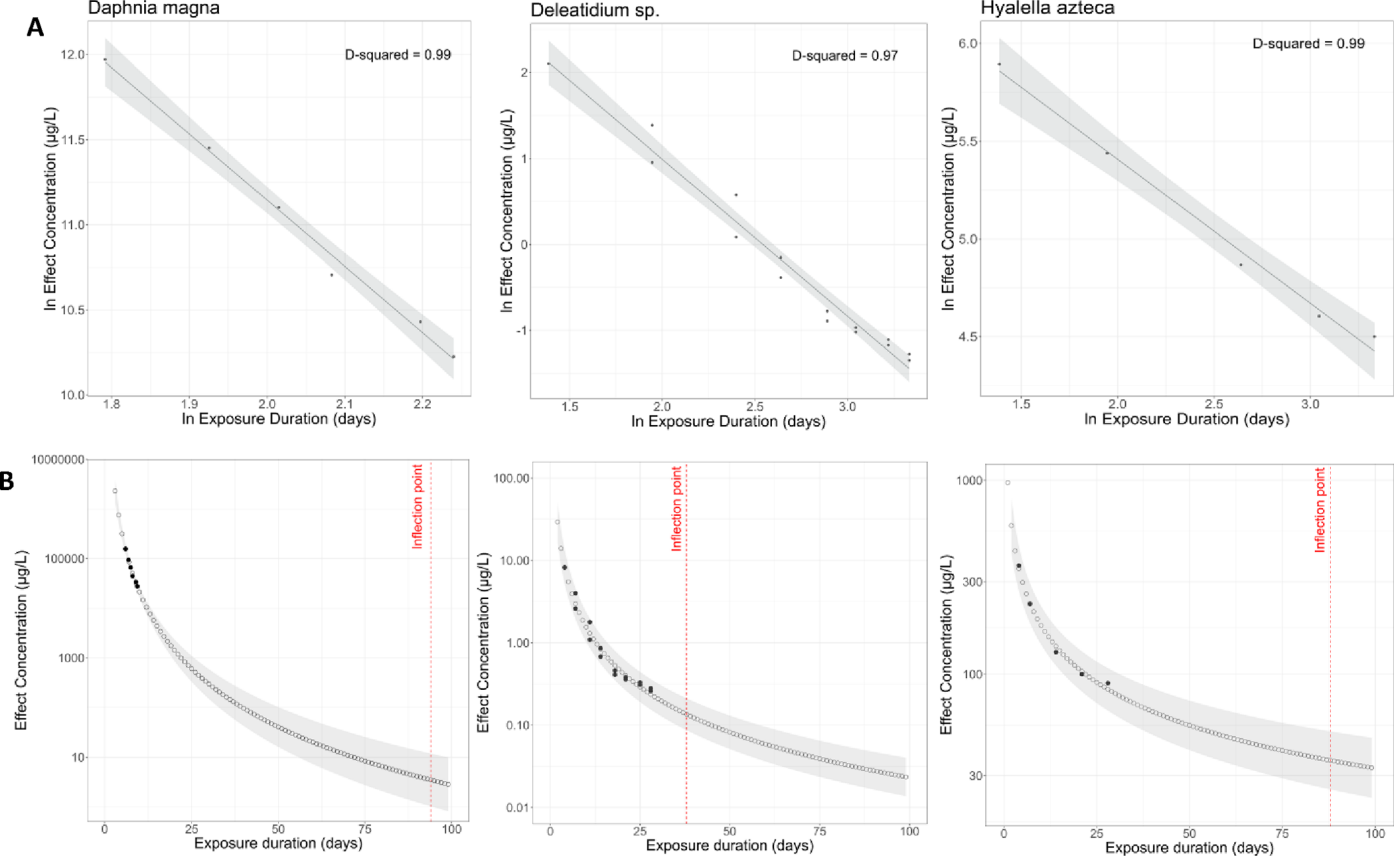
(A) Gaussian Generalized Linear Models (GLMs) with identity link were fitted to the imidacloprid toxicity data forDaphnia magna, *Deleatidium sp.*, andHyalella azteca, resulting in D-squared values of 0.99, 0.97, and 0.99, respectively. (B) The models were then used to predict the imidacloprid effect concentration for each species from 1 to 100 days of exposure (black dots are observed values, hollow circles are predicted values). This time span captured the majority of change in imidacloprid toxicity for the three species, visible as red dashed lines (refer to S5 for discussion on inflection points).

The model variables, coefficients, and inflection points for the *Daphnia magna, Deleatidium* sp., and *Hyalella azteca* models are shown in Table 1.

**Table 1:** Regression Model Variables, Their Coefficients, Standard Error, and Probability Values for Each of the Three Reference Species^a^

species	variable	coefficient	standard error	*t* value	probability	inflection point (days of exposure)
Daphnia magna	*y*-intercept	18.92	0.356	53.22	7.46 × 10^–7^	94
	ln exposure duration	–3.892	0.174	–22.41	2.35 × 10^–5^	
*Deleatidium* sp.	*y*-intercept	4.646	0.232	20.01	3.78 × 10^–11^	38
	in exposure duration	–1.827	0.085	–21.60	1.43 × 10^–11^	
Hyalella azteca	y-intercept	6.881	0.105	65.88	7.71 × 10^–6^	88
	ln exposure duration	–0.737	0.041	–18.14	3.66 × 10^–4^	

a Also included are the inflection points where the rate of change between successive modelled toxicity estimates reduces to almost nothing.

### Temporal Response Surface

The development of the imidacloprid TRS required the application of TAFs, calculated using modeled regression data for the three reference species, to the TRS toxicity set based on the organism Class to which each species belonged. An assumption was made that the regression slope, i.e., the rate at which an organism becomes more sensitive, is consistent between species within the same Class. This is reasonable because phylogenetic information has been shown to accurately predict the toxicity of chemicals, as closely related species often respond similarly.^16,17,20,51^ The time span of 100 days captures the majority of change in the three regression models (Table 1 and S5), resulting in a TRS that also spans 100 days. The modeled organisms (*Hyalella azteca, Deleatidium* sp., and *Daphnia magna*) became increasingly sensitive to imidacloprid at different rates (Figure 2), resulting in a change in the TRS distribution type that was chosen as the best fit over time (log Gumbel was chosen for days 1–17, then log-normal from days 18–100). The protective concentrations derived from the regression-adjusted toxicity data became progressively lower over time (Table 2).

**Table 2:** Protective Concentrations for the Temporally Adjusted Species Sensitivity Distributions for Selected Exposure Durations of the Temporal Response Surface^a^

	protective concentrations (μg/L)									
protection level	day 10	day 20	day 30	day 40	day 50	day 60	day 70	day 80	day 90	day 100
PC99	0.038	0.0023	0.00088	0.00043	0.00025	0.00015	0.00010	0.000072	0.000053	0.000040
PC95	0.089	0.013	0.0056	0.0030	0.0018	0.0012	0.00084	0.00062	0.00047	0.00037
PC90	0.15	0.033	0.015	0.0083	0.0052	0.0036	0.0026	0.0020	0.0015	0.0012
PC80	0.31	0.10	0.049	0.028	0.019	0.014	0.010	0.0078	0.0062	0.0051

a All values have been rounded to two significant figures. The protective concentrations for all exposure durations (days 1–100) can be found in the Supplementary Information (S11).

The temporally adjusted SSDs were plotted in order from day 1 – 100, allowing for the incorporation of exposure duration as a third dimension into the traditional SSD approach (Figure 3).

**Figure 3:**
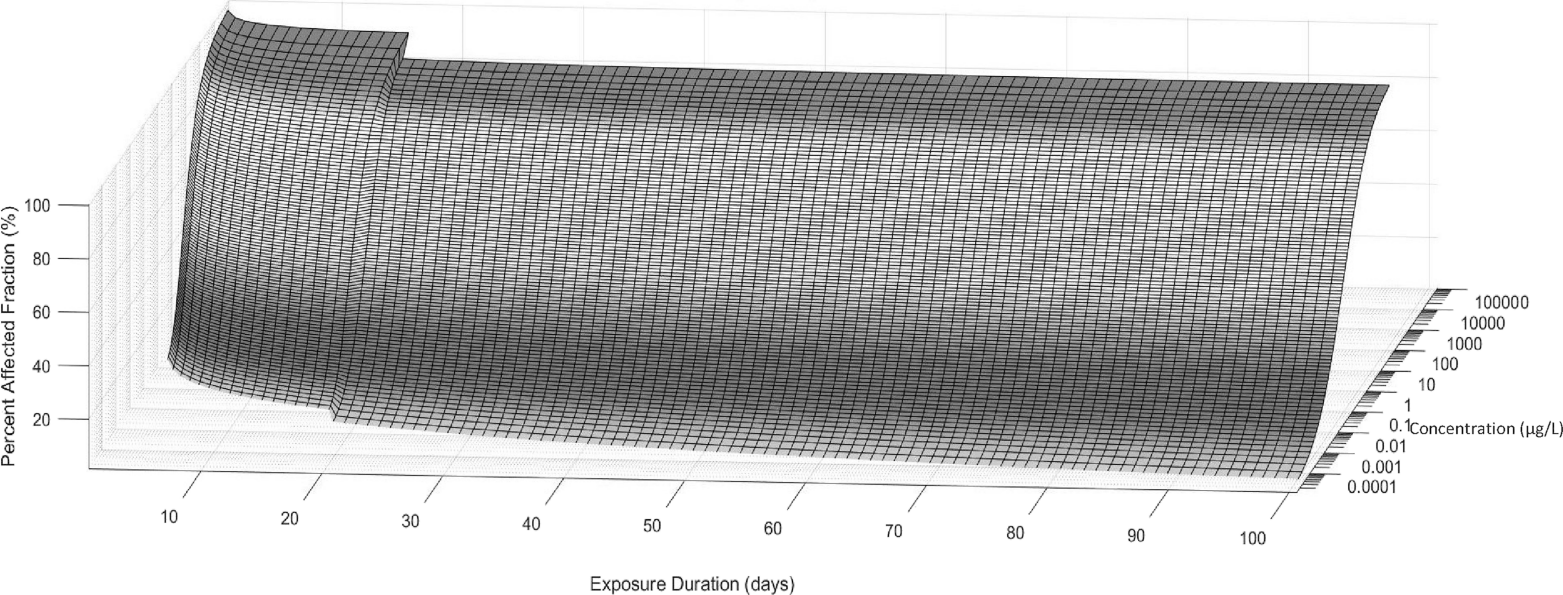
Temporal response surface for imidacloprid. All SSDs in this plot were produced using ssdtools,^59^ the vertical "wobble" in the surface is due to a change from log Gumbel to log-normal distribution type at day 18.

The TRS illustrates the progression of imidacloprid toxicity at the ecosystem level over time. The concentrations that are protective of 99%, 95%, 90% and 80% of the ecosystem (PC99, PC95, PC90, and PC80, respectively) drop quickly within the first 30 days of continuous exposure, then continue to decrease for the remainder of the 100-day period (Table 2 and Figure 3). This emphasizes the importance of considering extended exposure durations for the ecological risk assessment of imidacloprid. Analytical laboratories have a detection limit below which chemical concentrations cannot be accurately measured. The TRS PC99 drops below the 0.02 μg/L imidacloprid limit of reporting utilized by the Great Barrier Reef Catchment Loads Monitoring Program^72^ following 14 days of continuous exposure and the PC95 drops below this level at 18 days (S8). Gazetted freshwater systems in Queensland, including rivers and creeks, generally have a protection goal of 95% of species protected.^5,67^ Therefore, it may not be possible to assess whether aquatic ecosystems in the Great Barrier Reef catchment area are sufficiently protected from the effects of imidacloprid after just 18 days of continuous exposure.

A secondary advantage of the TRS method is that it can be used within a probabilistic risk assessment framework when both concentration and exposure duration are known. For example, Sumon, Ritika et al.^57^ calculated a 28-day imidacloprid zooplankton community NOEC of 0.03 μg/L in a subtropical microcosm study in Bangladesh. Using the TRS log-normal distribution equation at day 28, a concentration of 0.03 μg/L equates to approximately 12% of the ecosystem potentially affected. The higher estimate (12% PAF compared to a NOEC) is likely because the TRS includes toxicity data for more sensitive taxa (e.g., mayflies), as well as crustacea. Therefore, the observations of Sumon, Ritika et al.^57^ appear consistent with the TRS predictions. The TRS PC95 drops to 0.008 μg/L following 28 days of continuous exposure (S11). Since this value is below the NOEC observed by Sumon, Ritika et al.^57^ we can deem the TRS PC95 to be protective of both the zooplankton community and more sensitive organisms for the kinds of exposure observed. Warne M St J, Turner et al.^69^ assessed imidacloprid concentrations in waterways discharging to the Great Barrier Reef from 2009 to 2017. Water samples were collected monthly during the dry season (May to October) and intensively during high-flow events in the wet season (November to April). Concentrations peaked in the wet season. The highest average annual concentration was 0.2 μg/L for Sandy Creek at Homebush in the Mackay Whitsunday region (the 95th percentile and maximum were 0.76 μg/L and 1.2 μg/L, respectively). Using a traditional SSD approach, approximately 5% of the ecosystem would potentially be affected as a result of exposure to 0.2 μg/L (Table 2 of S2). Being a gazetted freshwater system, this concentration would not be considered problematic. However, given the temporally dense sampling and high detection frequency at this site (imidacloprid was detected in 89% of samples), it is reasonable to assume near-constant exposure of at least 100 days. Published monitoring data^71^ shows elevated imidacloprid concentrations at this site for months at a time. An imidacloprid concentration of 0.2 μg/L for a period of 100 days equates to approximately 61% of the ecosystem potentially affected using the TRS method. Species in this range (i.e., the species that fall below the 61% point on the *y*-axis of the 100-day TRS SSD, S9) represent a range of functional groups that collectively regulate algal growth, process organic matter, cycle nutrients, aerate sediments, and serve as prey to higher trophic levels.^49^ More sensitive species at the lower end of the SSD would experience higher impacts. Based on this finding and the outcomes of other studies (e.g.,^36,37,47,61^ ), it is likely that exposure to a concentration of 0.2 μg/L for 100 days would have a significant impact on the structure and function of aquatic invertebrate communities in these waterways. This estimate does not account for the impact of higher imidacloprid concentrations during runoff events.

### Comparative Evaluation of Toxicity Estimates

The regression-adjusted toxicity estimates for individual species were compared with toxicity data found in the literature for the same species, endpoint and exposure duration where available. Data for four species were found that could be used for validation purposes (Table 3). The adjusted toxicity values for *Asellus aquaticus*, *Gammarus pulex*, and *Caenis horaria* fell within the confidence intervals of existing data. The regression-adjusted LC10 value for *Culex pipiens* was within confidence intervals of the converted LC50 value reported in Ahmed and Othman.^1^ While these four species represent only 9% of the TRS toxicity set, the regression-adjusted toxicity estimates were deemed reasonable for the species and exposure durations compared.

**Table 3:** Comparison of Toxicity Data Sourced from the Literature with Regression-Adjusted Toxicity Estimates for the Same Species and Exposure Duration^a^

			toxicity value from literature			regression-adjusted toxicity value	
**species**	**end point**	**measure**	**exposure duration (days)**	toxicity value (μg/L)*	**reference**	**estimated toxicity value (μg/L)**	**reference**
Asellus aquaticus	immobilization	EC10	28	1.71 [0.386–7.55]	^48^	3.17	^65^
Gammarus pulex	immobilization	EC10	28	2.95 [1.15–7.59]	^48^	3.83	^6,65^ **
Caenis horaria	immobilization	EC10	28	0.024 [0.006–0.091]	^48^	0.034	^65^
Culex pipiens	mortality	LC10^^^	2	141 [76.8 – 161.8]^^^	^1^	135	^1^**

a * 95% confidence intervals in square brackets, ** Geomean, ^ LC50 datum and confidence intervals converted to estimates of LC10 by dividing by 5 as per Warne M St J, Batley et al.^67^

The literature-based ACR method produced a PC95 value of 0.024 μg/L, an order of magnitude lower than the 0.2 μg/L PC95 value calculated using the ANZ Guideline method with a factor of 10 conversion (Figure 4 and S6). Microcosm and mesocosm studies have illustrated significant ecosystem effects at concentrations below 0.2 μg/L (e.g.,^36^,^47^,^57^), illustrating that a default ACR of 10 may not provide sufficient protection against chronic imidacloprid exposure. The literature-derived ACRs support this, with all values being greater than or equal to 10 except for the water bug *Plea minutissima*. Mayflies generally exhibit higher sensitivity to imidacloprid than other taxa.^31,48^ The mayfly *Cloeon dipterum* exhibited the highest literature-derived ACR, calculated at 150.24, which was subsequently obscured by the 22.6 geomean value for all insects (S6). This may explain why the TRS PC95 value for 28 days of exposure was lower than, but within the confidence intervals of the PC95 derived using literature-based ACRs (S6 and S11), as the Insecta regression model was developed using a species of mayfly (*Deleatidium* sp.). This validation suggests that the TRS method reduces uncertainty when estimating the chronic effects of imidacloprid compared with the use of default ACRs. The TRS PC values are also lower than concentrations observed to cause ecosystem-level effects in micro- and mesocosm studies (S10), indicating they are likely to be protective of the ecosystem and sensitive organisms like mayflies and caddisflies.

**Figure 4:**
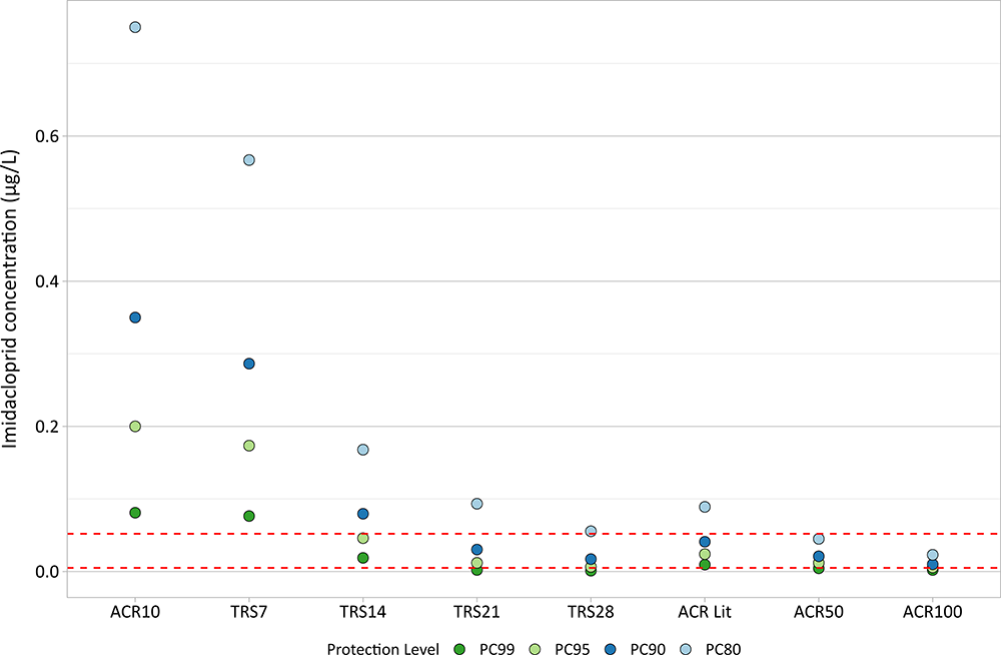
Comparison of protective concentrations (PC) derived using the TRS method at 7, 14, 21, and 28 days of exposure (TRS7, TRS14, TRS21, TRS38, respectively), and the those derived using the ACR method (ACR10, ACR Lit (literature-derived), ACR50, and ACR100). The dashed lines are the upper and lower PC95 confidence intervals derived using the highest and lowest literature-derived ACRs for each taxonomic group (see S6, Supporting Information).

For toxicants with cumulative and delayed toxicity, whether a threshold is truly protective depends on the exposure landscape (e.g., long-term or short pulses) and the protection goals (e.g., 95% protection or 99% protection) of the receiving environment.^11,28^ The SSD-based TRS method aligns with existing risk assessment frameworks for Australia^4,67^ as well as the European Water Framework Directive,^14,27^ meaning it can be used to set chronic exposure guidelines that take exposure duration into account (S11). The method can also enhance the reliability and accuracy of risk assessments when applied within a probabilistic framework, particularly if the monitoring data set is sufficiently dense to allow for estimation of exposure duration. The authors are currently working on a TRS-based risk assessment in the Great Barrier Reef catchment area using long-term and temporally dense imidacloprid monitoring data from the Great Barrier Reef Catchment Loads Monitoring Program.^71,73^ Future research may focus on the application of this method to other toxicants that display delayed or time-cumulative toxicity, such as organophosphorus insecticides, other neonicotinoid insecticides, and mercury.^21,26,43,54^ To address data limitations, future studies could focus on generating high-quality, taxa-specific timeseries bioassay data. This would enhance the regression relationships, mitigating the challenges of data scarcity and variability when deriving chronic guidelines for substances with delayed or cumulative toxicity. Additionally, future validation efforts could apply the TAFs to an SSD built solely on chronic data, as this would enable better estimation of sublethal effects. The approach also shows promise for the risk assessment of other influencing stressors (e.g., pH or water temperature). Combinations of stressors can be accommodated using a multiple linear regression approach (rather than simple linear regression) to develop the TAFs. The TRS method produces results in percent affected fraction (PAF) so can theoretically be incorporated into toxicant mixture risk assessment using a suitable model of joint toxicity (for example, Independent Action as per^68^), although this is yet to be tested.

## Supplementary Material









## Notes

S1: Summary of chronic and acute toxicity data, S2: derivation of SSD using the ANZG method, S3: Ostracoda & Maxillopoda assessment, S4: regression model development, S5: inflection point method and results, S6: protective concentrations for each ACR scenario, S7: plots and bimodality coefficients for each ACR-adjusted data set, S8: change in TRS protective concentrations over time, S9: TRS distribution plots, bimodality coefficients, and SSDs for selected exposure durations, and S10: literature review comparing the imidacloprid TRS outcomes with ecosystem effects (PDF)
S11: TRS protective concentrations (PC99/95/90/80) for days 1–100 (XLSX)
S12: Regression model toxicity data (XLSX)
S13: TRS SSD shape parameters for days 1–100 (XLSX)

## †

M.S.J.W. peacefully passed away in his sleep on February 19, 2024, at the age of 61. He served as an Associate Professor at The University of Queensland’s School of the Environment and was the Director of the Reef Catchments Science Partnership. Michael made significant and enduring contributions to science, particularly in ecotoxicology and water quality management for the Great Barrier Reef and played a key role in developing national water quality guidelines. He was not only a colleague and mentor but also a dear friend to the authors of this manuscript.
