# Supplementary material for: The Temporal Response Surface: A Novel Method for the Assessment of Delayed and Time-Cumulative Aquatic Ecosystem Risk
Source: Environ Sci Technol. 2025 May 19;59(21):10157–66. doi: 10.1021/acs.est.4c14331 (PMC12138969; doi:10.1021/acs.est.4c14331)
Supplement: Supplementary file 1 [file es4c14331_si_001.pdf]

Supplementary Information for: The Temporal Response Surface: a novel method for the assessment of delayed and time- cumulative aquatic ecosystem risk.

Cath A. Neelamraju[1,2]*, Reinier M. Mann[2,4], Michael St J. Warne[1,2†], Francisco Sanchez-Bayo[3], Ryan D.R. Turner[1,2]

1. The Reef Catchments Science Partnership, School of the Environment, The University of Queensland, St Lucia, Brisbane, Queensland, Australia, post code 4067.
2. Queensland Department of Environment, Tourism, Science and Innovation, Ecosciences Precinct, 41 Boggo Rd, Dutton Park, Brisbane, Queensland, Australia, post code 4102.
3. School of Life and Environmental Sciences, The University of Sydney, 1 Central Avenue, South Eveleigh, New South Wales, Australia, post code 2015.
4. Sustainable Minerals Institute, The University of Queensland, St Lucia, Brisbane, Queensland, Australia, post code 4067.

* Corresponding author: c.neelamraju@uq.edu.au

Summary

43 pages, 33 figures, 6 tables. References for all sections can be found on pages S39-S43.

Supporting Information

S1: Summary of chronic and acute toxicity data, S2: Derivation of SSD using the ANZG method, S3: Ostracoda & Maxillopoda assessment, S4: Regression model development, S5: Inflection point method and results, S6: Protective concentrations for each ACR scenario, S7: Plots and bimodality coefficients for each ACR-adjusted data set, S8: Change in TRS protective concentrations over time, S9: TRS distribution plots, bimodality coefficients, and SSDs for selected exposure durations, S10: Literature review comparing the imidacloprid TRS outcomes with ecosystem effects. In addition, three excel spreadsheets contain additional data. S11 (excel): TRS protective concentrations (PC99/95/90/80) for days 1-100, S12 (excel): Regression model toxicity data, S13 (excel): TRS SSD shape parameters for days 1-100.

List of tables

Table 1 **Summary of acute and chronic toxicity data used to calculate the species sensitivity distributions and protective concentrations for each imidacloprid ACR scenario, as well as the Temporal Response Surface. 5**

Table 2 The ecotoxicity threshold values (ETVs), reliability classification, and distribution parameters for the merged freshwater and marine SSD for imidacloprid using the ANZ Guideline method. All ETVs values have been rounded to two significant figures. 12

Table 3 The protective concentrations, reliability classification, and distribution parameters for the merged freshwater and marine SSD for imidacloprid (excluding Ostracoda and Maxillopoda). All values have been rounded to two significant figures. 13

Table 4 Acute to chronic ratios (ACRs) for two crustacea and seven insect species derived from values in the literature. The ACRs were geomeaned by taxonomic group. 18

Table 5 The protective concentrations, bimodality coefficients, and reliability classifications for each of the ACR-adjusted SSDs. All values are in µg/L and are rounded to two significant figures. 19

Table 6 The skewness, kurtosis, and bimodality coefficients of the temporally adjusted toxicity data in the TRS at select time points. All datasets have the same number of samples (n=35). 23

List of figures

Figure 1 Visualisation of all imidacloprid data that passed the screening and quality assessment process; (A) histogram of the pooled toxicity data, (B) kernel density plot illustrating the relative sensitivities of heterotrophs versus phototrophs, and (C) box and whiskers plot illustrating the differences in sensitivity between freshwater and marine organisms. All data have been log transformed. 10

Figure 2 Species sensitivity distribution, generated with the R package ssdtools (77), for the combined fresh and marine toxicity data for imidacloprid. This SSD was deemed slightly bimodal with non-arthropods clustered at the top end of the SSD and was therefore discarded in favour of an arthropod-only SSD. 11
Figure 3 Species sensitivity distribution, generated with the R package ssdtools (77), for the combined fresh and marine toxicity data for imidacloprid. The toxicity data used to generate this SSD can be found in S1. 12
Figure 4 Species sensitivity distribution, generated with the R package ssdtools (77), for the combined fresh and marine toxicity data for imidacloprid. Ostracoda and Maxillopoda species have been excluded from this species sensitivity distribution. 13
Figure 5 Absolute Rate of Change (ROC) in concentration over time (µg/L/day) for the three representative species. The modelled effect concentrations slowed at days 38, 88, and 94 for Deleatidium sp., H. azteca, and D. magna, respectively (marked by vertical dashed lines). 17
Figure 6 (A) a histogram showing the distribution of toxicity data used to derive the SSD for the lower Confidence Interval of the literature-derived ACR scenario, alongside (B) a density plot and (C) box and whiskers plot that illustrates the relative sensitivity of each organism Class and media type to imidacloprid. 20
Figure 7 (A) a histogram showing the distribution of toxicity data used to derive the SSD for the literature-derived ACR scenario, alongside (B) a density plot and (C) box and whiskers plot that illustrates the relative sensitivity of each organism Class and media type to imidacloprid. 21
Figure 8 (A) a histogram showing the distribution of toxicity data used to derive the SSD for the upper Confidence Interval of the literature-derived ACR scenario, alongside (B) a density plot and (C) box and whiskers plot that illustrates the relative sensitivity of each organism Class and media type to imidacloprid. 21
Figure 9 (A) a histogram showing the distribution of toxicity data used to derive the SSD for the default ACR of 50 scenario, alongside (B) a density plot and (C) box and whiskers plot that illustrates the relative sensitivity of each organism Class and media type to imidacloprid. 22
Figure 10 (A) a histogram showing the distribution of toxicity data used to derive the SSD for the default ACR of 100 scenario, alongside (B) a density plot and (C) box and whiskers plot that illustrates the relative sensitivity of each organism Class and media type to imidacloprid. 22
Figure 11. Imidacloprid protective concentrations derived from the Temporal Response Surface decrease over time. Note the y axis is in log scale which accentuates the vertical 'wobble' due to a change from log Gumbel to log normal distribution type at day 18. The analytical limit of reporting used by the Great Barrier Reef Catchment Loads Monitoring Program (87) is shown as a red dashed line. 23
Figure 12 The species sensitivity distribution, generated with the R package ssdtools (77), for the imidacloprid TRS at day 10. The dotted line is the imidacloprid concentration that is protective of 95% of the aquatic ecosystem (PC95). 24
Figure 13 (A) a histogram showing the distribution of temporally adjusted toxicity data used to derive the SSD for day 10 of the TRS, alongside (B) a density plot and (C) box and whiskers plot that illustrates the relative sensitivity of each organism Class and media type to imidacloprid. 25
Figure 14 The species sensitivity distribution, generated with the R package ssdtools (77), for the imidacloprid TRS at day 20. The dotted line is the imidacloprid concentration that is protective of 95% of the aquatic ecosystem (PC95). 25
Figure 15 (A) a histogram showing the distribution of temporally adjusted toxicity data used to derive the SSD for day 20 of the TRS, alongside (B) a density plot and (C) box and whiskers plot that illustrates the relative sensitivity of each organism Class and media type to imidacloprid. 26
Figure 16 The species sensitivity distribution, generated with the R package ssdtools (77), for the imidacloprid TRS at day 20. The dotted line is the imidacloprid concentration that is protective of 95% of the aquatic ecosystem (PC95). 26
Figure 17 (A) a histogram showing the distribution of temporally adjusted toxicity data used to derive the SSD for day 20 of the TRS, alongside (B) a density plot and (C) box and whiskers plot that illustrates the relative sensitivity of each organism Class and media type to imidacloprid. 27
Figure 18 The species sensitivity distribution, generated with the R package ssdtools (77), for the imidacloprid TRS at day 30. The dotted line is the imidacloprid concentration that is protective of 95% of the aquatic ecosystem (PC95). 27
Figure 19 (A) a histogram showing the distribution of temporally adjusted toxicity data used to derive the SSD for day 30 of the TRS, alongside (B) a density plot and (C) box and whiskers plot that illustrates the relative sensitivity of each organism Class and media type to imidacloprid. 28
Figure 20 The species sensitivity distribution, generated with the R package ssdtools (77), for the imidacloprid TRS at day 40. The dotted line is the imidacloprid concentration that is protective of 95% of the aquatic ecosystem (PC95). 28
Figure 21 (A) a histogram showing the distribution of temporally adjusted toxicity data used to derive the SSD for day 40 of the TRS, alongside (B) a density plot and (C) box and whiskers plot that illustrates the relative sensitivity of each organism Class and media type to imidacloprid. 29

Figure 22 The species sensitivity distribution, generated with the R package ssdtools (77), for the imidacloprid TRS at day 50. The dotted line is the imidacloprid concentration that is protective of 95% of the aquatic ecosystem (PC95). 29
Figure 23 (A) a histogram showing the distribution of temporally adjusted toxicity data used to derive the SSD for day 50 of the TRS, alongside (B) a density plot and (C) box and whiskers plot that illustrates the relative sensitivity of each organism Class and media type to imidacloprid. 30
Figure 24 The species sensitivity distribution, generated with the R package ssdtools (77), for the imidacloprid TRS at day 60. The dotted line is the imidacloprid concentration that is protective of 95% of the aquatic ecosystem (PC95). 30
Figure 25 (A) a histogram showing the distribution of temporally adjusted toxicity data used to derive the SSD for day 60 of the TRS, alongside (B) a density plot and (C) box and whiskers plot that illustrates the relative sensitivity of each organism Class and media type to imidacloprid. 31
Figure 26 The species sensitivity distribution, generated with the R package ssdtools (77), for the imidacloprid TRS at day 70. The dotted line is the imidacloprid concentration that is protective of 95% of the aquatic ecosystem (PC95). 31
Figure 27 (A) a histogram showing the distribution of temporally adjusted toxicity data used to derive the SSD for day 70 of the TRS, alongside (B) a density plot and (C) box and whiskers plot that illustrates the relative sensitivity of each organism Class and media type to imidacloprid. 32
Figure 28 The species sensitivity distribution, generated with the R package ssdtools (77), for the imidacloprid TRS at day 80. The dotted line is the imidacloprid concentration that is protective of 95% of the aquatic ecosystem (PC95). 32
Figure 29 (A) a histogram showing the distribution of temporally adjusted toxicity data used to derive the SSD for day 80 of the TRS, alongside (B) a density plot and (C) box and whiskers plot that illustrates the relative sensitivity of each organism Class and media type to imidacloprid. 33
Figure 30 The species sensitivity distribution, generated with the R package ssdtools (77), for the imidacloprid TRS at day 90. The dotted line is the imidacloprid concentration that is protective of 95% of the aquatic ecosystem (PC95). 33
Figure 31 (A) a histogram showing the distribution of temporally adjusted toxicity data used to derive the SSD for day 90 of the TRS, alongside (B) a density plot and (C) box and whiskers plot that illustrates the relative sensitivity of each organism Class and media type to imidacloprid. 34
Figure 32 The species sensitivity distribution, generated with the R package ssdtools (77), for the imidacloprid TRS at day 100. The dotted line is the imidacloprid concentration that is protective of 95% of the aquatic ecosystem (PC95). 34
Figure 33 (A) a histogram showing the distribution of temporally adjusted toxicity data used to derive the SSD for day 100 of the TRS, alongside (B) a density plot and (C) box and whiskers plot that illustrates the relative sensitivity of each organism Class and media type to imidacloprid. 35

S1: Toxicity data that passed the screening and quality assessment

Toxicity data were curated for the current study for four separate processes:

1. For the derivation of a chronic species sensitivity distribution (SSD) and protective concentrations for imidacloprid in line with the Australian and New Zealand Guidelines for Fresh and Marine Water Quality (79), hereafter termed the ANZ Guidelines,
2. For the development of log-log regression relationships between effect concentration and exposure duration capable of estimating chronic toxicity effects from acute toxicity data,
3. For the development of the toxicity data set underlying the Temporal Response Surface (TRS), and
4. For the derivation of acute-to-chronic ratios (ACRs) to validate the TRS.

This attachment lists the toxicity data used to derive the SSDs and protective concentration values for both the TRS and the ACR-adjusted SSDs (points 1 and 3 above). Some endpoint data were obtained as part of revision of the Australian and New Zealand Water Quality Guidelines for Fresh and Marine Water Quality (5, 34). Both freshwater and marine toxicity data that passed the quality assurance and screening procedures were used to derive the TRS and the ACR-adjusted SSDs for the current study because the pesticide reduction target in the Reef 2050 Water Quality Improvement Plan is intended to protect all freshwater and marine ecosystems of the Great Barrier Reef catchment area, estuary, and lagoon (5). As such, all aquatic species (freshwater, estuarine and marine) were theoretically included in the current study, but in practice this was limited to the species for which there were ecotoxicity data. In addition, as there were insufficient toxicity data available to build regression models for Ostracoda and Maxillopoda, organisms from these two classes were excluded from the TRS (see Table 1 footnote, and S3). Chronic toxicity data were also not used for the TRS, as converting these endpoints to estimates of acute toxicity was deemed inappropriate. While the TRS method can mathematically utilise data from any time point, this was not deemed appropriate from an ecotoxicological perspective because most chronic endpoints are either reproductive or development based. If a 28-day EC10 (reproduction) is adjusted to 4-days duration this does not make sense because reproduction generally doesn't occur at that time frame. On the other hand, an acute LC10 is still an LC10 whether it is estimated at 4-days' exposure or adjusted to a chronic 28-day exposure period. The outcome is the same (death of 10% of the test organisms). In addition, as the regression models were used to estimate toxicity in integers of whole days it was essential that the toxicity data used to build the TRS also be in whole days to avoid introduction of error into the calculations. A temporal adjustment factor was applied to one of the toxicity data points prior to development of the TRS, as the duration of the toxicity test for this species was 4.75 days (35). The toxicity datum for *Gammarus lawrencianus* (Malacostraca) was therefore adjusted to an exposure duration of 5 days using the *Hyalella* regression model. Data quality was assessed using the nationally endorsed ANZ Guideline method, which examines how each toxicity value was generated and awards a quality score on the basis of answers to a series of questions (see Table 3 and Appendix 1 of 79). According to the ANZG method, only 'high' (≥80%) and 'acceptable' (≥50% to <80%) quality toxicity data may be used to derive water quality guidelines. For the acute toxicity data (i.e., the TRS toxicity set), 48.7% were of acceptable quality and 51.3% were of high quality. The lowest quality score was 61.5% for *H.azteca* (47) which was geomeaned with two other toxicity values that scored 73% (71) and 87.9% (57). The maximum quality score was 92.6% which was recorded for both *Americamysis bahia* and *Penaeus japonicus* from a study by Hano, Ito, et al. (22). The median quality score for all TRS toxicity data was 82.4%. The data quality assessment spreadsheets can be made available if requested.

Table 1 Summary of acute and chronic toxicity data used to calculate the species sensitivity distributions and protective concentrations for each imidacloprid ACR scenario, as well as the Temporal Response Surface.

Organism Type	Species Scientific Name	Phylum	Media	Class	Hetero/ Phototroph	LOWEST VALUE FOR SPECIES (ug/L)	Exposure duration (days)	Data type	Endpoint	Measure	Reference
Macroinvertebrate	*Amphibalanus amphitrite*	Arthropoda	Marine	Maxillopoda	Crustacean	1660^	4	Chronic NOEC	Larval development	Larval development (%)	Negri, Templeman, et al. (49)
Microinvertebrate	*Ceriodaphnia dubia*	Arthropoda	Fresh	Branchiopoda	Crustacean	2040†^	7	Chronic NOEC/EC10	Reproduction	Neonates/female	Raby, Nowierski, et al. (57)
Macroinvertebrate	*Chironomus dilutus*	Arthropoda	Fresh	Insecta	Insect	0.06^	40	Chronic EC20	Adult emergence	Adult emergence	Cavallaro, Morrissey, et al. (15)
Macroinvertebrate	*Chironomus riparius*	Arthropoda	Fresh	Insecta	Insect	0.37†^	28	Chronic NOEC	Development	Emergence time (50% individuals)	Njattuvetty Chandran, Fojtova, et al. (50)
Macroinvertebrate	*Coenobita variabilis*	Arthropoda	Marine	Malacostraca	Crustacean	43*^	4.75	Chronic EC10	Larval development	Larval development (%)	Negri, Templeman, et al. (49)
Macroinvertebrate	*Daphnia magna*	Arthropoda	Fresh	Branchiopoda	Crustacean	1800^	21	Chronic NOEC	Immobilisation	Immobilisation	USEPA (76)
Macroinvertebrate	*Neocloeon triangulifer*	Arthropoda	Fresh	Insecta	Insect	1.12^	32	Chronic EC10	Reproduction	Emergence	Raby, Zhao, et al. (58)
Macroinvertebrate	*Protonemura sp.*	Arthropoda	Fresh	Insecta	Insect	8.47†^	21	Chronic NOEC/EC10	Development	Emergence	Brüggemann, Hund-Rinke, et al. (11)
Macroinvertebrate	*Aedes sp.*	Arthropoda	Fresh	Insecta	Insect	4.08a	2	Acute LC50	Mortality	Mortality	Raby, Nowierski, et al. (57)
Macroinvertebrate	*Americamysis bahia*	Arthropoda	Marine	Malacostraca	Crustacean	7.770†a	4	Acute LC50	Mortality	Mortality	Hano, Ito, et al. (22), USEPA (77)
Macroinvertebrate	*Asellus aquaticus*	Arthropoda	Fresh	Malacostraca	Crustacean	7.8a	4	Acute EC50	Immobilisation	Immobilisation	Van den Brink, Van Smeden, et al. (78)
Macroinvertebrate	*Baetis rhodani*	Arthropoda	Fresh	Insecta	Insect	0.849a	2	Acute LC50	Mortality	Mortality	Beketov and Liess (9)
Macroinvertebrate	*Caecidotea sp.*	Arthropoda	Fresh	Malacostraca	Crustacean	32.08a	4	Acute EC50	Immobilisation	Immobilisation	Raby, Nowierski, et al. (57)

Organism Type	Species Scientific Name	Phylum	Media	Class	Hetero/ Phototroph	LOWEST VALUE FOR SPECIES (ug/L)	Exposure duration (days)	Data type	Endpoint	Measure	Reference
Macroinvertebrate	*Caenis horaria*	Arthropoda	Fresh	Insecta	Insect	0.6a	4	Acute EC50	Immobilisation	Immobilisation	Van den Brink, Van Smeden, et al. (78)
Macroinvertebrate	*Callinectes sapidus*	Arthropoda	Marine	Malacostraca	Crustacean	10.566†a	1	Acute LC50	Mortality	Mortality	Osterberg (53)
Macroinvertebrate	*Chaoborus obscuripes*	Arthropoda	Fresh	Insecta	Insect	325.8a	4	Acute EC50	Immobilisation	Immobilisation	Van den Brink, Van Smeden, et al. (78)
Macroinvertebrate	*Cheumatopsyche brevilineata*	Arthropoda	Fresh	Insecta	Insect	0.485a	2	Acute EC50	Immobilisation	Immobilisation	Yokoyama, Ohtsu, et al. (87)
Macroinvertebrate	*Chironomus tentans*	Arthropoda	Fresh	Insecta	Insect	0.575a	4	Acute LC50	Mortality	Mortality	Stoughton, Liber, et al. (71)
Microinvertebrate	*Chydorus sphaericus*	Arthropoda	Fresh	Branchiopoda	Crustacean	220.9a	2	Acute EC50	Immobilisation	Immobilisation	Sánchez-Bayo and Goka (65)
Macroinvertebrate	*Cloeon sp.*	Arthropoda	Fresh	Insecta	Insect	2.31a	4	Acute EC50	Immobilisation	Immobilisation	Raby, Nowierski, et al. (57)
Macroinvertebrate	*Coenagrion sp.*	Arthropoda	Fresh	Insecta	Insect	346.27a	4	Acute LC50	Mortality	Mortality	Raby, Nowierski, et al. (57)
Macroinvertebrate	*Crangon uritai*	Arthropoda	Marine	Malacostraca	Crustacean	57a	4	Acute EC50	Immobilisation	Immobilisation	Hano, Ito, et al. (22)
Macroinvertebrate	*Culex pipiens*	Arthropoda	Fresh	Insecta	Insect	32.2†a	3	Acute LC50	Mortality	Mortality	Ahmed and Othman (2)
Microinvertebrate	*Cypretta seurati*	Arthropoda	Fresh	Ostracoda	Crustacean	1.6a^	2	Acute EC50	Immobilisation	Immobilisation	Sánchez-Bayo and Goka (65)
Microinvertebrate	*Cypridopsis vidua*	Arthropoda	Fresh	Ostracoda	Crustacean	0.3a^	2	Acute EC50	Immobilisation	Immobilisation	Sánchez-Bayo and Goka (65)
Macroinvertebrate	*Ephemerella sp.*	Arthropoda	Fresh	Insecta	Insect	1.06a	4	Acute EC50	Immobilisation	Immobilisation	Raby, Nowierski, et al. (57)
Macroinvertebrate	*Gammarus fossarum*	Arthropoda	Fresh	Malacostraca	Crustacean	25.56a	1	Acute EC50	Immobilisation	Immobilisation	Malev, Klobučar, et al. (42)

Organism Type	Species Scientific Name	Phylum	Media	Class	Hetero/ Phototroph	LOWEST VALUE FOR SPECIES (ug/L)	Exposure duration (days)	Data type	Endpoint	Measure	Reference
Macroinvertebrate	*Gammarus lawrencianus*	Arthropoda	Marine	Malacostraca	Crustacean	0.554 a**	4.75	Acute EC50	Immobilisation	Immobilisation	Knysh, Courtenay, et al. (35)
Macroinvertebrate	*Gammarus pulex*	Arthropoda	Fresh	Malacostraca	Crustacean	8.02†a	4	Acute EC50	Immobilisation	Immobilisation	Ashauer, Hintermeister, et al. (6), Van den Brink, Van Smeden, et al. (78)
Macroinvertebrate	*Gyrinus sp.*	Arthropoda	Fresh	Insecta	Insect	5.75a	4	Acute EC50	Immobilisation	Immobilisation	Raby, Nowierski, et al. (57)
Macroinvertebrate	*Hexagenia sp.*	Arthropoda	Fresh	Insecta	Insect	932.05a	4	Acute LC50	Mortality	Mortality	Raby, Nowierski, et al. (57)
Macroinvertebrate	*Hyalella azteca*	Arthropoda	Fresh	Malacostraca	Crustacean	4.39†a	4	Acute LC50	Mortality	Mortality	USEPA (76), Minton (47), Stoughton, Liber, et al. (71), Raby, Nowierski, et al. (57)
Microinvertebrate	*Ilyocypris dentifera*	Arthropoda	Fresh	Ostracoda	Crustacean	0.3a^	2	Acute EC50	Immobilisation	Immobilisation	Sánchez-Bayo and Goka (65)
Macroinvertebrate	*Isonychia bicolor*	Arthropoda	Fresh	Insecta	Insect	1.89†a	4	Acute EC50	Immobilisation	Immobilisation	Camp and Buchwalter (14), Raby, Nowierski, et al. (57)
Macroinvertebrate	*MacCaffertium sp.*	Arthropoda	Fresh	Insecta	Insect	1.06a	4	Acute EC50	Immobilisation	Immobilisation	Raby, Nowierski, et al. (57)
Macroinvertebrate	*Micrasema sp.*	Arthropoda	Fresh	Insecta	Insect	0.64a	4	Acute EC50	Immobilisation	Immobilisation	Raby, Nowierski, et al. (57)
Macroinvertebrate	*Palaemonetes pugio*	Arthropoda	Marine	Malacostraca	Crustacean	41.71†a	4	Acute LC50	Mortality	Mortality	Key, Chung, et al. (33)
Macroinvertebrate	*Penaeus japonicus*	Arthropoda	Marine	Malacostraca	Crustacean	5a	4	Acute EC50	Immobilisation	Immobilisation	Hano, Ito, et al. (22)
Macroinvertebrate	*Plea minutissima*	Arthropoda	Fresh	Insecta	Insect	18.9a	4	Acute EC50	Immobilisation	Immobilisation	Van den Brink, Van Smeden, et al. (78)
Macroinvertebrate	*Simulium latigonium*	Arthropoda	Fresh	Insecta	Insect	0.373a	4	Acute LC50	Mortality	Mortality	Beketov and Liess (9)

Organism Type	Species Scientific Name	Phylum	Media	Class	Hetero/ Phototroph	LOWEST VALUE FOR SPECIES (ug/L)	Exposure duration (days)	Data type	Endpoint	Measure	Reference
Macroinvertebrate	*Simulium vittatum*	Arthropoda	Fresh	Insecta	Insect	0.81†a	2	Acute LC50s	Mortality	Mortality	Overmyer, Mason, et al. (54)
Macroinvertebrate	*Stenelmis sp.*	Arthropoda	Fresh	Insecta	Insect	9.92a	4	Acute EC50	Immobilisation	Immobilisation	Raby, Nowierski, et al. (57)
Macroinvertebrate	*Trichocorixa sp.*	Arthropoda	Fresh	Insecta	Insect	6.31a	2	Acute EC50	Immobilisation	Immobilisation	Raby, Nowierski, et al. (57)

^ Data not used in TRS, † Geomean, [a] Values were acute LC50/EC50 values that were converted to chronic negligible effect concentration values by dividing by 10, as per the ANZ Guideline method (79), * Value of 41.405 used for TRS (predicted effect concentration at 5 days using Hyalella regression model), ** Value of 0.5296 used for TRS (predicted effect concentration at 5 days using Hyalella regression model).

S2: Construction of a chronic toxicity SSD for the derivation of guidelines for use in Australia and New Zealand (default ACR of 10)

Two SSD calculation software packages were considered for this project; Burrlioz (12) because this is the software currently recommended by the Australian & New Zealand Guidelines for Fresh & Marine Water Quality (79), and ssdtools (73) because this is what will be prescribed by the Guidelines in the near future (18, 19). Burrlioz is an R Shiny-based software application while ssdtools is available as both Shiny interface (16) and as an R code package in CRAN (73). Both Burrlioz 2.0 and ssdtools fit species sensitivity distributions to concentration data; however, the latter platform provides additional functionality that was useful for the current study. This functionality includes the ability to fit and compare a broad range of distribution types at the same time, the provision of test statistics to assess goodness-of-fit, enhanced data visualisation, model prediction capability, and confidence interval generation. In addition, being an R-based package, it was possible to standardise and automate the data visualisation, SSD generation, and data output processes. This allowed for a reduction in error as well as improved computational efficiency in the current study.

In total, there were toxicity data for 43 arthropod species (five classes within one phyla) and 14 non-arthropod species (seven classes across six phyla) that passed the screening and quality assessment process. Given the current understanding of neonicotinoid mode of action and the type and sensitivity of the nAChR in arthropods (insects and crustaceans), it is expected that this group of organisms are more sensitive to the effects of imidacloprid than non-arthropods (e.g., fish) (48, 55, 75). A modality assessment of the pooled arthropod and non-arthropod toxicity data supported this theory. Firstly, a bimodality coefficient was calculated to be above the 0.555 threshold specified in Warne M St J, Batley, et al. (79). Furthermore, a visual inspection of the data and statistical test for difference both indicated that the data distribution was bimodal, with arthropods being more sensitive to imidacloprid than non-arthropods (S2, Supplementary Information). Modality was assessed using the combined toxicity dataset for all freshwater and marine species that passed the screening and quality assessment process, to increase the sample size being assessed (n = 57). All data that were not chronic NOEC or EC10 values were first converted to this type of data as per the methods recommended by Warne M St J, Batley, et al. (79), then transformed using a natural logarithm (ln) to normalise the data for visualisation and testing. A bimodality coefficient of 0.521, calculated on the log-transformed data, suggested that the data may exhibit bimodality as it is close to (although under) the >0.555 cutoff specified in Warne M St J, Batley, et al. (79). A visual examination of the data histogram (Figure 1A) kernel density plot (Figure 1B), and box and whiskers plot (Figure 1C) showed that although arthropods are generally more sensitive to imidacloprid exposure, some overlap exists between the two groups. A statistical analysis of the pooled toxicity data found no difference in sensitivity between fresh and marine organisms ($p>0.05$); however, arthropods were significantly more sensitive to imidacloprid exposure than non-arthropods ($p<0.00005$). As the modality assessment was inconclusive, both organism types were initially considered for the derivation of the merged fresh and marine SSD for imidacloprid and a decision made based on the fit of the data to the SSD.

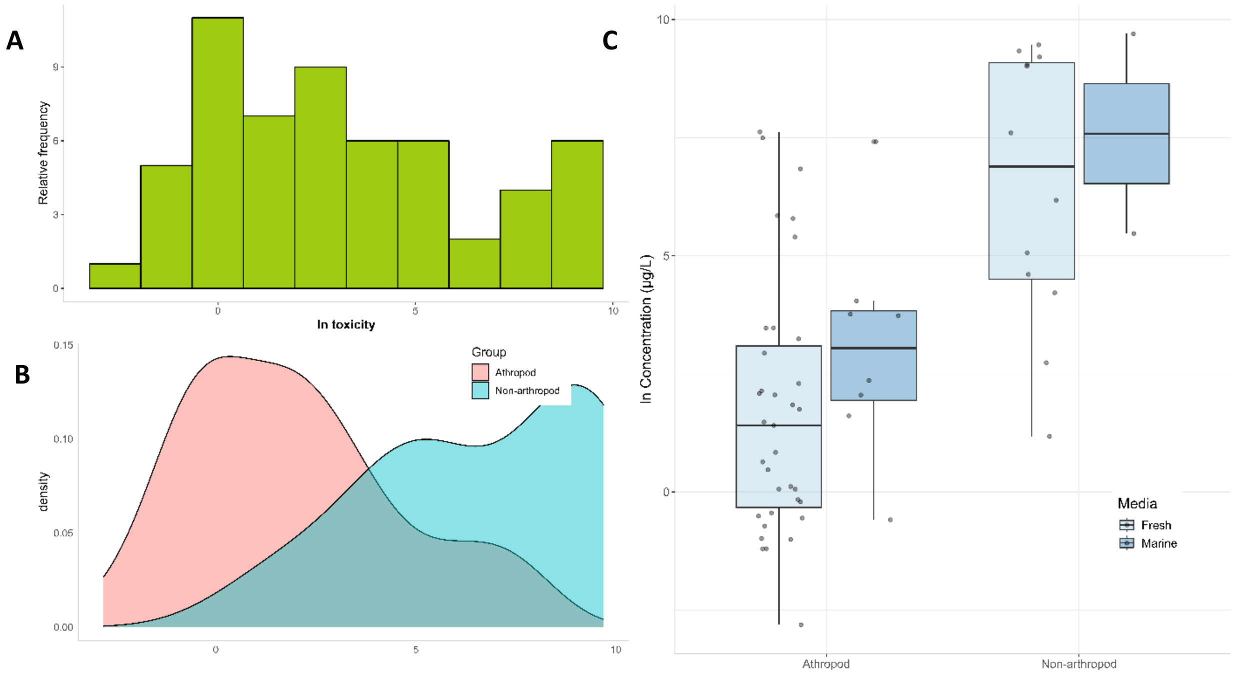

Figure 1 Visualisation of all imidacloprid data that passed the screening and quality assessment process; (A) histogram of the pooled toxicity data, (B) kernel density plot illustrating the relative sensitivities of heterotrophs versus phototrophs, and (C) box and whiskers plot illustrating the differences in sensitivity between freshwater and marine organisms. All data have been log transformed.

For the pooled data set (both arthropods and non-arthropods), there were chronic NOEC/EC10 type data (data preference 1) for 10 freshwater species and two marine species. For freshwater, there was a further one chronic EC/LC50 type toxicity datum (data preference 2) and 37 converted acute EC/LC50 type data (data preference 4). For marine there were a further seven converted acute EC/LC50 type data (data preference 4). To derive the merged fresh and marine imidacloprid SSD, the acute toxicity data (data preference 4) were first converted to chronic negligible effect concentration values by dividing by 10, as per the ANZ Guideline method (79). Then chronic NOEC/EC10, chronic EC/LC50, and the converted acute data were combined for both arthropods and non-arthropods, creating a data set of 57 species across seven phyla. However, the resulting SSD fit appeared bimodal, with non-arthropods at the top end of the SSD, producing a poor fit (Figure 2). The concentrations likely to protect 99%, 95%, and 90% of the ecosystem (PC99, PC95, and PC90, respectively) were much the same as those previously published for freshwater and marine (separate guidelines) by King, Smith, et al. (34). However, the concentration likely to protect 80% of the ecosystem (the PC80) was approximately three times higher (1.3 µg/L in the current study compared to 0.3 µg/L for freshwater and 0.46 µg/L for marine in King, Smith, et al. (34).

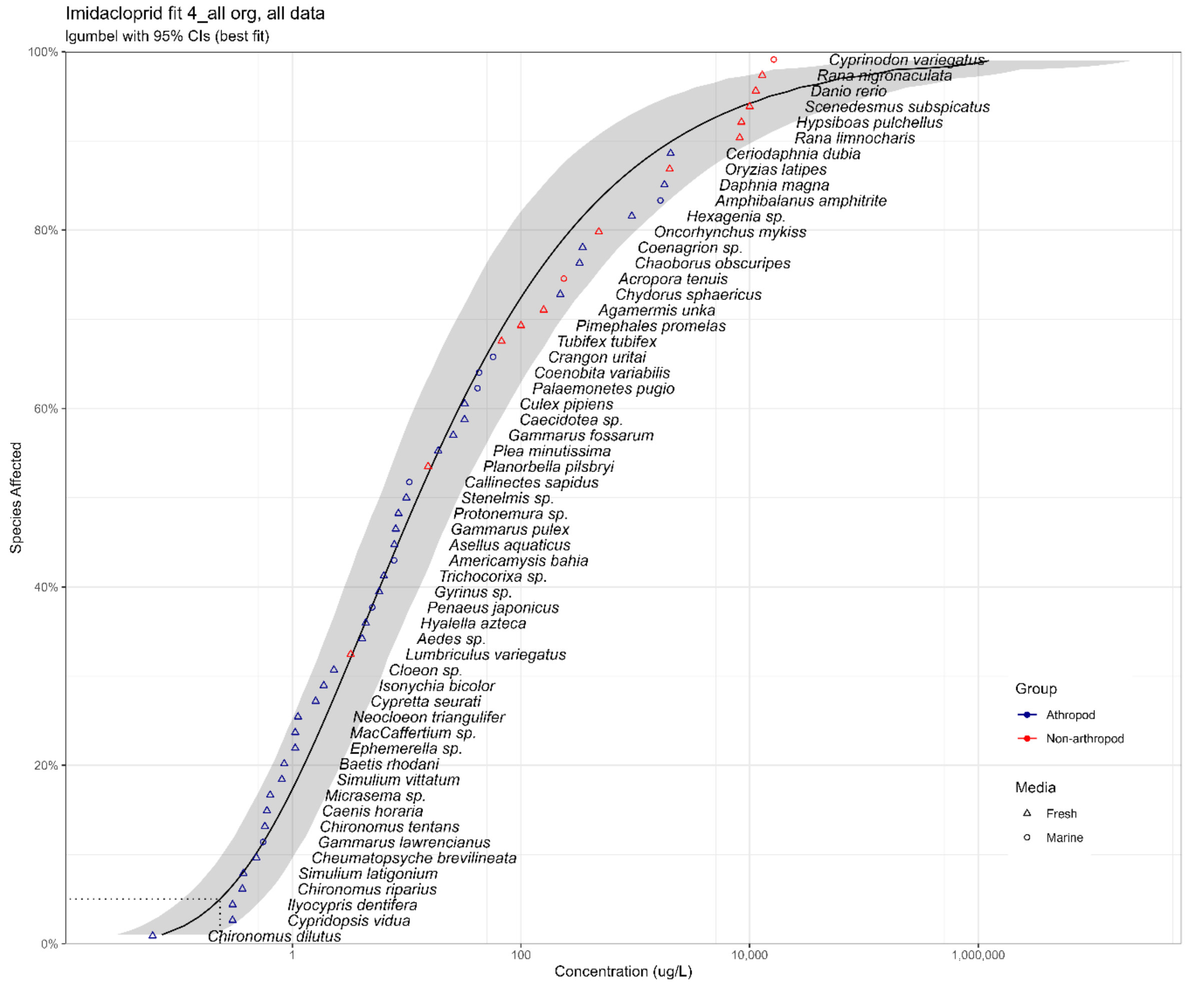

Figure 2 Species sensitivity distribution, generated with the R package ssdtools (73), for the combined fresh and marine toxicity data for imidacloprid. This SSD was deemed slightly bimodal with non-arthropods clustered at the top end of the SSD and was therefore discarded in favour of an arthropod-only SSD.

A second arthropod-only SSD was developed using all data types available (data preference 1 and 4) in order to satisfy minimum data requirements. When an SSD is derived using species from a single phylum (in this case, arthropods), the necessity for data from at least four taxonomic groups can be balanced by ensuring a good fit for the SSD and obtaining reliable PC values. This approach is acceptable if the overall dataset for the chemical still includes at least five species from four different phyla (79). This SSD was deemed a good fit to the data with moderate reliability, and protective concentrations very similar to those previously published for fresh and marine ecosystems (34). Therefore, this SSD and the resulting protective concentrations are likely to be more protective of sensitive organisms (arthropods). The represented classes were Branchiopoda (water fleas), Insecta (insects), Malacostraca (crabs, lobsters, and shrimp), Maxillopoda (barnacles), and Ostracoda (seed shrimp). The merged fresh and marine imidacloprid SSD derived using the ANZ Guideline method (default ACR of 10) is presented in Figure 3.

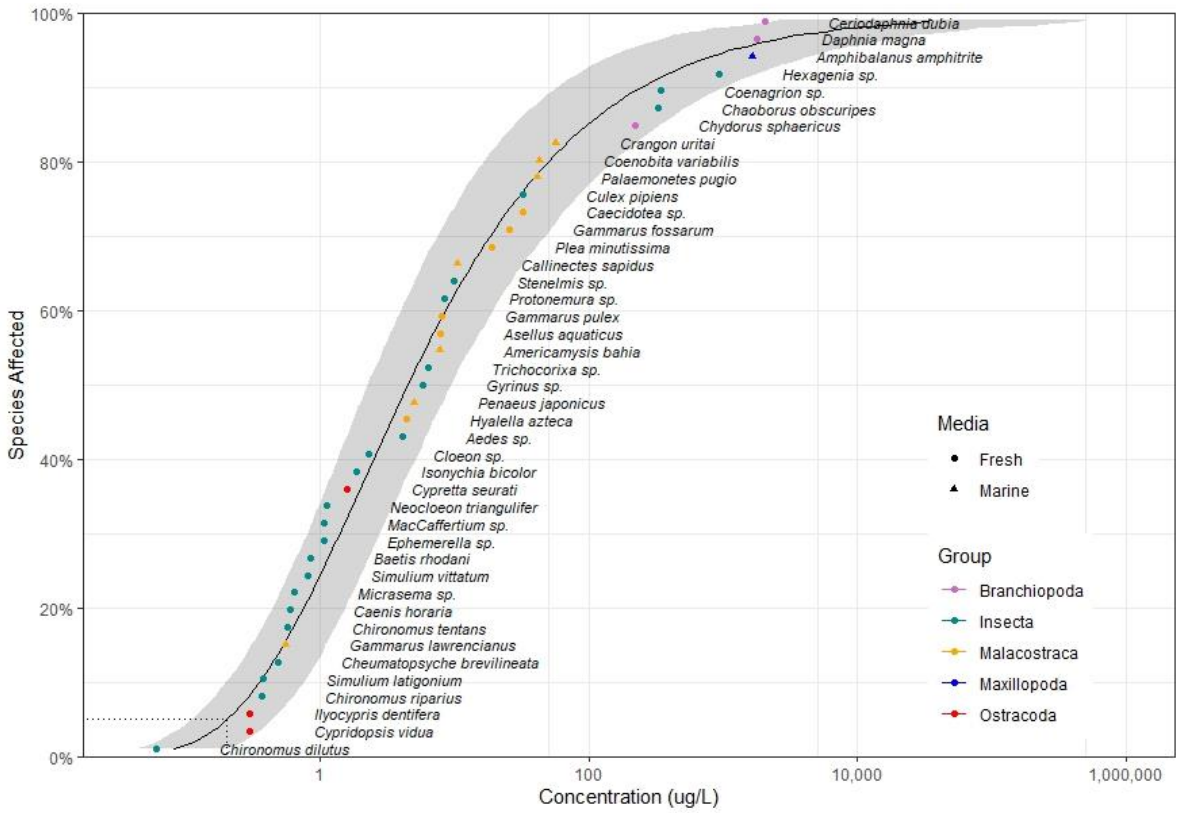

Figure 3 Species sensitivity distribution, generated with the R package ssdtools (73), for the combined fresh and marine toxicity data for imidacloprid. The toxicity data used to generate this SSD can be found in S1.

The distribution parameters, DGVs, and reliability classification of the merged fresh and marine imidacloprid SSD (Figure 3) are presented in Table 2. The reliability of the PC values was assessed based on the type and quality of the data, the sample size, and a visual estimation of SSD goodness-of-fit, to assign a reliability classification to each SSD (79).

Table 2 The ecotoxicity threshold values (ETVs), reliability classification, and distribution parameters for the merged freshwater and marine SSD for imidacloprid using the ANZ Guideline method. All ETVs values have been rounded to two significant figures.

Ecotoxicity threshold values	
**Protection level**	**Concentration (µg/L)**
PC99	0.081
PC95	0.20
PC90	0.35
PC80	0.75

Reliability classification*	
**Criterion**	**Result**
Sample size	43
Type of toxicity data	Combined chronic and converted acute for fresh and marine
SSD model fit	Good
Reliability	Moderate

Distribution parameters	
**SSD parameters**	**Values**
Distribution type	Log Gumbel
location	0.7289
scale	2.1237

* refer to Warne M St J, Batley, et al. (79) for definitions of each criterion.

S3: Testing the influence of Ostracoda and Maxillopoda species

The merged fresh and marine SSD (data preference 1 and 4) was recalculated without the four Ostracoda and Maxillopoda species in order to investigate the effect that removing these organisms would have on the protective concentration (PC) values. The distribution parameters, PC values, and reliability classification of the merged fresh and marine imidacloprid toxicity data (excluding Ostracoda and Maxillopoda) are presented in Table 3 and can be compared with those in Table 2. The SSD for the combined fresh and marine imidacloprid (excluding Ostracoda and Maxillopoda) toxicity is presented in Figure 4. As the values for Ostracoda and Maxillopoda fall within the range of the other organisms on the SSD (see Figure 3) they do not appear to influence the SSD distribution type or shape when removed. Both SSD distributions with (Table 2) and without (Table 3) these species are log Gumbel, the distribution parameters are similar, and the PC values are also comparable. The fit for both SSDs was determined to be good, with Moderate reliability PC values for both methods, based on the reliability classification of Warne M St J, Batley, et al. (79). Therefore, exclusion of Ostracoda and Maxillopoda from the imidacloprid SSD does not appear to impact the level of protection that will be provided by the imidacloprid Temporal Response Surface.

Table 3 The protective concentrations, reliability classification, and distribution parameters for the merged freshwater and marine SSD for imidacloprid (excluding Ostracoda and Maxillopoda). All values have been rounded to two significant figures.

Protective concentrations	
**Protection levels**	**Protective concentrations (µg/L)**
PC99	0.094
PC95	0.23
PC90	0.40
PC80	0.85

Reliability classification*	
**Criterion**	**Result**
Sample size	39
Type of toxicity data	chronic NOEC/EC10, chronic EC20 data, and converted acute data for both fresh and marine species
SSD model fit	Good
Reliability	Moderate

Distribution parameters	
**SSD parameters**	**Values**
Distribution type	Log Gumbel
location	0.84
scale	2.09

* refer to Warne M St J, Batley, et al. (79) for definitions of each criterion.

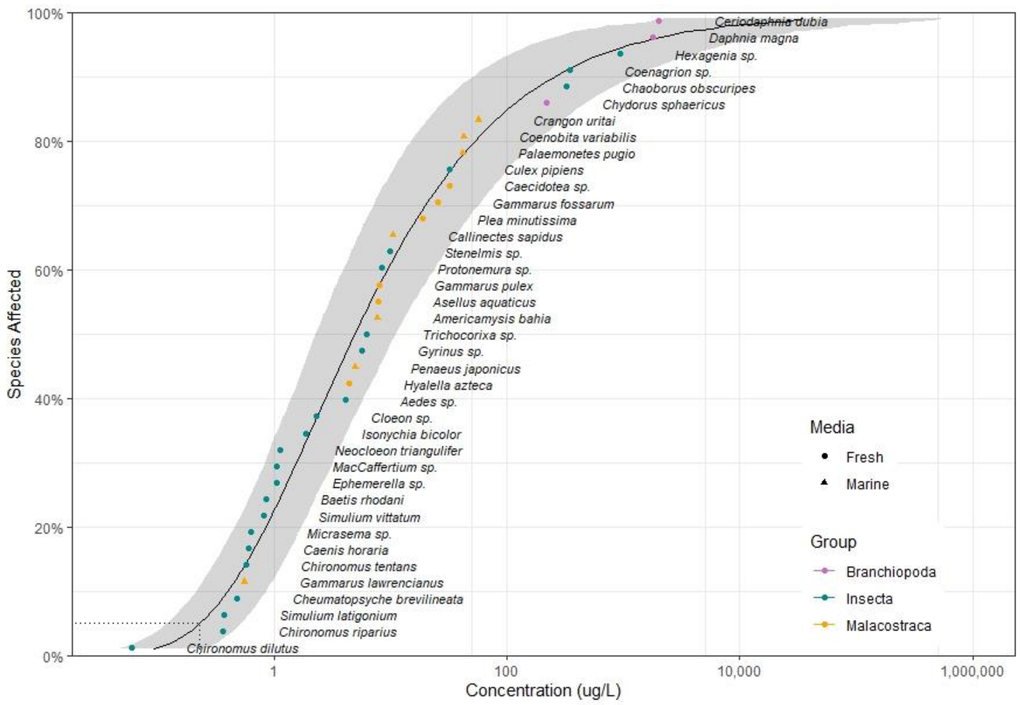

Figure 4 Species sensitivity distribution, generated with the R package ssdtools (73), for the combined fresh and marine toxicity data for imidacloprid. Ostracoda and Maxillopoda species have been excluded from this species sensitivity distribution.

S4: Development of log-log regression relationships

The use of regression analysis to predict chronic lethality using time series acute bioassay data has been around for some time and is well-published. For example, Sprague (70) details the linear relationship that exists between the log of organism survival time and logged dose of a toxicant, and how this may be used to identify lethal threshold concentrations of toxicants. Green (21) employed a regression relationship between the LC50 and exposure duration over short time frames (up to 2 days) to estimate the appropriate test duration needed to assess chronic toxicity of salinity in the amphipod *Pontoporeia affinis*. Mayer, Krause, et al. (45) developed regression relationships between acute effect concentration, degree of response, and duration of exposure that were able to predict chronic lethality 84% to 92% of the time when the approach was applied to a database of 18 chemicals and seven fish species. Sánchez-Bayo (63) used log-log regression analysis to uncover the patterns of time-cumulative toxicity in neonicotinoid insecticides. Kumar, Correll, et al. (36) developed log-log regression relationships between exposure duration and effect concentration that produced credible predictions of chronic lethality for six different pesticides to the freshwater shrimp, *Paratya australiensis*. Three of these pesticides were insecticides known to exhibit cumulative or delayed toxicity characteristics: chlorpyrifos and dimethoate (both organophosphorus insecticides), and cypermethrin (a synthetic pyrethroid) (41, 60). They further propose that predictions of chronic toxicity from time series regression can be used in ecological risk assessments with reasonable confidence where no measured estimates of chronic toxicity are available.

Due to variations introduced by differences in experimental design, toxicity data were preferentially selected if they were presented in the same paper or at least determined in the same laboratory (79). The selected data were mortality endpoints of at least 50% effect as per the methods of Sánchez-Bayo and Tennekes (67). LC/EC50 data tend to have smaller standard error compared to other measures, e.g., LC/EC10, and therefore produce more stable estimates. Toxicity datasets with sufficient temporally spaced endpoint statistics (i.e. EC/LC50) were identified for three species: the mayfly *Deleatidium* sp. (39, 40), the freshwater amphipod *Hyalella azteca* (7, 57), and the water flea *Daphnia magna* (30). All toxicity data passed the quality assessment process outlined in Warne M St J, Batley, et al. (79), with the exception of *Deleatidium* sp., which had poor survivorship in the control treatments in one of the papers (40). The *Deleatidium* sp. data were still used to build the Insecta regression relationship; however, we recommend that this process be repeated once better-quality data becomes available. See section on *Deleatidium* sp. for discussion. All three data sets displayed less variance in the predictor (exposure duration) than the response (effect concentration) so a gaussian generalised linear model (GLM) was applied to the natural log of the two variables using the following formula:

$$lnEC = \beta \times lnED + \varepsilon \qquad \textit{Equation 1}$$

where $lnEC$ refers to the natural log of the effect concentration in µg/L, β is the regression slope estimate, $lnED$ is the natural log of the exposure duration in whole days, and ε is the residual error. The log-log regression models for each species (*Daphnia magna, Deleatidium* sp., and *Hyalella azteca*) were developed separately, employing an iterative approach to assess goodness-of-fit, ensure model integrity, and address any influential points. Influential points were defined as observations in the data that, once removed, improved the strength and predictive power of the model. Influential points were identified using the DFFITS ("difference in fits") metric and Cooke's distance, which is a method of identifying outliers based on the scaled change in fitted model values. As there were insufficient data to allow for splitting into separate training and test sets, the predictive performance of the regression models was estimated using the leave-one-out-cross-validation (LOOCV) method (86). In LOOCV, the dataset is iteratively split into a training and test set, with one observation left out of the test set for each iteration. The model is then fitted to the remaining data and used to estimate the value of the data point that has been removed. This process is repeated until each observation has been used for both training and validation exactly once. The Mean Square Error (MSE) is calculated for each iteration then averaged across all iterations to provide an unbiased estimate of model performance. An average MSE close to zero indicates that the model predictions are highly accurate with minimal error between the observed and predicted values (31).

Daphnia magna

There were initially eight data points for *D. magna* across two mortality-related endpoints, all from the one study (30). The *D. magna* data set included two LC50 (effect concentration of imidacloprid causing 50% mortality) data points

calculated at 5 and 7 days using a logistic dose-response relationship. There was also one ET50 (time to 50% mortality) data point calculated for each of the 6 concentrations tested, using the hyperbolic model method described by Sánchez-Bayo (63). This resulted in six ET50 data points with various exposure durations between 6 and 9.39 days. The EC50 and ET50 data were combined to create the dataset of 8 data points. All data were logged and a gaussian GLM with identity link applied. Assessment of the diagnostic plots, DFFITS and Cooks distance revealed the two EC50 data points to be outliers influencing the model, and these were subsequently removed. With these data removed, the D-squared value for the *D. magna* model increased from 0.52 to 0.99 and overall model diagnostics improved. The average MSE, estimated using LOOCV, was 0.00049. The model was therefore making predictions very close to observed values, indicating good predictive performance. The final *D. magna* model therefore included six data points taken at multiple time intervals between 6 and 9.39 days (Figure 2, main paper). Modelled ET50 effect concentrations from days 1 to 365 show the rate of change in toxicity begins to slow at approximately 94 days of continuous exposure (indicated as an inflection point in Figure 5 below).

Deleatidium sp.

There were initially 23 *Deleatidium* sp. EC50 or LC50 data points across three different effects (behaviour, intoxication, and mortality) from two separate studies by the same lead author (39, 40). Both studies were conducted on the same species, in artificial soft water, with organisms of the same age (juveniles 10 mm in length). However, one of the studies (39) was a 4-day acute bioassay, with static application of three imidacloprid concentration treatments between 0 and 40.5 µg/L and the other (40) was a 28-day chronic toxicity bioassay, with a static-renewal design and ten imidacloprid concentration treatments ranging from 0 to 4 µg/L. The chronic toxicity bioassay produced data for two sub-lethal endpoints (impairment and immobilization), as well as mortality. The authors define impairment as being 'unable to right themselves by performing a normal swimming movement, but were still able to walk' and ascertained that this sub-lethal response is the first part of the mortality continuum; i.e. all dead nymphs were assumed to have previously been impaired, then immobilized (40). However, a comparison of the regression slopes for each endpoint revealed that data belonging to the impairment endpoint had a significantly different slope (i.e. a different relationship between exposure duration and toxicity) to those of the immobilization and mortality data ($p < 0.001$). Therefore, all data for the behaviour endpoint were removed, leaving 16 data points for analysis.

All toxicity data passed the quality assessment process outlined in Warne M St J, Batley, et al. (79) with the exception of Macaulay, Hageman, et al. (40), which had poor survivorship in the control treatments. Statistical correction was not applied to these data by the study authors prior to estimation of LCx values (*pers comms.* Sam Macaulay 15/04/2025) which may have resulted in an overestimation of the toxicity of imidacloprid to *Deleatidium* sp. These data were still used to build the Insecta regression relationship for several reasons. Firstly, the control mortality was reasonably consistent between time points, so it is likely that all LCx values were overestimated to a similar magnitude. For generation of TAFs for the current study, it is the slope of the regression relationship (i.e., the speed at which imidacloprid becomes more toxic) that is most important, not the toxicity estimates themselves. While the lack of statistical correction (e.g. Abbotts formula) may have impacted the individual estimated LCx values, the difference between them (slope) should be reasonably reliable. This theory was tested by estimating the slope of the relationship between exposure duration and effect concentration for other mayfly species using Equation 1. A review of available literature uncovered sufficient high quality data to determine a regression slope for two other mayfly species. It is important to note that these data were paired time points (e.g., an estimate of toxicity on day 4 then again on day 10 of imidacloprid exposure) not time series data (estimates of toxicity at successive time points such as 4, 7, 14, and 28 days), so could not be used to derive regression relationships for the calculation of TAFs. The data were used to provide an estimate of the change in toxicity over time (as slope) similar to the use of acute-to-chronic ratios which are also based on paired toxicity values. For *Chironomus riparius*, the LC50 effect concentration was found to decline with a slope of -0.831 between 1 and 10 days of imidacloprid exposure (50, paper quality score 95.6%). For *Chironomus dilutus*, the LC50 also declined quickly with a slope of -1.895 between 4 days (57, paper quality score 90.1%) and 10 days (47, paper quality score 72.1%) of imidacloprid exposure. For the current study, the slope of the log-log regression relationship for *Deleatidium* sp. was found to be -1.827 (see Table 1 of main manuscript). This is close to the estimate for *C. dilutus* and is therefore deemed a reasonable representation of the increase in toxicity over time for sensitive taxa such as mayflies. Secondly, there were no other time-series data available for class Insecta. In order to retain these organisms in the SSD they would need to have been adjusted using either the Branchiopoda regression model (slope

of -3.982) or the Malacostraca regression model (slope of -0.737). This may have resulted in an overestimation or underestimation, respectively, of the change in toxicity over time for Class Insecta. In addition, the *Deleatidium* sp. regression model produced reasonable temporally adjusted estimates when applied to two other Insecta species (see Table 3 of main manuscript). The TRS toxicity data set included a 96-hour EC10 (Immobolisation) data point for *Caenis horaria* from Van den Brink, Van Smeden, et al. (78). When adjusted to 28 days, the estimated toxicity fell within the confidence intervals of the same species, exposure duration, and endpoint reported by Roessink, Merga, et al. (62). The 96-hour *Culex pipiens* LC10 (Mortality) estimate of Ahmed and Othman (2), when adjusted to 48 hours, aligned with the confidence intervals of the 48-hour LC10 reported in the same study. Finally, the regression relationships introduced here offer a novel advancement in methods to adjust acute toxicity data to estimates of chronic toxicity. Existing approaches often rely on literature-derived ACRs, which may vary in quality (see Table 4 of S6), or arbitrary default ACRs, which can significantly over- or underestimate toxicity changes over time. Like any new methodology, refinements will be necessary in future iterations. To address data limitations, future studies could focus on generating high-quality, taxa-specific timeseries bioassay data. This would enhance SSD adjustments for problematic toxicants, mitigating the challenges of data scarcity and variability when deriving chronic guidelines for substances with delayed or cumulative toxicity. We therefore deemed the *Deleatidium* sp. data suitable for the current study but recommend that this process be repeated once better-quality data becomes available.

The 16 data points were logged and a gaussian GLM with identity link applied. Assessment of the diagnostic plots, DFFITS and Cookes distance revealed two data points to be outliers influencing the model. On investigation, these two points both had an exposure duration of 4 days, both were from the same paper (39), and of a slightly different experimental design than the remaining data (40). With these data removed, the D-squared value for the *Deleatidium* sp. model increased from 0.91 to 0.97 and overall model diagnostics improved. The average MSE, estimated using LOOCV, was 0.037. The model was therefore making predictions very close to observed values, indicating good predictive performance. The final *Deleatidium* sp. model therefore included 15 LC50 and EC50 data points across multiple time intervals between 7 and 28 days (Figure 2, main paper). Modelled LC50 or EC50 effect concentrations from days 1 to 365 show the rate of change in toxicity begins to slow at approximately 38 days of continuous exposure (indicated as an inflection point in Figure 5 below).

Hyalella azteca

The initial *H. azteca* data set contained seven LC50 data points spanning 2 to 28 days, from four different studies (7, 57, 71, 76). All studies were conducted in fresh water with juvenile amphipods between 2 and 10 days old as test organisms. However, the model fit for this pooled data set was poor (D-squared of 0.06). Investigation of diagnostic plots, DFFITS and Cookes distance revealed data points from two of the studies (71, 76) to be outliers. These two values were removed, leaving five LC50 data points spanning 4 to 28 days from two studies (7, 57). According to OECD guidelines, the use of five data points is acceptable for the determination of a statistical relationship between a measured response (e.g. LC50) and the concentration of the analyte in a sample (52). The two studies adhered to all other OECD guideline requirements such as test concentrations over a dynamic range of toxicity, at least 20 organisms per concentration, and control mortality within acceptable ranges. One of these studies was acute in nature with a test duration of 4 days (57), while the other was a chronic bioassay test spanning 28 days (7). The data from these two studies were logged and a gaussian GLM with identity link applied. The D-squared value for this model was 0.99, and the model diagnostics were acceptable. The average MSE, estimated using LOOCV, was 0.0085. The model was therefore making predictions very close to observed values, indicating good predictive performance. The final *H. azteca* model therefore included five LC50 data points taken at multiple time intervals between 4 and 28 days (Figure 2, main paper). Modelled LC50 effect concentrations from days 1 to 365 show the rate of change in toxicity begins to slow at approximately 88 days of continuous exposure (indicated as an inflection point in Figure 5 below).

S5: Calculation of inflection points in the regression models

While each of the three representative species has been shown to live up to a year under optimal conditions (29, 56, 84), the extrapolation of statistical relationships derived from relatively short durations (9.39 days for *D. magna*, 28 each for *Deleatidium* sp. and *H. azteca*) to much longer durations (365 days) will carry inherent uncertainty. Also, as the exposure time becomes sufficiently long, effect concentration data tends to approach an asymptotic value (21, 70). This means that the modelled effect concentration data will form an exponential decay curve once unlogged; while never

truly reaching zero, there will be a point of inflection in the unlogged modelled data where the rate of change between successive time points reduces to almost nothing. After this point, there is little benefit in analysing the change in toxicity, as there will be very little impact on the protective concentrations of the final TRS model.

Inflection points were calculated by analysing the rate of change (ROC) between successive modelled estimates to determine when the slope of the unlogged regression curve was slowing down (i.e. plateauing). The difference in ROC was determined for each of the three modelled species over the course of 1 to 365 days using the following formula.

$$Difference\ in\ ROC\ at\ time_i = \frac{\Delta(effect\ concentration)}{\Delta(exposure\ duration)} \qquad \textit{Equation 2}$$

Where Δ (delta) represents the change or difference between two consecutive observations. A rolling average window equivalent to the bioassay duration for each of modelled species was employed to assess this rate of change (9.39 days for *D. magna*, 28 each for *Deleatidium* sp. and *H. azteca*), and a cutoff value of 0.01 µg/L was established to identify significant inflection points. This cutoff value aligns with half of the analytical limit of reporting for imidacloprid employed by the Great Barrier Reef Catchment Loads Monitoring Program (83). Below this limit of reporting the aqueous concentration of imidacloprid cannot be accurately quantified, and therefore any change in sensitivity of modelled organisms below this level also cannot be accurately quantified. The change in modelled effect concentration slows at days 38, 88, and 94 for *Deleatidium* sp., *H. azteca*, and *D. magna*, respectively (indicated as vertical dashed lines for each species in Figure 5). It is important to note that the shape of the regression relationships (and location of inflection points) are species- and model-specific, and this time span may change if the method is applied to other species or toxicants.

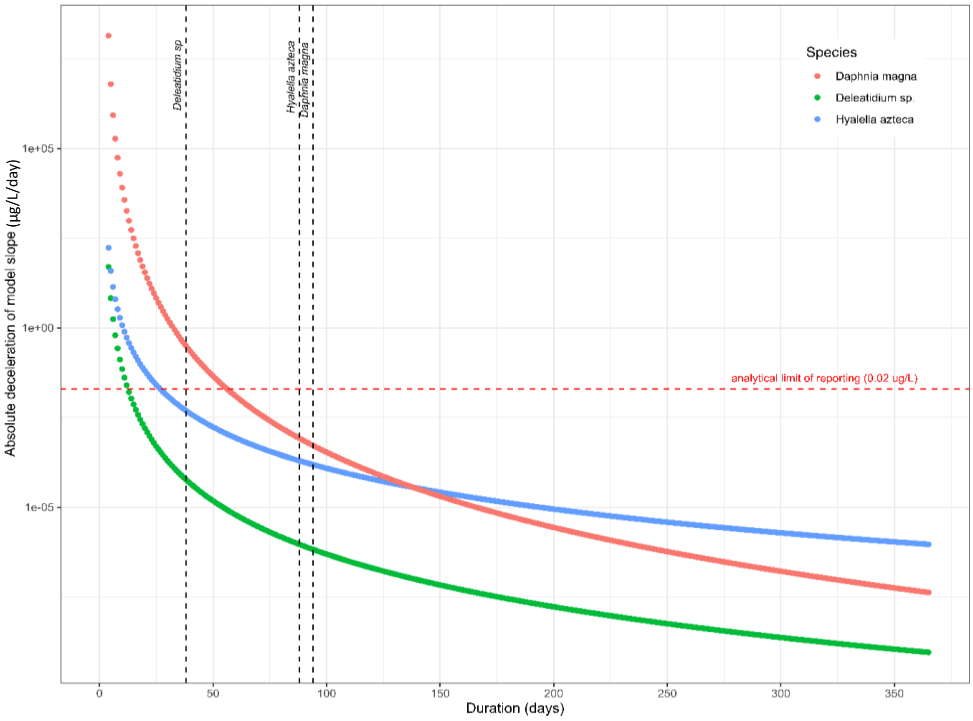

Figure 5 Absolute Rate of Change (ROC) in concentration over time (µg/L/day) for the three representative species. The modelled effect concentrations slowed at days 38, 88, and 94 for Deleatidium sp., H. azteca, and D. magna, respectively (marked by vertical dashed lines).

S6: Calculation of acute to chronic ratios and PC values

The ANZ Guidelines recommend the use of ACRs to convert acute toxicity data to an estimate of chronic toxicity (8, 79). Acute to chronic ratios represent the relationship between the acute toxicity (LC50 or EC50) and chronic toxicity (NOEC or EC10) of a particular chemical. Due to variation in ACRs between species and toxicants, Warne M St J, Batley, et al. (79) recommend that the data used to derive an ACR be for the same species and presented in the same paper, or at least determined in the same laboratory. The guidelines permit the application of ACRs derived for one species (or a geomean if several ACRs are available) to all species in an SSD to derive chronic water quality guidelines (79). However, the extrapolation from acute data to an estimate of chronic toxicity using ACRs can be problematic due to variability in sensitivity between species and toxicants (13, 81). For example, Ahlers, Riedhammer, et al. (1) compiled acute and chronic toxicity data for green algae (102 substances), *Daphnia magna* (102 substances), and fish (32 substances) and found that while the median ACRs for fish, *D. magna* and algae were reasonably low (10.5, 7, and 5.4, respectively), ACRs for individual species and chemicals varied greatly, with the highest being 4,400. They found no statistical relationship between ACR and log K_{OW} (a measure of a substance's hydrophobicity), nor ACR and trophic level of each organism. Raimondo, Montague, et al. (59) compiled 456 paired acute and chronic toxicity data points for aquatic invertebrates and fish for a range of metals, narcotics, pesticides, and other organic chemicals. They found that while the median ACR across all species was 8.3, there was a 16,000-fold range between the lowest and highest ACR values. These results have been replicated in other studies (e.g. 26, 44). Therefore, the application of ACRs to convert acute toxicity data to estimates of chronic toxicity may result in somewhat arbitrary values that may be under-protective (or over-protective) depending on the species and toxicant in question. The limitations of default ACRs are further discussed in Warne M St J, Westbury, et al. (81).

However, to assess the suitability of using regression relationships to adjust the acute toxicity data, it was necessary to derive SSDs using the ACR method for comparison. A literature review was conducted which identified suitable data for the derivation of nine species-specific imidacloprid ACRs, which were calculated using the following formula:

$$ACR = \frac{acute\ LC10\ or\ EC10}{chronic\ LC10\ or\ EC10} \qquad \text{Equation 3}$$

Three studies from the open literature had suitable data for the derivation of species-specific imidacloprid ACRs in two crustaceans and seven insect species (Table 4). None of these data passed the quality screening processes outlined in Warne M St J, Batley, et al. (79) due to either low chemical purity, use of formulation products, or high mortality in the control treatments, so the resulting protective concentrations could not be used for derivation of chronic water quality guidelines for imidacloprid. However, these ACRs should approximate the actual increase in toxicity, and were therefore deemed suitable for validation and sanity-checking of protective concentrations derived using the TRS method. For crustaceans, the ACRs ranged from 17.24 for *Gammarus pulex* to 45.63 for *Asellus aquaticus.* For insects, the minimum calculated ACR was 7.43 for *Plea minutissima,* while the maximum was 150.24 for *Cloeon dipterum.* For each taxon, a geomean ACR of 28.1 and 22.6 for crustacea and insects, respectively, was determined. The geomean ACRs were then applied to the SSD toxicity data for each species according to taxonomic group. As an additional measure, the highest and lowest ACR from each taxonomic group were used to derive upper and lower confidence bounds for the ACR-adjusted SSDs and the protective concentrations derived using this approach.

Table 4 Acute to chronic ratios (ACRs) for two crustacea and seven insect species derived from values in the literature. The ACRs were geomeaned by taxonomic group.

Taxonomic group	Species	Endpoint	Measure	ACR	Chemical purity	Ref	ACR Geomean
Crustacea	*Asellus aquaticus*	Mortality	96h LC10/ 28d LC10	45.63*	20% (formulation)	(62)	28.1
	Gammarus pulex	Mortality	96h LC10/ 28d LC10	17.24*	20% (formulation)	(62)	
Insecta	*Chaoborus obscuripes*	Mortality	96h LC10/ 28d LC10	89.45*	20% (formulation)	(62)	22.6
	Sialis lutaria	Immobilisation	96h EC10/ 28d EC10	12.27^#	20% (formulation)	(62)#	
	Plea minutissima	Mortality	96h LC10/ 28d LC10	7.43	20% (formulation)	(62)	
	Cloeon dipterum	Mortality	96h LC10/ 28d LC10	150.24	20% (formulation)	(62)	

Taxonomic group	Species	Endpoint	Measure	ACR	Chemical purity	Ref	ACR Geomean
	Caenis horaria	Mortality	96h LC10/ 28d LC10	10.85*	20% (formulation)	(62)	
	Chironomus tentans	Mortality	96h LC10/ 28d LC10[a]	31.59*	99.2% for acute, 24% (formulation) for chronic	(71)**	
	Deleatidium sp.	Immobility	168h EC10[a]/ 28d EC10[a]	10*	>99% (analytical grade)	(40)	

* Because the control mortality in these tests exceeded 10% these results should be considered indicative only. ** Value not included in Insecta geomean as the chronic 8-d LC10 was derived using an imidacloprid formulation (~24% purity), therefore the calculated ACR may not be representative of actual change in effect between the two time points. ^ acute EC10 value is ">" so the calculated ACR is indicative only. # The ACR for *Sialis lutaria* was calculated using the Immobility endpoint due to large confidence intervals on LC10 data, [a] value converted from EC50 or LC50 to EC10 or LC10 by dividing by 5.

In addition to the literature-derived ACRs, several default ACRs were also applied in order to derive SSDs and protective concentrations for comparison with the TRS. In the absence of species- and chemical-specific ACRs, the ANZ Guidelines recommend the application of a conversion factor of 10 to convert acute LC/EC/IC/50 values to estimates of chronic EC10/NOEC values (79). However, Warne M St J, Westbury, et al. (81) cite a study by Kenaga (32) who compiled acute and chronic data for 84 chemicals, including pesticides for nine species of fish and two invertebrates. From this dataset, 135 ACR values were derived that ranged from 1 to 18,100. The authors concluded that protective concentrations derived using a default ACR of 10 would underestimate the chronic toxicity of 57% of the chemicals studied. Calabrese and Baldwin (13) recommend using an ACR value of 50 to overcome this issue should no suitable species- and chemical-specific ACR be available. May, Drost, et al. (44), who calculated fish and *D. magna* ACRs for 203 different substances, recommend the use of a default ACR of 100 which they found to be sufficiently protective for 90% of the chemicals they studied. Therefore, the following three default ACRs were also applied to convert acute toxicity data to estimates of chronic toxicity for the current study:

1. A default ACR of 10, as recommended by the ANZ Guidelines (79),
2. A default ACR of 50, as recommended by Calabrese and Baldwin (13); and
3. A default ACR of 100, as recommended by May, Drost, et al. (44).

The resulting converted acute data were combined with existing chronic toxicity data to derive separate ACR-adjusted SSDs and protective concentrations for scenarios 1-3 above. The imidacloprid concentrations that are protective of 99%, 95%, 90% and 80% (PC99, PC95, PC90, and PC80, respectively) of the ecosystem for each ACR-adjusted scenario are presented in Table 5. The ACR-adjusted SSDs for all scenarios were deemed a good fit to the data, and the resulting protective concentrations were categorised as being of Moderate Reliability (79). A summary of the acute and chronic toxicity data prior to ACR adjustment, including exposure durations and conversions used, can be found in S1.

Table 5 The protective concentrations, bimodality coefficients, and reliability classifications for each of the ACR-adjusted SSDs. All values are in µg/L and are rounded to two significant figures.

	**Scenario**			
Protection level	ACR of 10 (ANZG)	ACRs from literature [upper and lower confidence intervals]*	ACR of 50	ACR of 100
PC99	0.081	0.0094 [0.0015-0.043]	0.0044	0.0021
PC95	0.20	0.024 [0.0040-0.11]	0.012	0.0056
PC90	0.35	0.041 [0.0073-0.19]	0.021	0.010
PC80	0.75	0.089 [0.017-0.41]	0.045	0.023
Treatment	ACR of 10 applied to all acute toxicity data	ACRs from literature, applied to acute toxicity data by taxonomic group	ACR of 50 applied to all acute toxicity data	ACR of 100 applied to all acute toxicity data
Bimodality coefficient	0.521	0.569	0.574	0.586

* PC values for the upper and lower confidence intervals were derived by applying the lowest and highest ACRs, respectively, for each taxonomic group in Table 4 to the acute toxicity data in S1.

S7: ACR scenario modality check

The relative sensitivity of each organism Class to imidacloprid was assessed visually using box and whisker plots and frequency histograms, for each of the ACR-adjusted scenarios in S6. The bimodality coefficient (BC), an estimate of the empirical relationship between bimodality and the skewness and kurtosis of a dataset, was also calculated on log-transformed data using the following formula (79):

$$BC = \frac{\gamma^2+1}{K+\frac{3(n-1)^2}{(n-2)(n-3)}} \quad \textit{Equation 4}$$

where γ is skewness, K is excess kurtosis (adjusted for normality), and *n* is the sample size. A BC greater than 0.555 suggests the data are not uniform and may be bimodal (20, 79).

Scenario: Lower CI ACR from literature

BC = 0.559, data are not normally distributed (p <0.005)

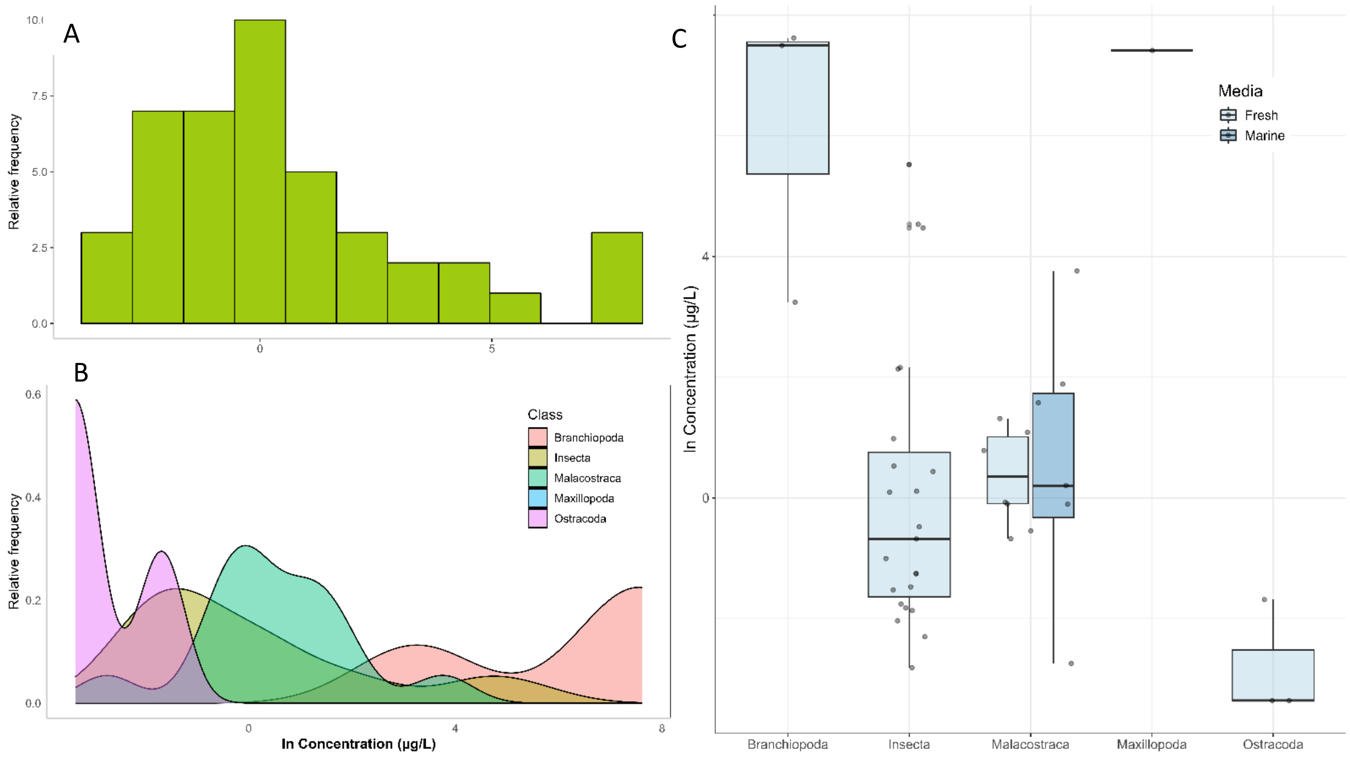

Figure 6 (A) a histogram showing the distribution of toxicity data used to derive the SSD for the lower Confidence Interval of the literature-derived ACR scenario, alongside (B) a density plot and (C) box and whiskers plot that illustrates the relative sensitivity of each organism Class and media type to imidacloprid.

Scenario: ACR from literature

BC = 0.659, data are not normally distributed ($p < 0.05$)

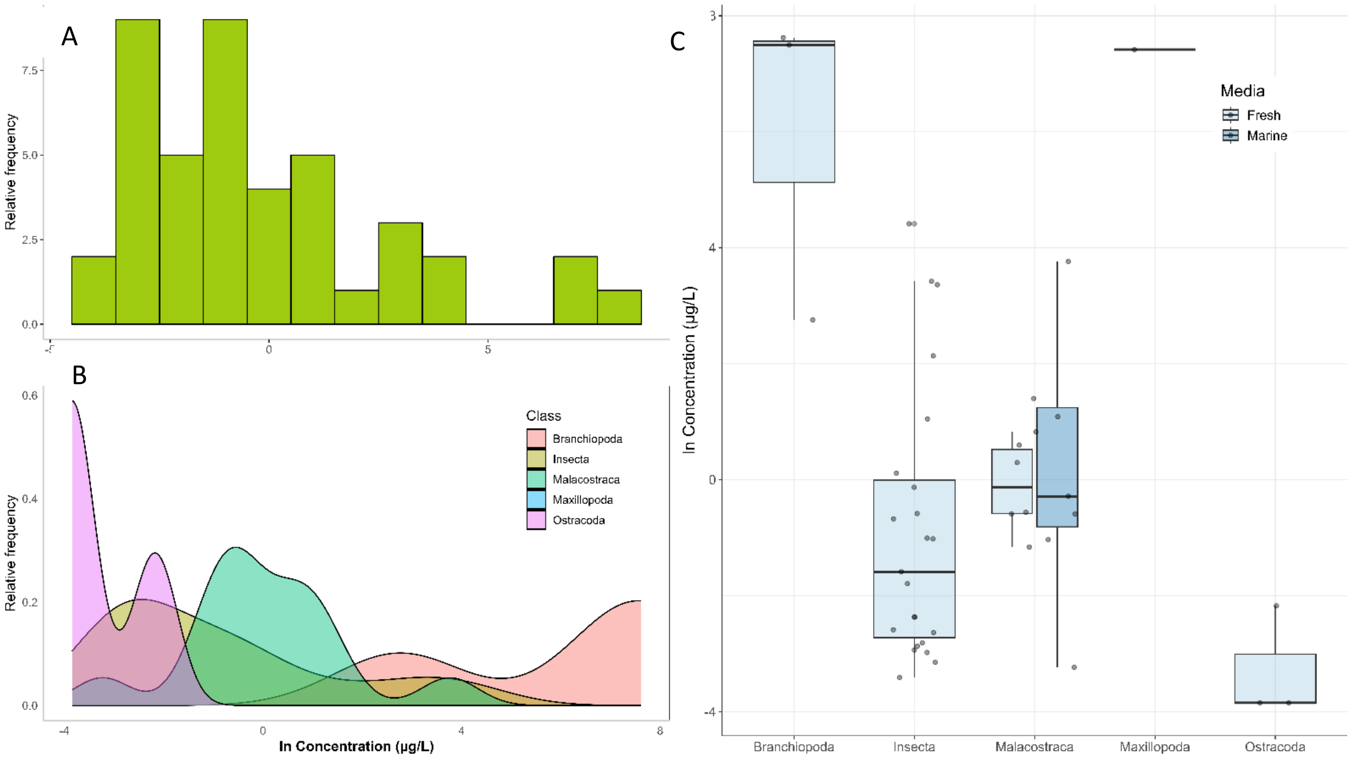

Figure 7 (A) a histogram showing the distribution of toxicity data used to derive the SSD for the literature-derived ACR scenario, alongside (B) a density plot and (C) box and whiskers plot that illustrates the relative sensitivity of each organism Class and media type to imidacloprid.

Scenario: Upper ACR from literature

BC = 0.532, data are not normally distributed ($p < 0.05$)

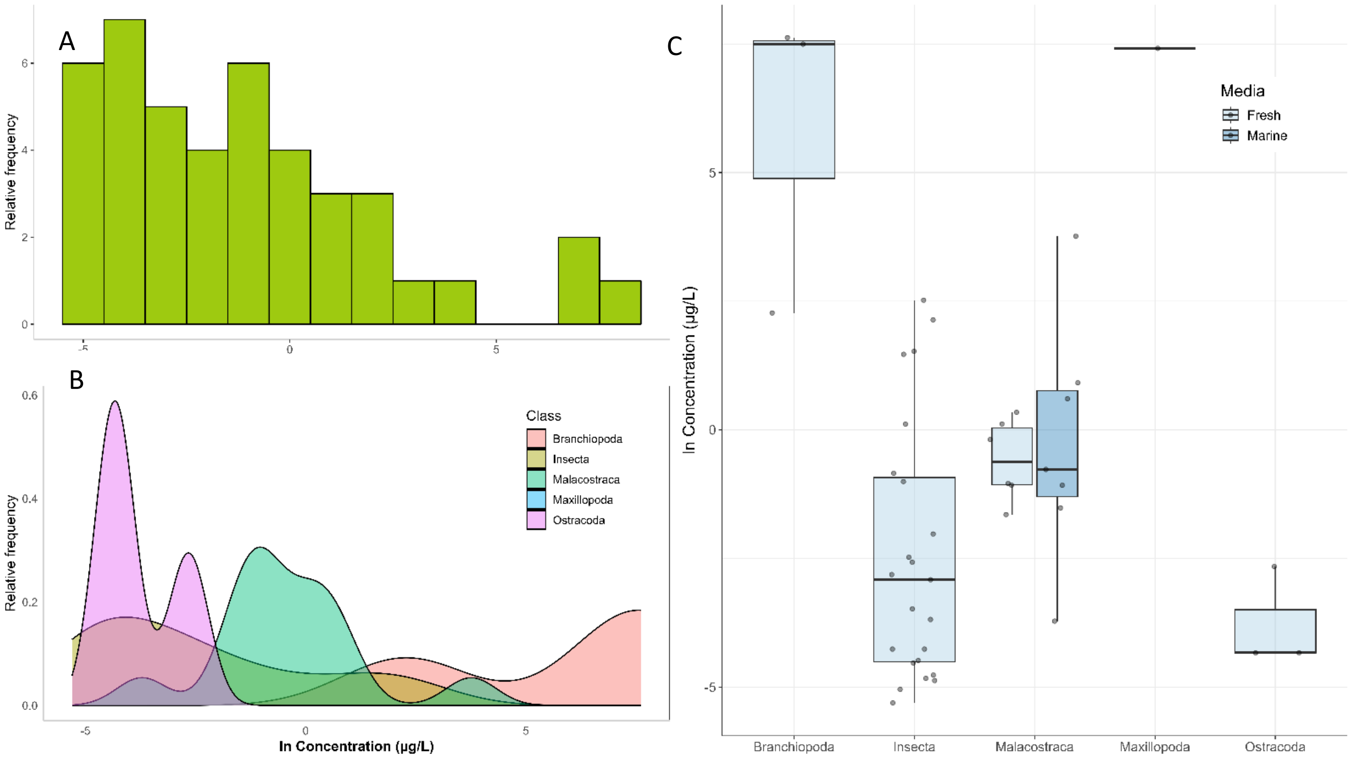

Figure 8 (A) a histogram showing the distribution of toxicity data used to derive the SSD for the upper Confidence Interval of the literature-derived ACR scenario, alongside (B) a density plot and (C) box and whiskers plot that illustrates the relative sensitivity of each organism Class and media type to imidacloprid.

Scenario: Default ACR of 50

BC = 0.574, data are not normally distributed (p <0.005)

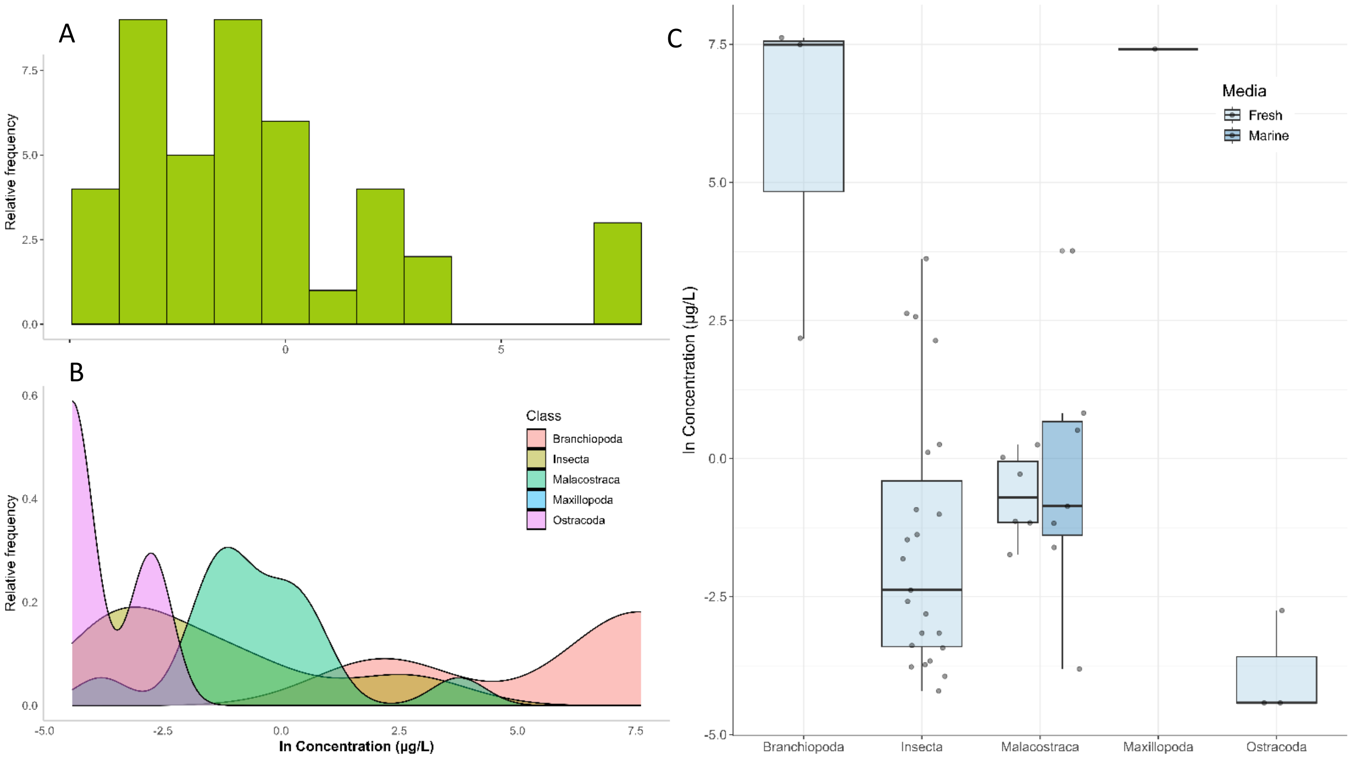

Figure 9 (A) a histogram showing the distribution of toxicity data used to derive the SSD for the default ACR of 50 scenario, alongside (B) a density plot and (C) box and whiskers plot that illustrates the relative sensitivity of each organism Class and media type to imidacloprid.

Scenario: Default ACR of 100

BC = 0.586, data are not normally distributed (p <0.001)

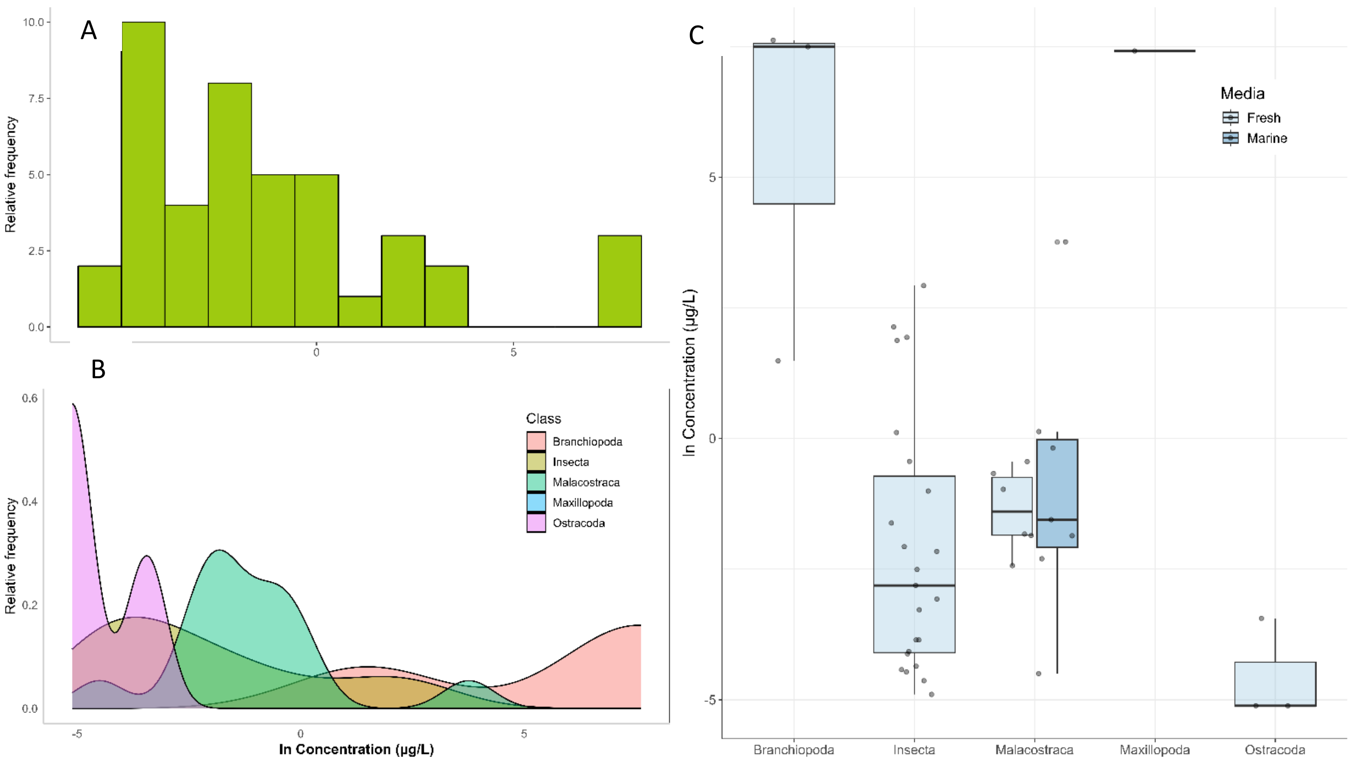

Figure 10 (A) a histogram showing the distribution of toxicity data used to derive the SSD for the default ACR of 100 scenario, alongside (B) a density plot and (C) box and whiskers plot that illustrates the relative sensitivity of each organism Class and media type to imidacloprid.

S8: Change in TRS protective concentrations over time

Analysis of the TRS shows that the imidacloprid concentrations that are protective of 99%, 95%, 90% and 80% (PC99, PC95, PC90, and PC80, respectively) of the ecosystem drop quickly within the first 30 days of continuous exposure and become progressively lower over the extended 100-day exposure period (Figure 11).

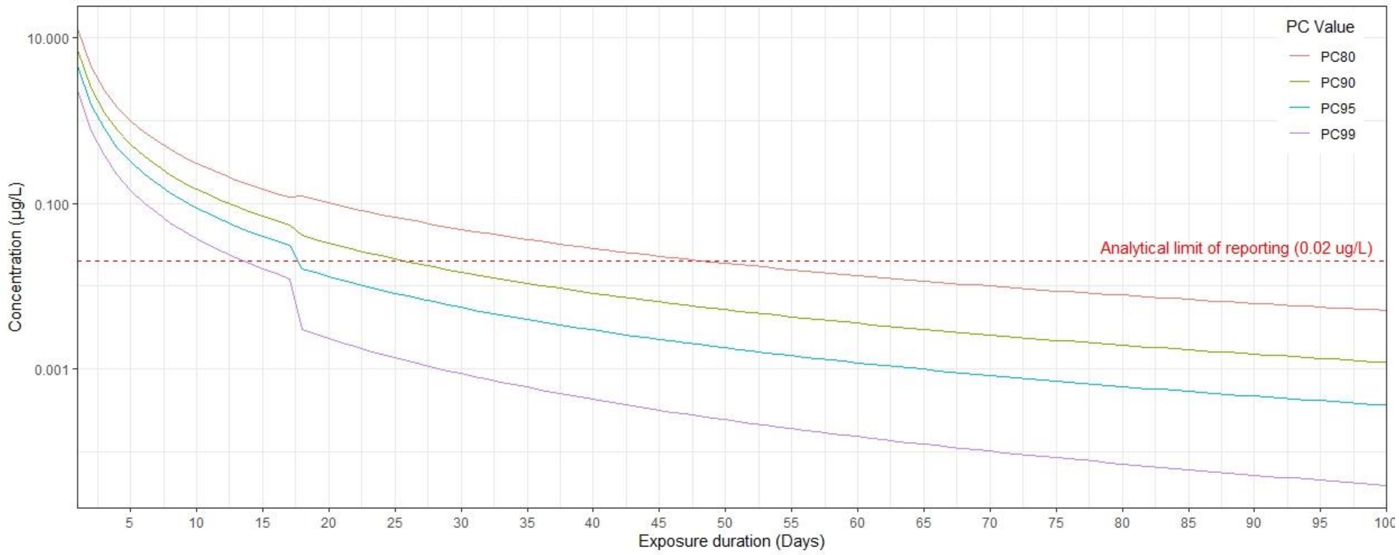

Figure 11. Imidacloprid protective concentrations derived from the Temporal Response Surface decrease over time. Note the y axis is in log scale which accentuates the vertical 'wobble' due to a change from log Gumbel to log normal distribution type at day 18. The analytical limit of reporting used by the Great Barrier Reef Catchment Loads Monitoring Program (83) is shown as a red dashed line.

S9: TRS modality check, SSDs and data visualization

Due to changes in the relative sensitivity of organisms over time, the distribution type that best fitted each temporally adjusted SSD also changed over time. Therefore, the weight-of-evidence approach (79) was used to conduct modality tests of the temporally adjusted toxicity data at select time points. The adjusted toxicity data were tested for bimodality at 10-day intervals from day 10 to day 100 of exposure. The bimodality coefficient (BC) was under the <0.555 cut off specified in Warne M St J, Batley, et al. (79) for all time points tested (Table 6) and a visual inspection of the data also suggested the data were not bimodal. Therefore, the temporally adjusted toxicity data were deemed to be unimodal.

Table 6 The skewness, kurtosis, and bimodality coefficients of the temporally adjusted toxicity data in the TRS at select time points. All datasets have the same number of samples (n=35).

Day	kurtosis	skewness	Bimodality coefficient
10	1.058234	0.855547	0.402
20	-0.4320937	0.3426146	0.396
28	-0.8857555	0.1518111	0.432
30	-0.9548306	0.1185866	0.441
40	-1.161462	0.001779072	0.478
50	-1.24207	-0.06602718	0.450
60	-1.266229	-0.108353	0.509
70	-1.263355	-0.1362011	0.512
80	-1.247162	-0.1552723	0.511
90	-1.224496	-0.1687531	0.507
100	-1.198934	-0.1785311	0.503

The section below contains the TRS SSDs and data visualization plots for each of the time points listed in Table 6. It is important to note that toxicity data for Classes Ostracoda and Maxillopoda were excluded from TRS toxicity set as there were insufficient toxicity data available in the literature to develop regression models for these Classes.

Day 10

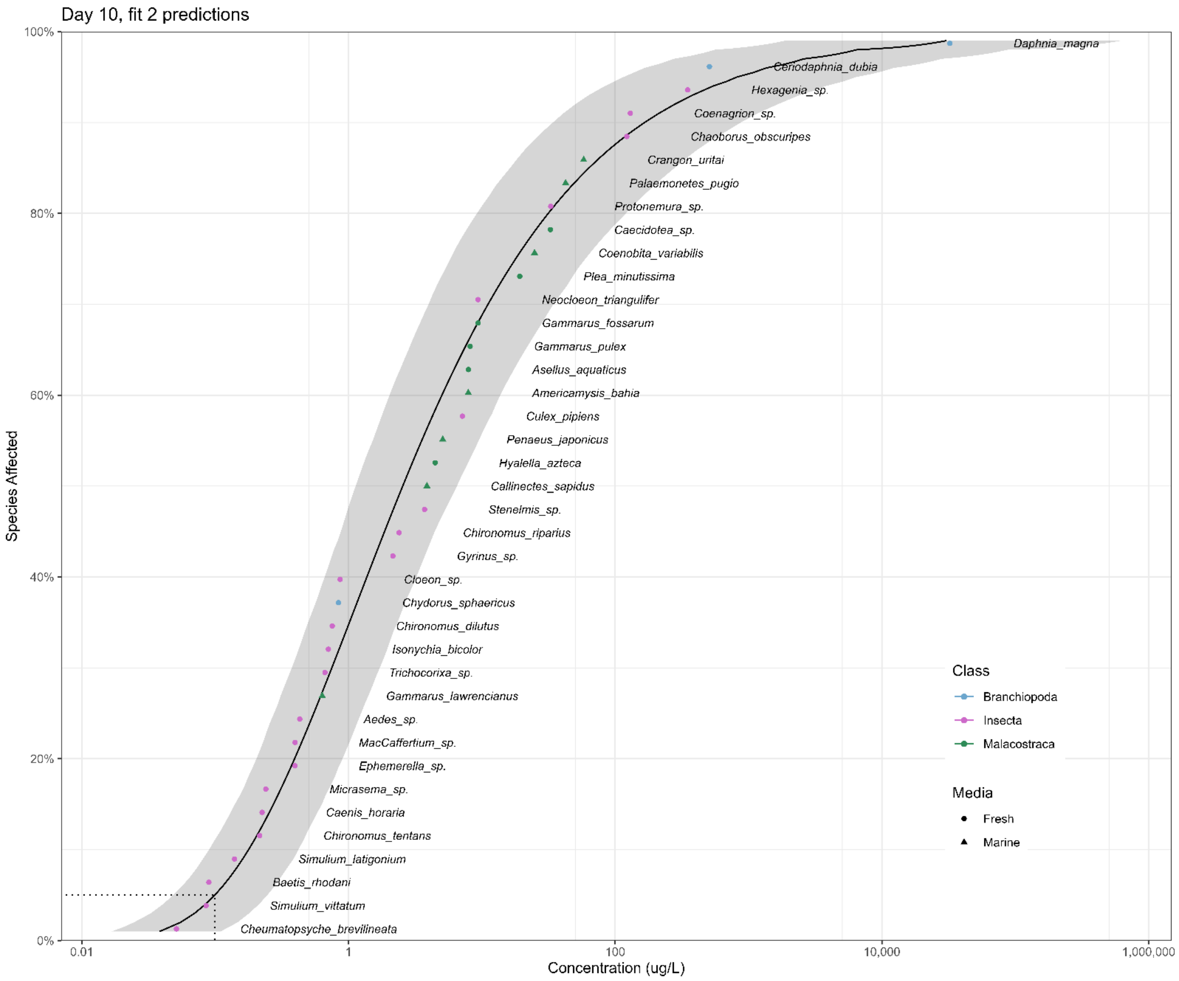

Figure 12 The species sensitivity distribution, generated with the R package ssdtools (73), for the imidacloprid TRS at day 10. The dotted line is the imidacloprid concentration that is protective of 95% of the aquatic ecosystem (PC95).

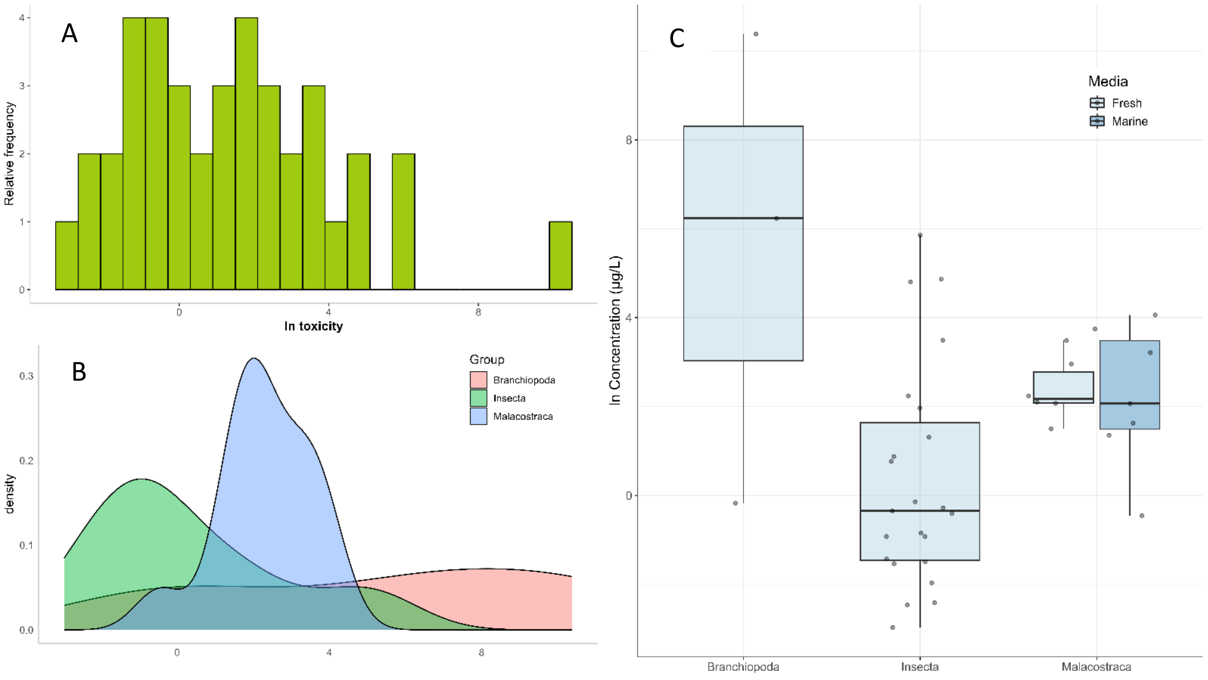

Figure 13 (A) a histogram showing the distribution of temporally adjusted toxicity data used to derive the SSD for day 10 of the TRS, alongside (B) a density plot and (C) box and whiskers plot that illustrates the relative sensitivity of each organism Class and media type to imidacloprid.

Day 20

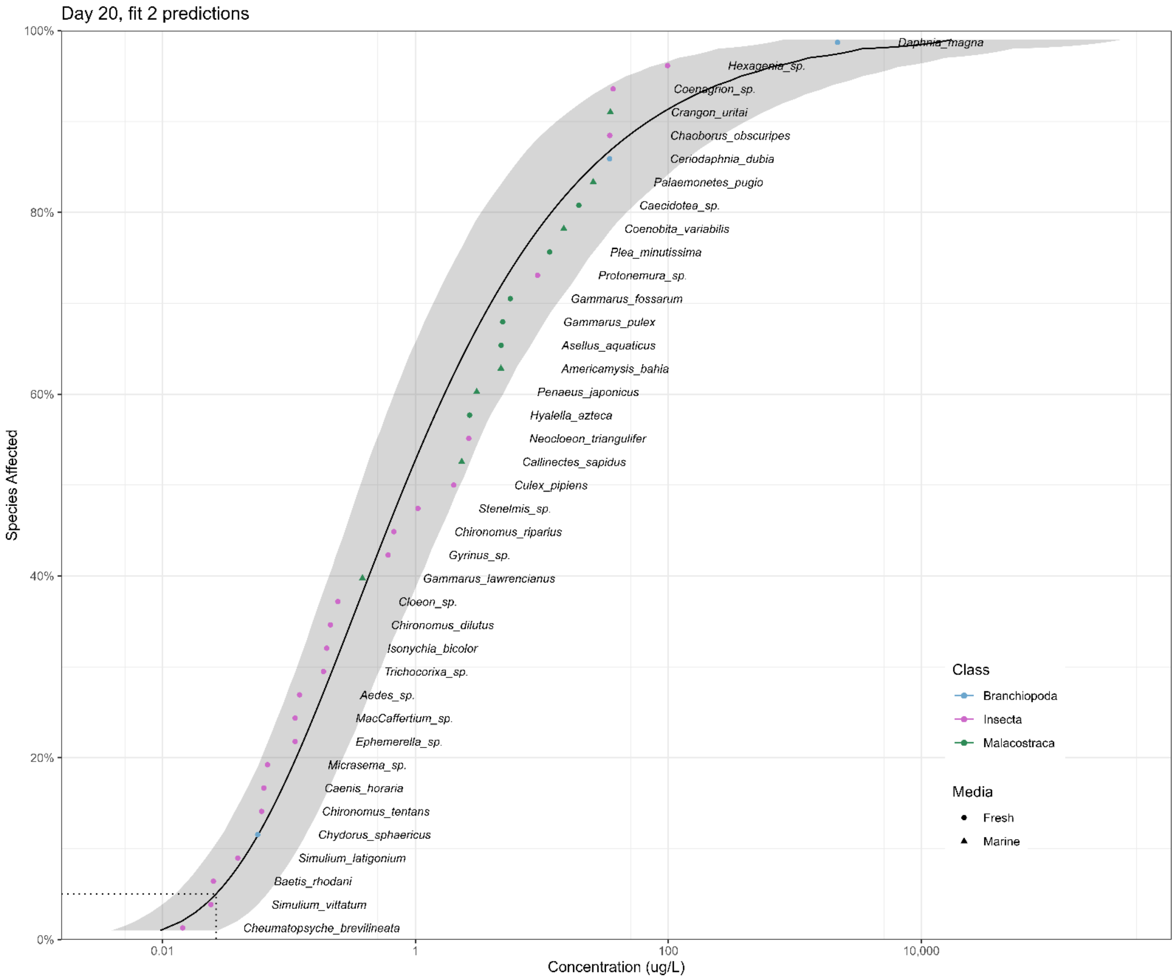

Figure 14 The species sensitivity distribution, generated with the R package ssdtools (73), for the imidacloprid TRS at day 20. The dotted line is the imidacloprid concentration that is protective of 95% of the aquatic ecosystem (PC95).

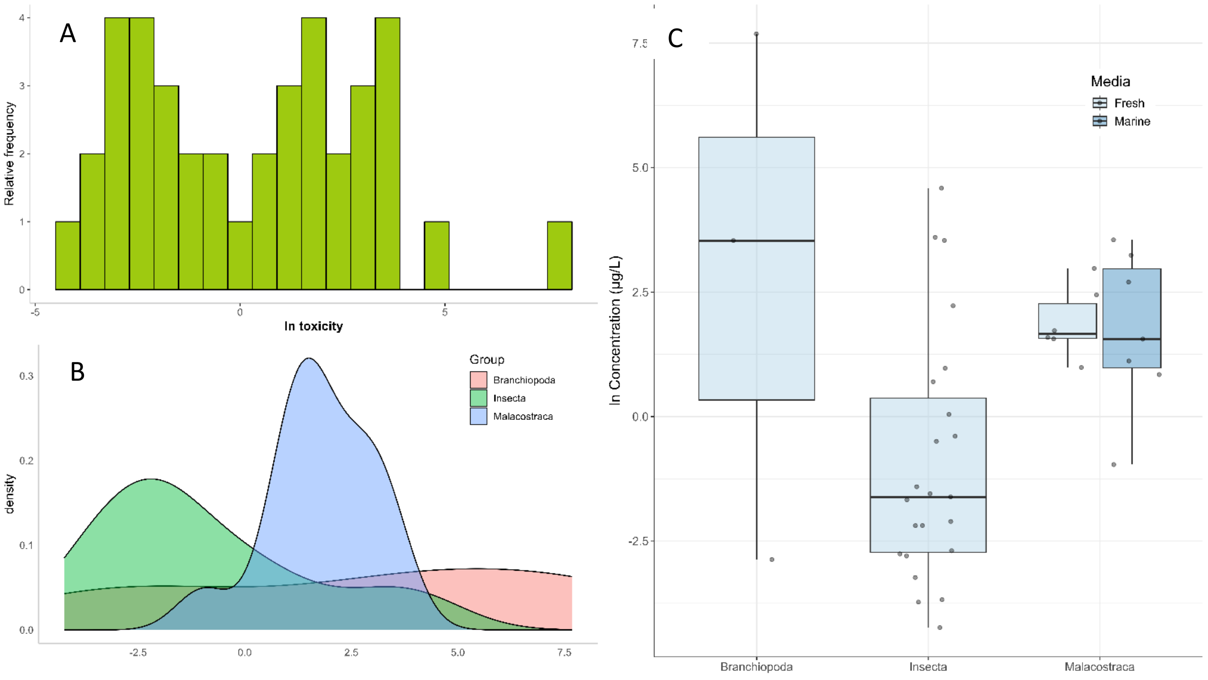

Figure 15 (A) a histogram showing the distribution of temporally adjusted toxicity data used to derive the SSD for day 20 of the TRS, alongside (B) a density plot and (C) box and whiskers plot that illustrates the relative sensitivity of each organism Class and media type to imidacloprid.

Day 28

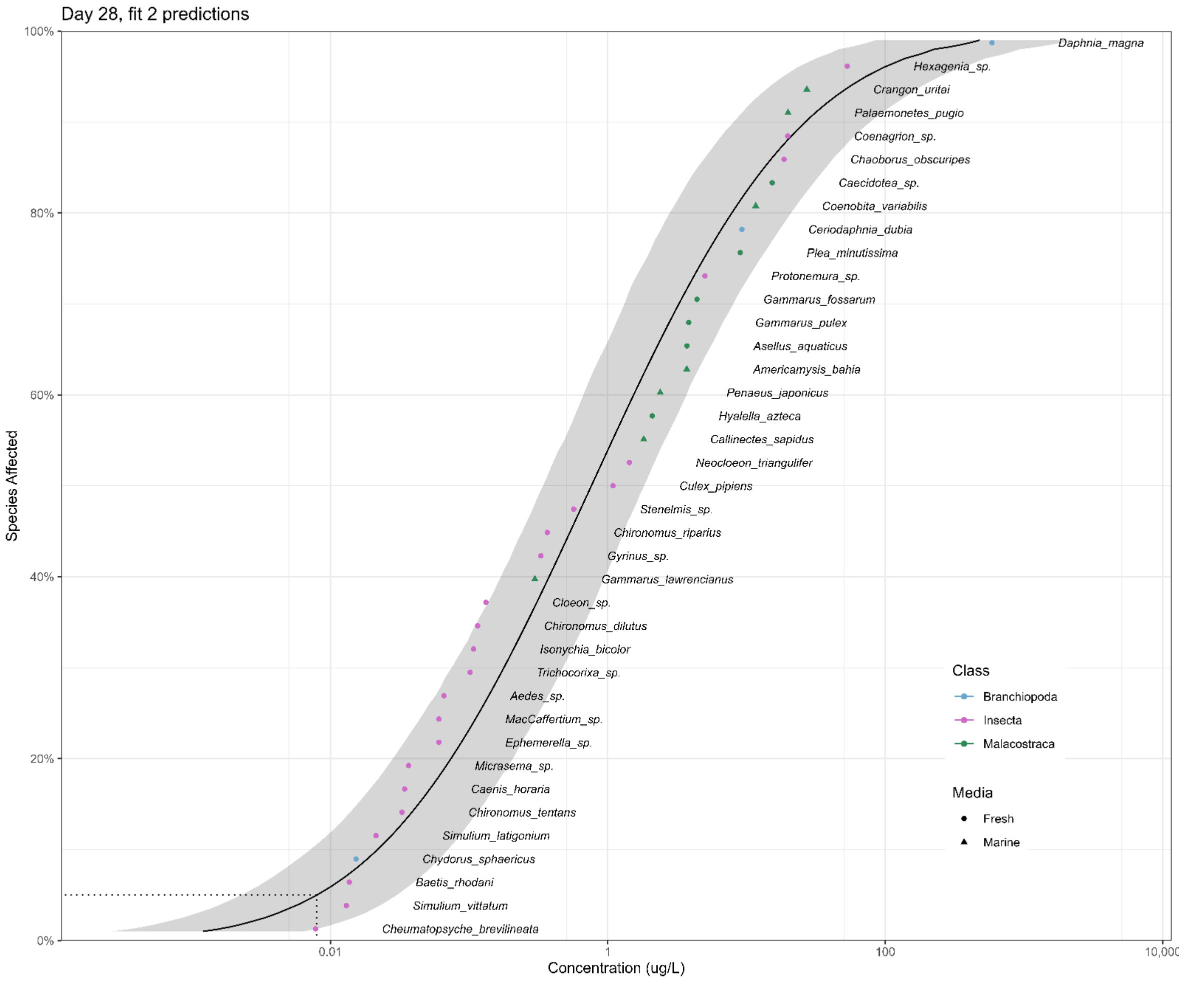

Figure 16 The species sensitivity distribution, generated with the R package ssdtools (73), for the imidacloprid TRS at day 20. The dotted line is the imidacloprid concentration that is protective of 95% of the aquatic ecosystem (PC95).

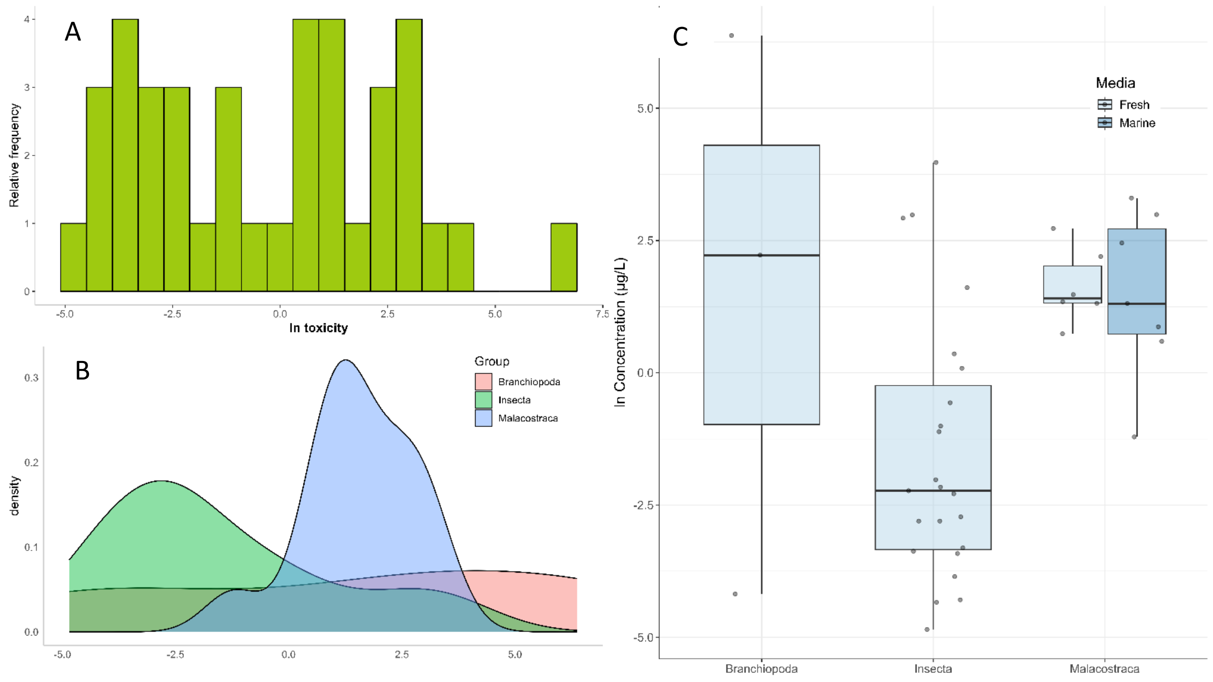

Figure 17 (A) a histogram showing the distribution of temporally adjusted toxicity data used to derive the SSD for day 20 of the TRS, alongside (B) a density plot and (C) box and whiskers plot that illustrates the relative sensitivity of each organism Class and media type to imidacloprid.

Day 30

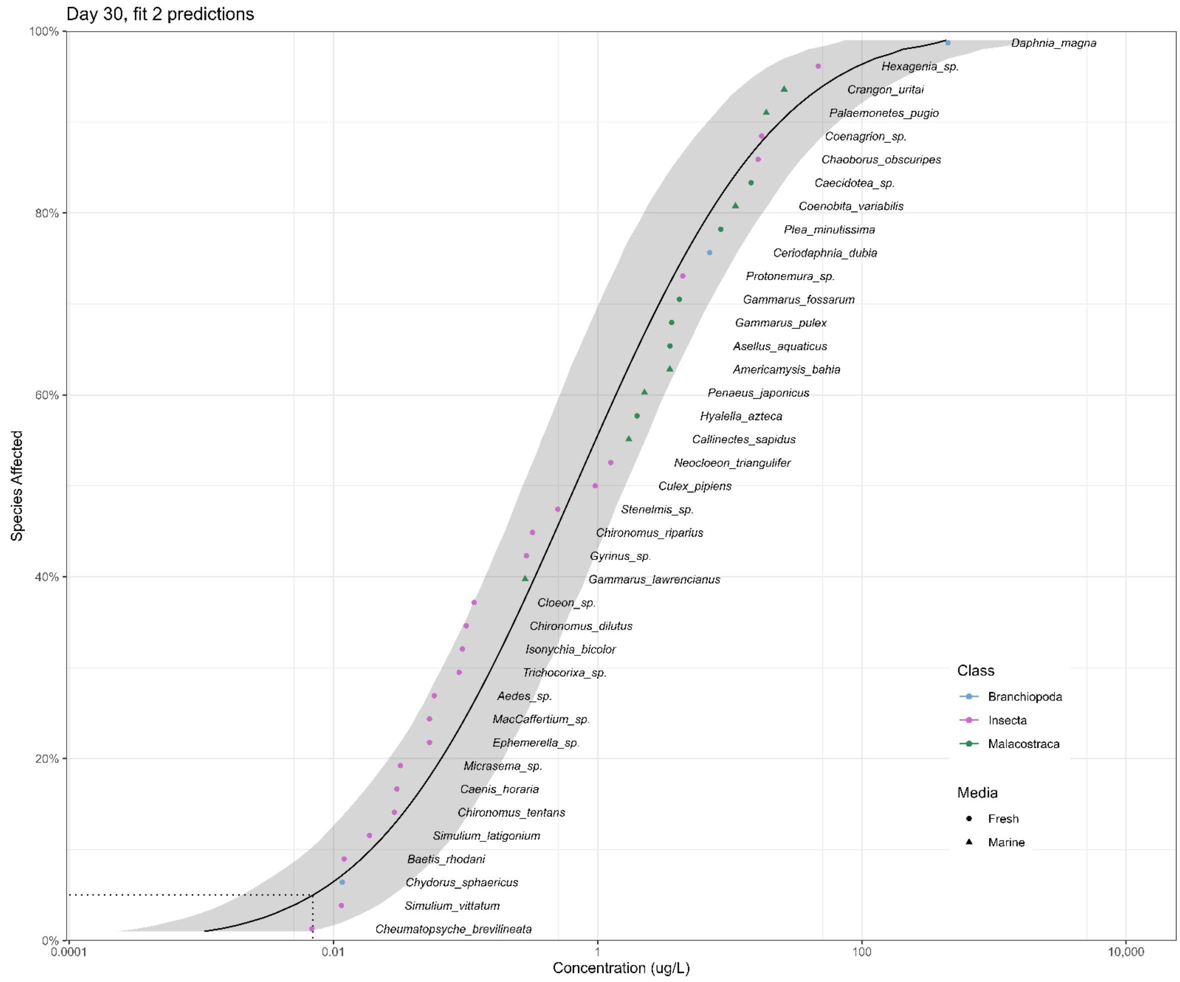

Figure 18 The species sensitivity distribution, generated with the R package ssdtools (73), for the imidacloprid TRS at day 30. The dotted line is the imidacloprid concentration that is protective of 95% of the aquatic ecosystem (PC95).

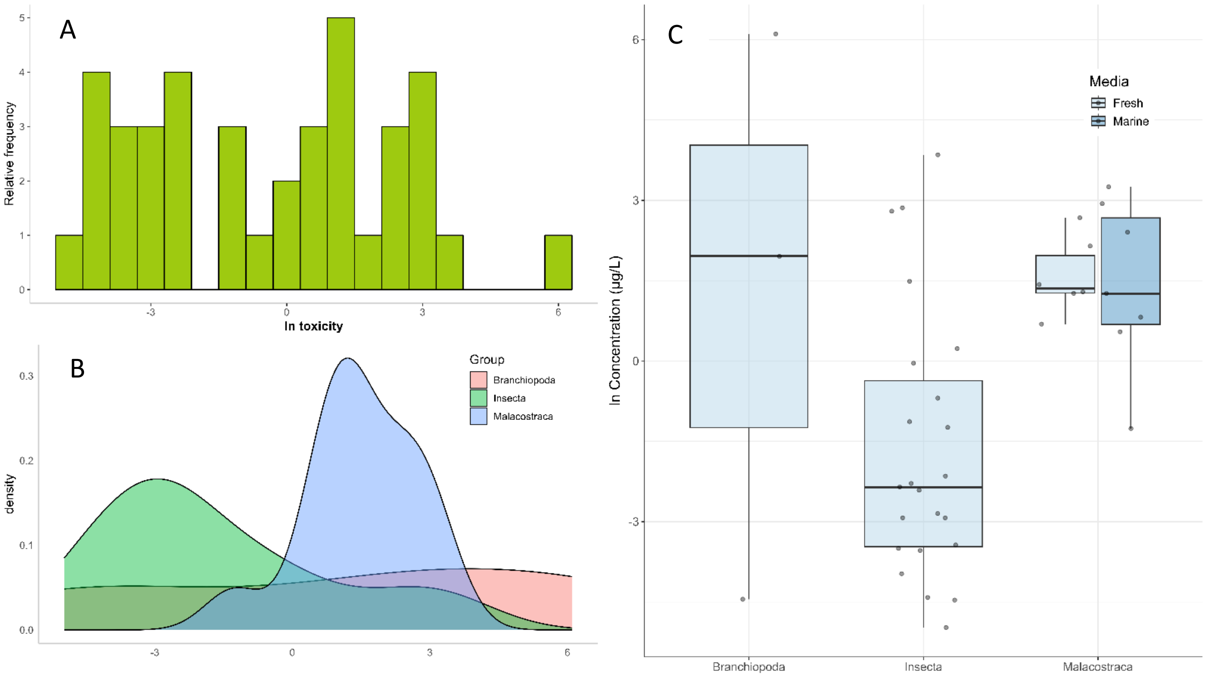

Figure 19 (A) a histogram showing the distribution of temporally adjusted toxicity data used to derive the SSD for day 30 of the TRS, alongside (B) a density plot and (C) box and whiskers plot that illustrates the relative sensitivity of each organism Class and media type to imidacloprid.

Day 40

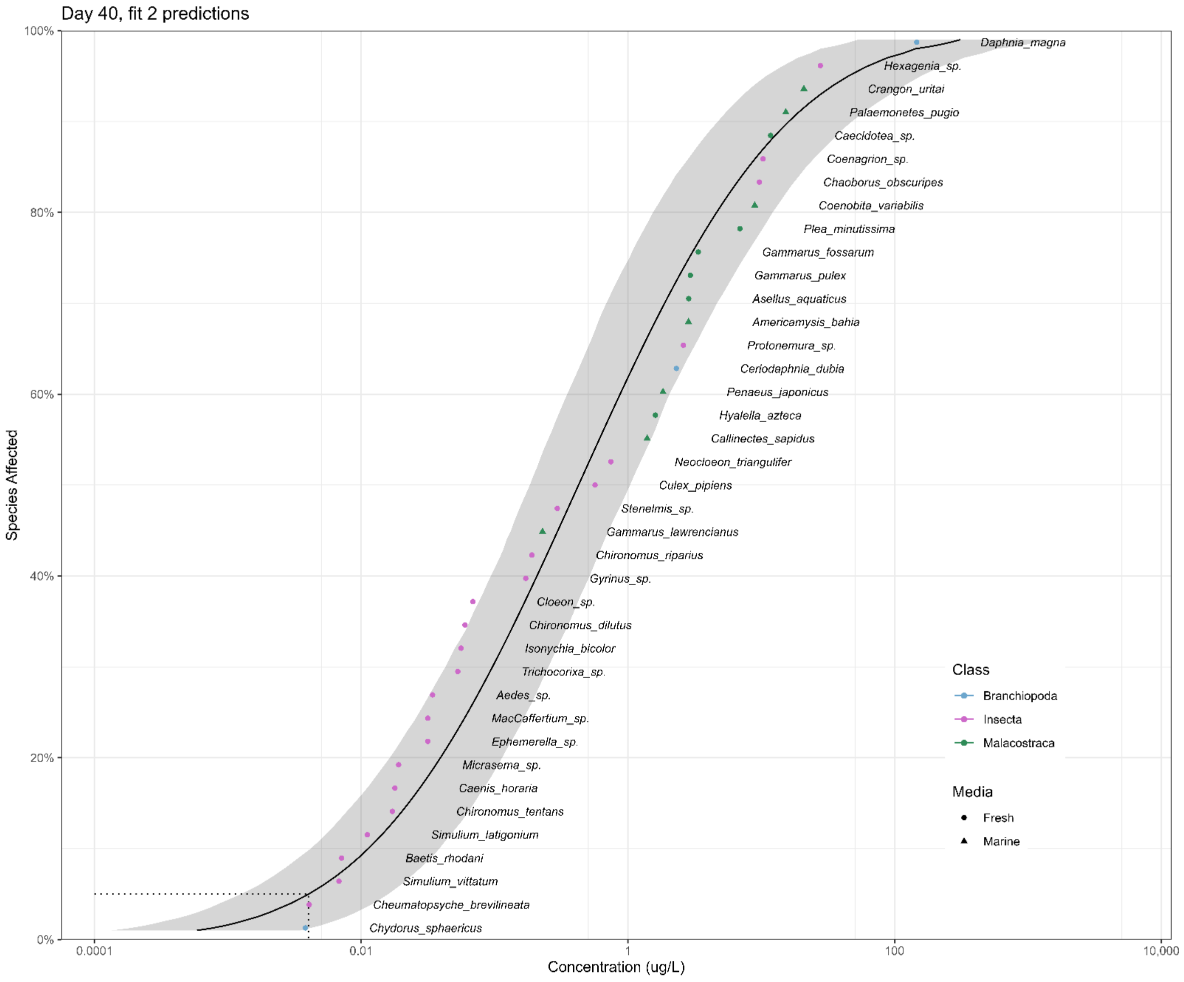

Figure 20 The species sensitivity distribution, generated with the R package ssdtools (73), for the imidacloprid TRS at day 40. The dotted line is the imidacloprid concentration that is protective of 95% of the aquatic ecosystem (PC95).

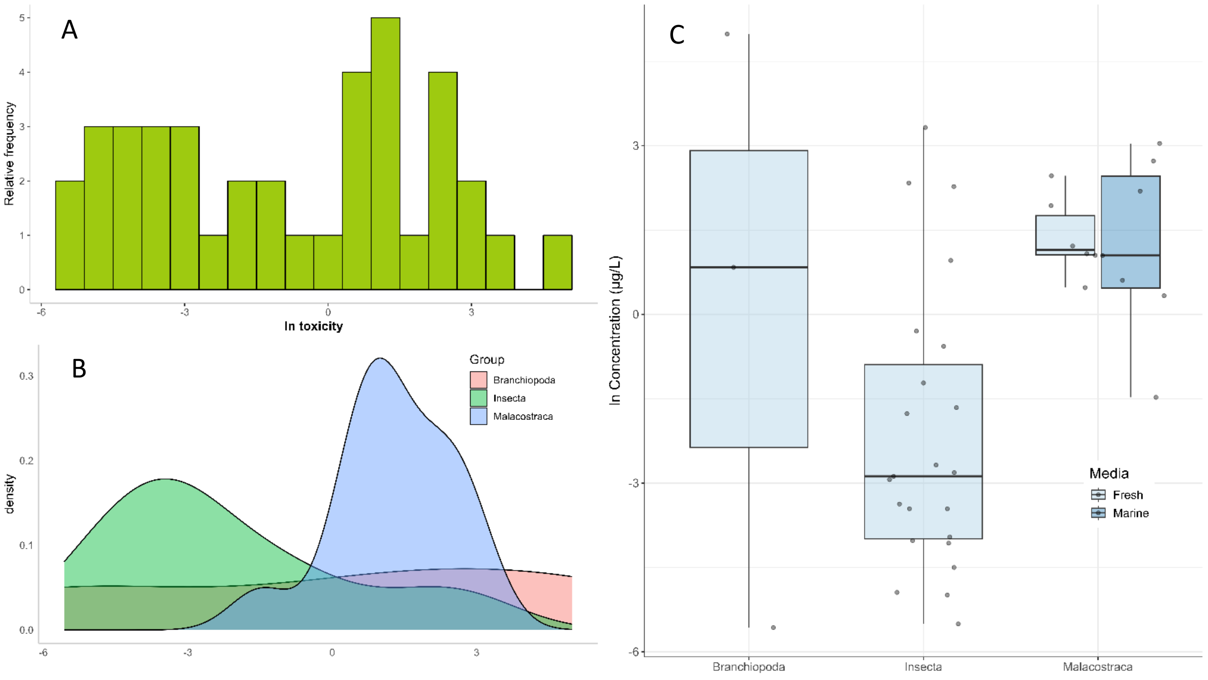

Figure 21 (A) a histogram showing the distribution of temporally adjusted toxicity data used to derive the SSD for day 40 of the TRS, alongside (B) a density plot and (C) box and whiskers plot that illustrates the relative sensitivity of each organism Class and media type to imidacloprid.

Day 50

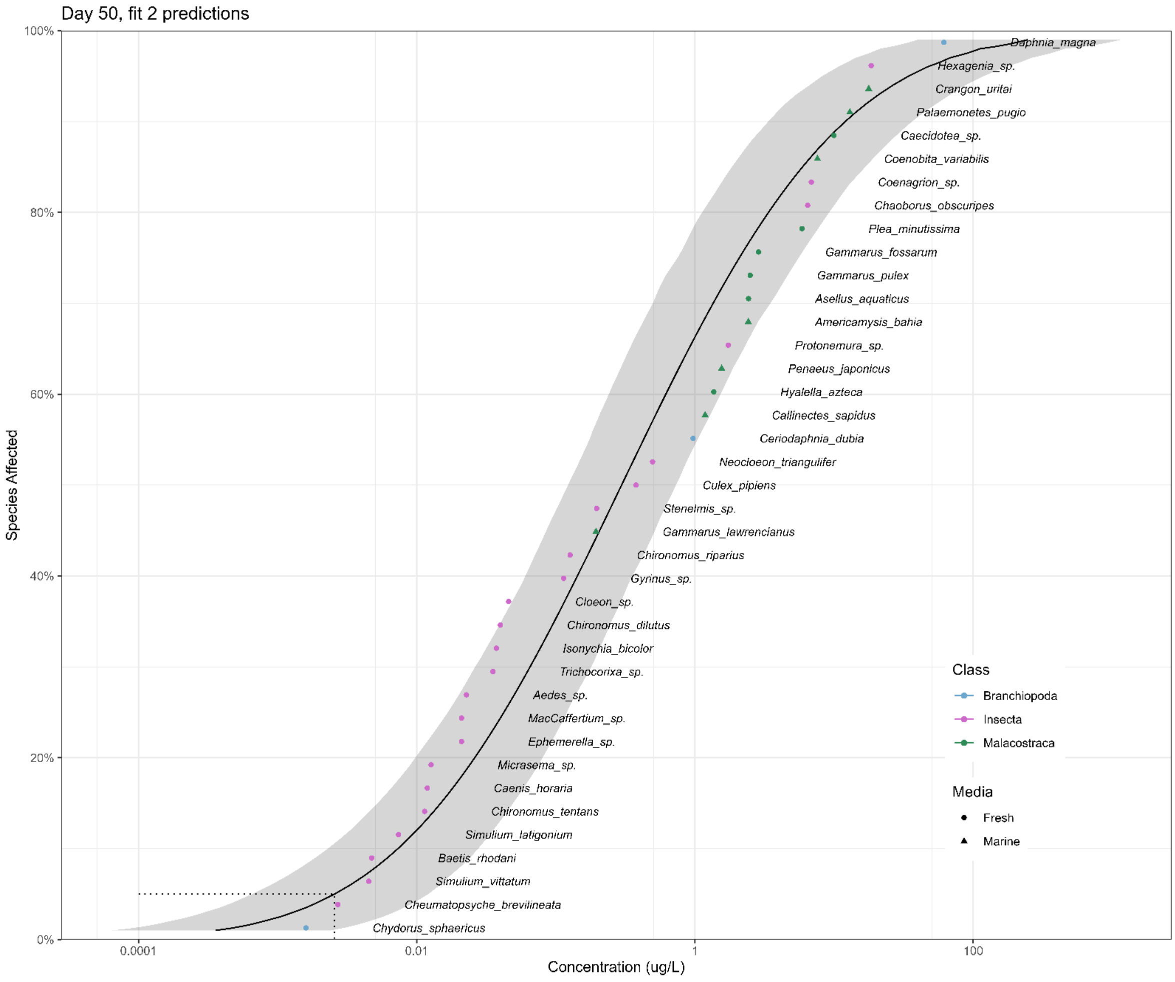

Figure 22 The species sensitivity distribution, generated with the R package ssdtools (73), for the imidacloprid TRS at day 50. The dotted line is the imidacloprid concentration that is protective of 95% of the aquatic ecosystem (PC95).

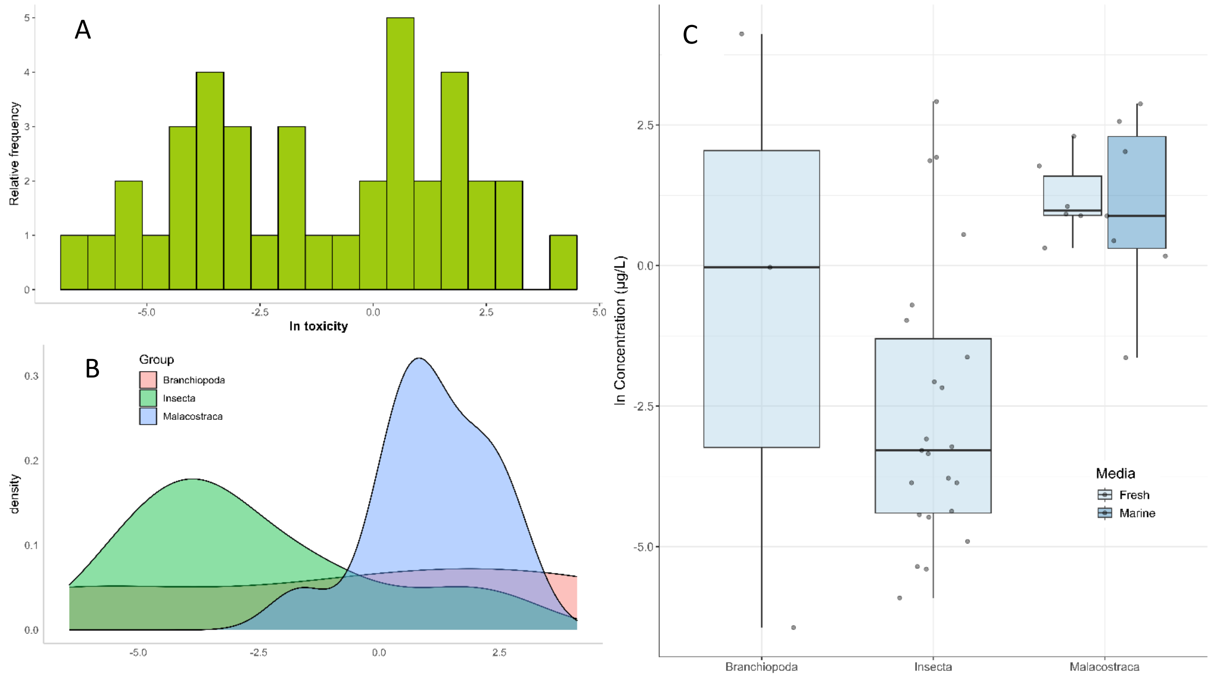

Figure 23 (A) a histogram showing the distribution of temporally adjusted toxicity data used to derive the SSD for day 50 of the TRS, alongside (B) a density plot and (C) box and whiskers plot that illustrates the relative sensitivity of each organism Class and media type to imidacloprid.

Day 60

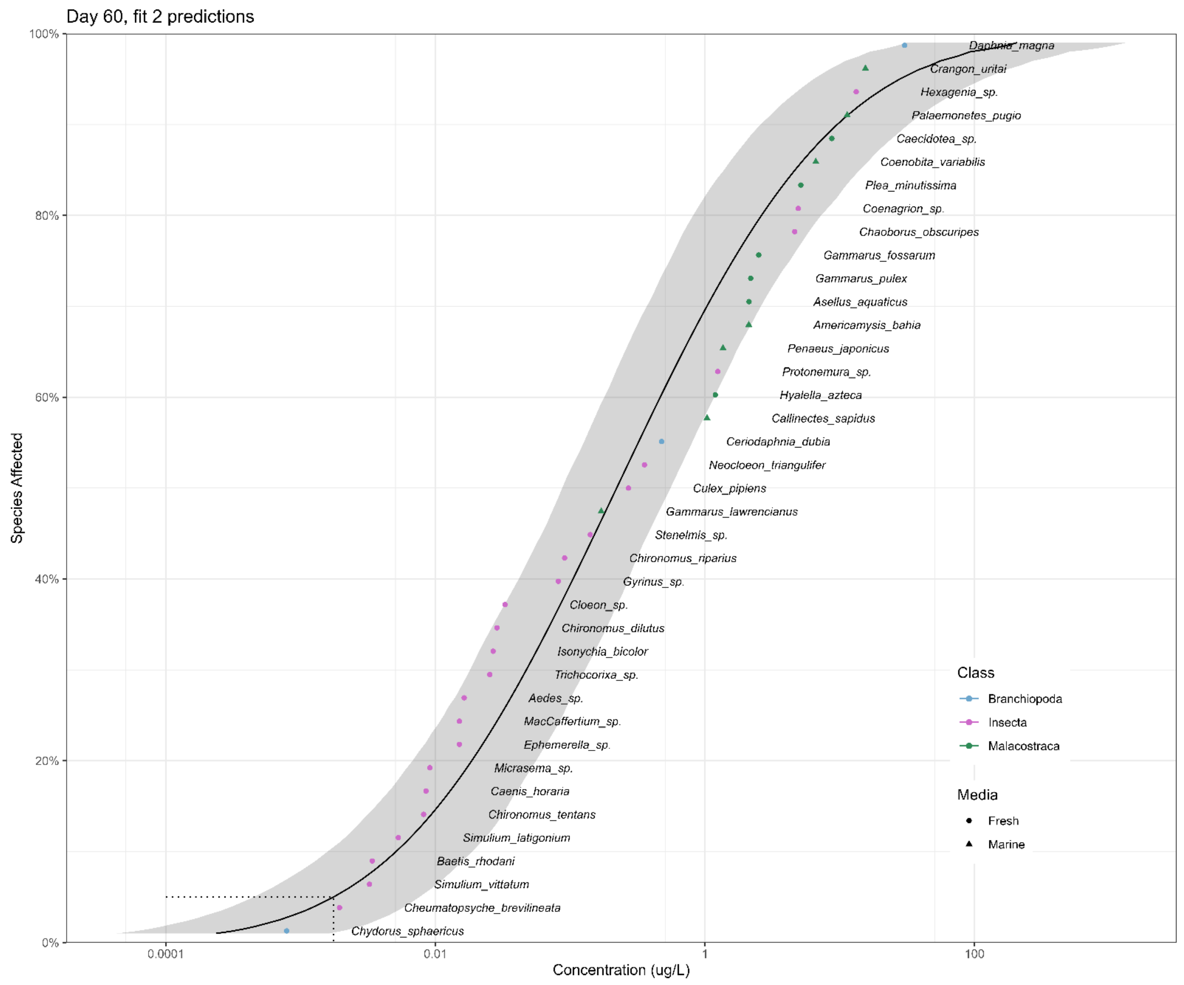

Figure 24 The species sensitivity distribution, generated with the R package ssdtools (73), for the imidacloprid TRS at day 60. The dotted line is the imidacloprid concentration that is protective of 95% of the aquatic ecosystem (PC95).

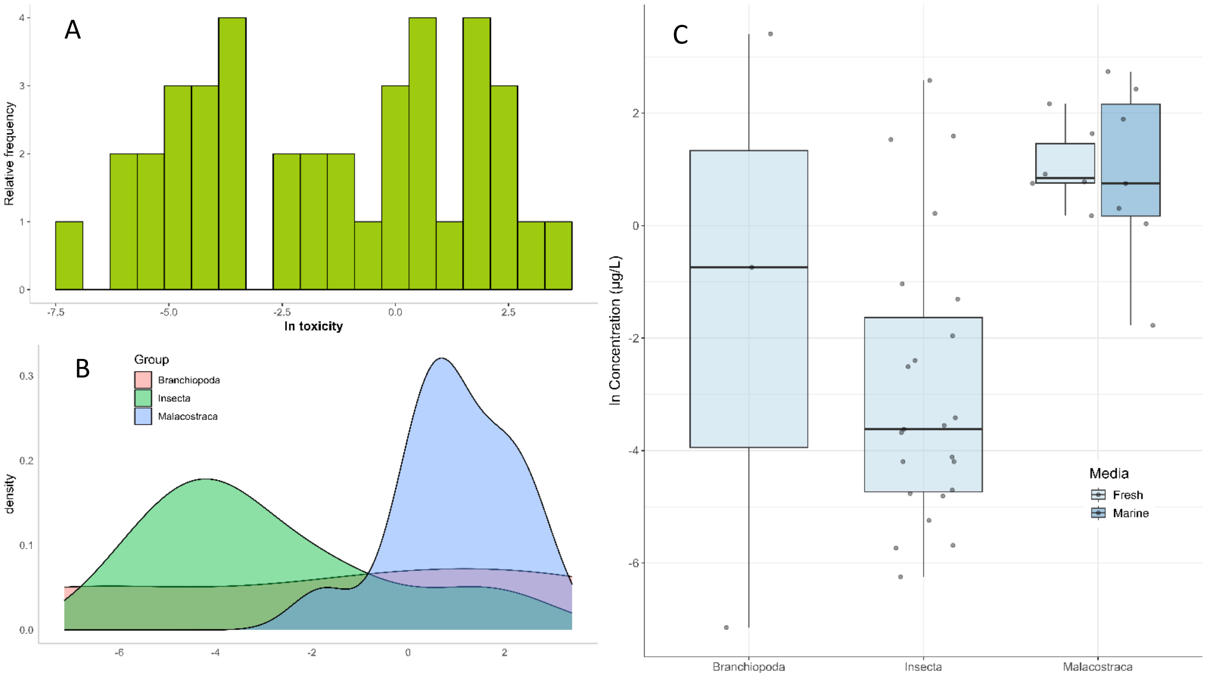

Figure 25 (A) a histogram showing the distribution of temporally adjusted toxicity data used to derive the SSD for day 60 of the TRS, alongside (B) a density plot and (C) box and whiskers plot that illustrates the relative sensitivity of each organism Class and media type to imidacloprid.

Day 70

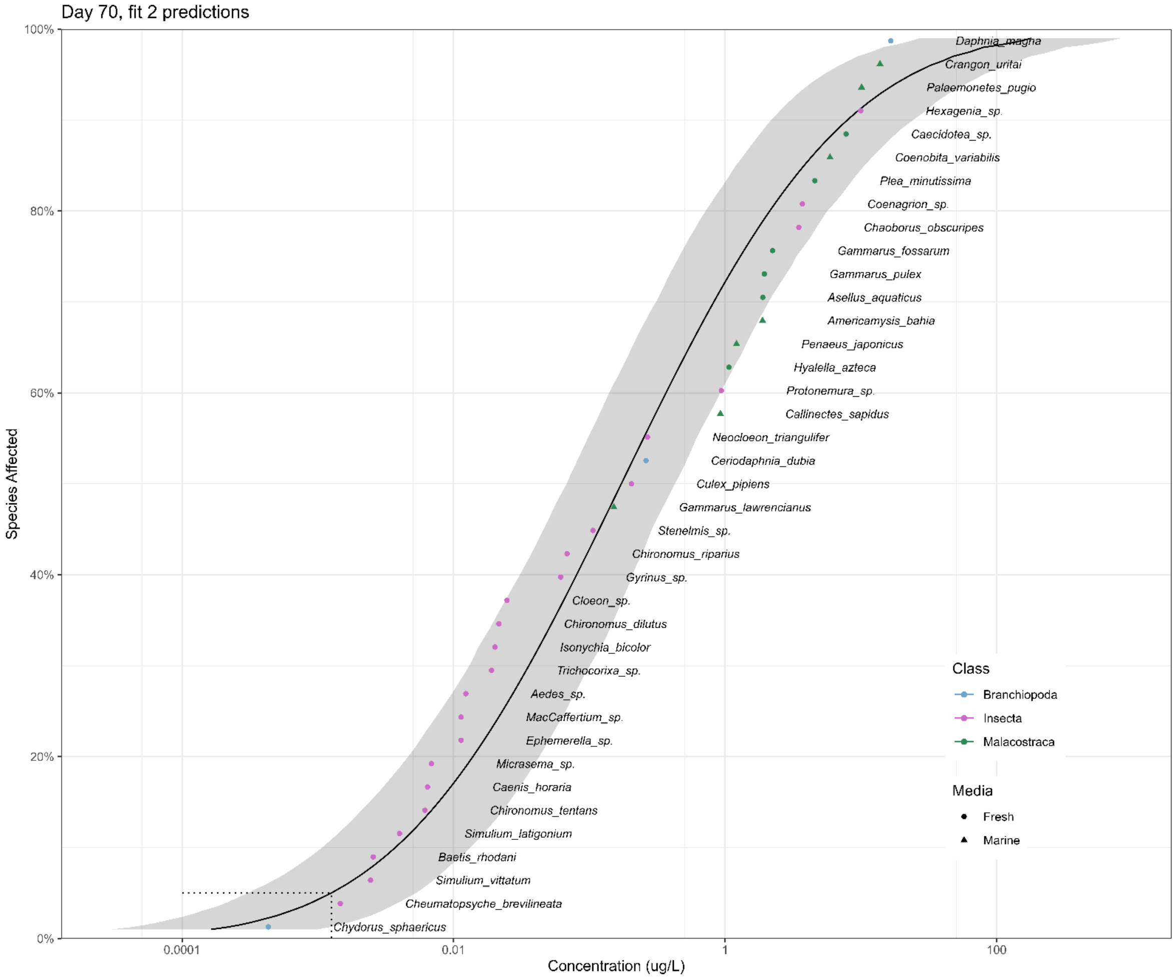

Figure 26 The species sensitivity distribution, generated with the R package ssdtools (73), for the imidacloprid TRS at day 70. The dotted line is the imidacloprid concentration that is protective of 95% of the aquatic ecosystem (PC95).

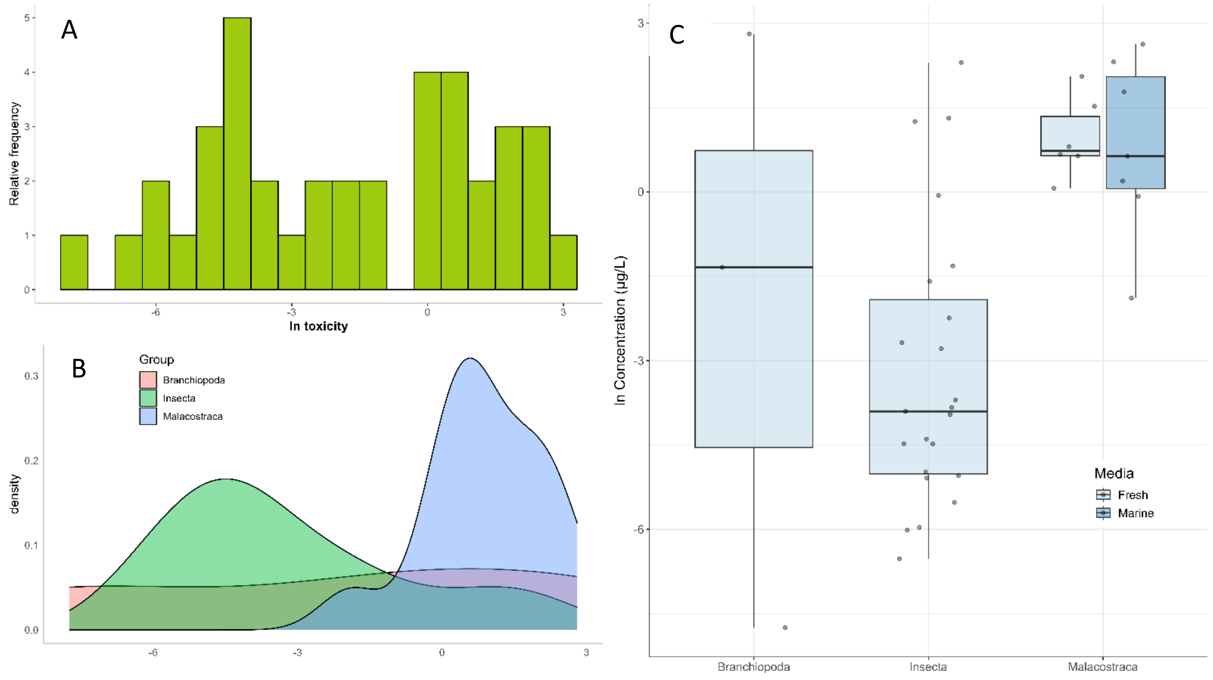

Figure 27 (A) a histogram showing the distribution of temporally adjusted toxicity data used to derive the SSD for day 70 of the TRS, alongside (B) a density plot and (C) box and whiskers plot that illustrates the relative sensitivity of each organism Class and media type to imidacloprid.

Day 80

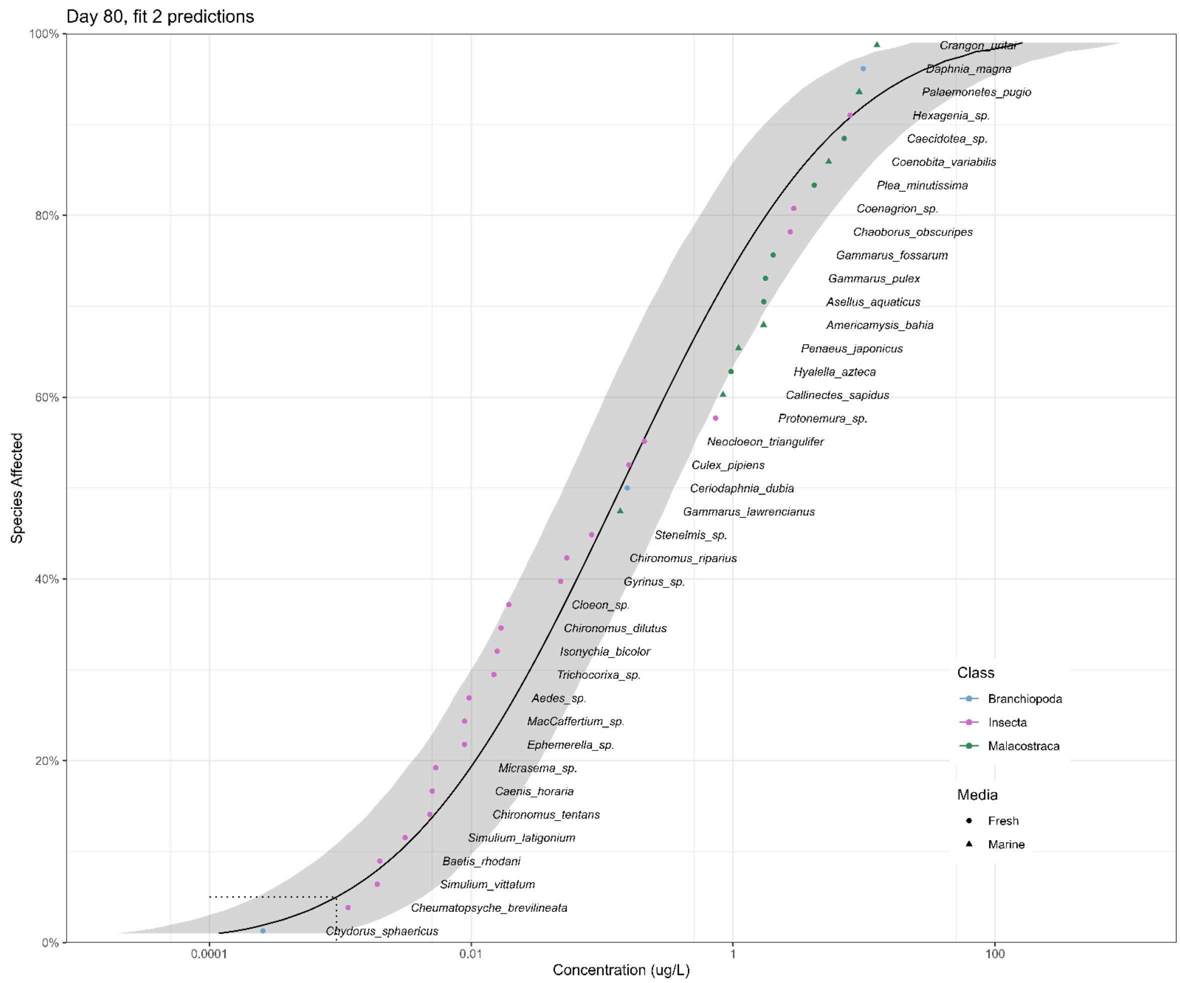

Figure 28 The species sensitivity distribution, generated with the R package ssdtools (73), for the imidacloprid TRS at day 80. The dotted line is the imidacloprid concentration that is protective of 95% of the aquatic ecosystem (PC95).

A

C

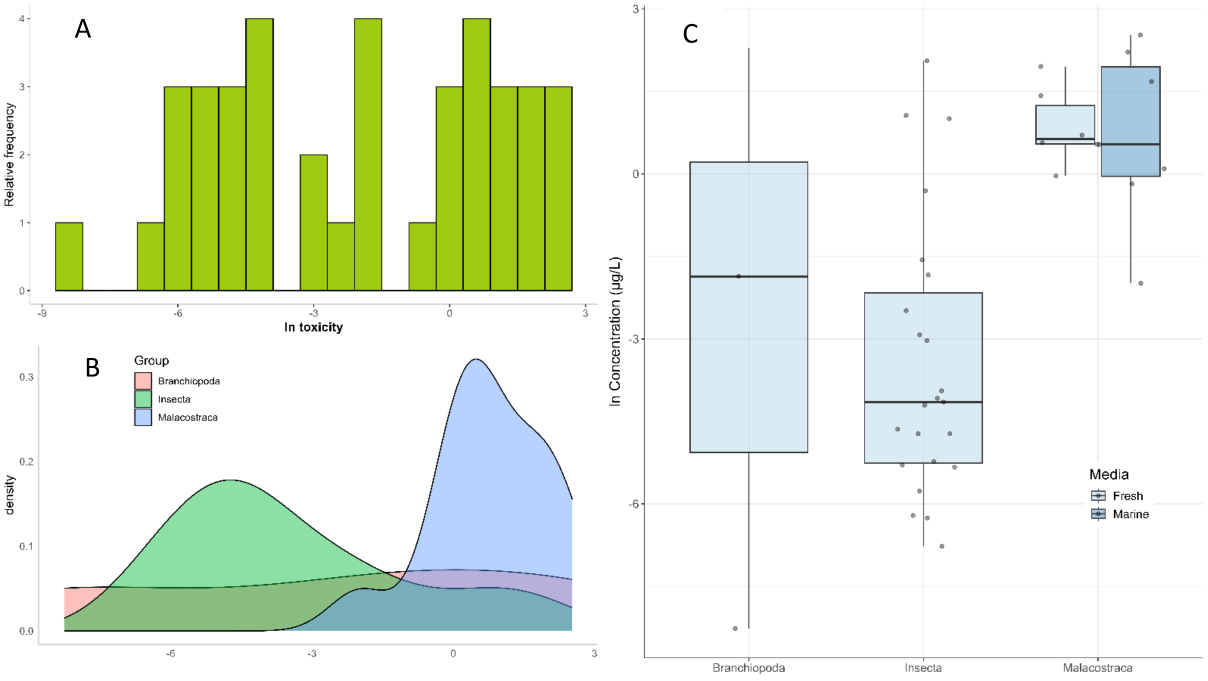

Figure 29 (A) a histogram showing the distribution of temporally adjusted toxicity data used to derive the SSD for day 80 of the TRS, alongside (B) a density plot and (C) box and whiskers plot that illustrates the relative sensitivity of each organism Class and media type to imidacloprid.

Day 90

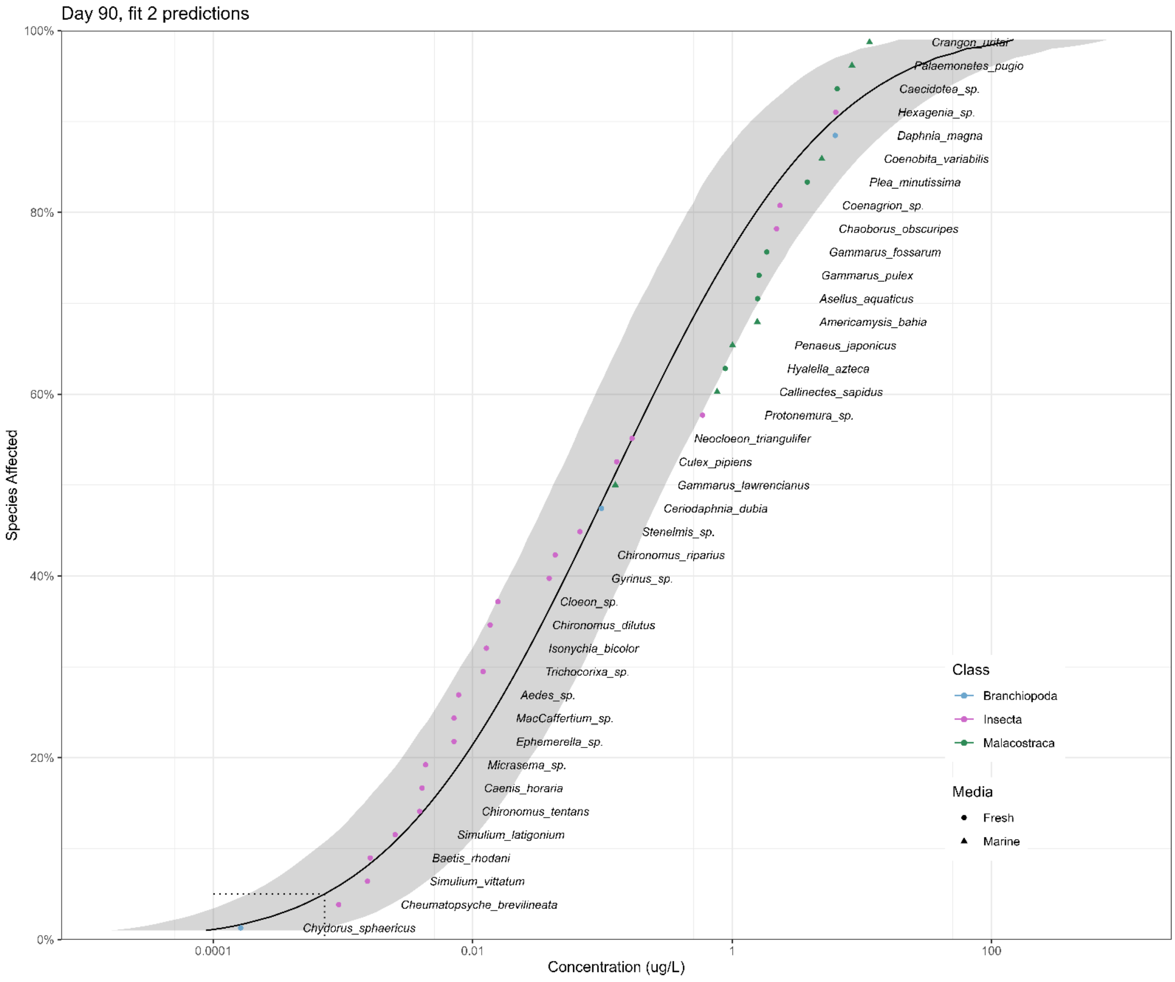

Figure 30 The species sensitivity distribution, generated with the R package ssdtools (73), for the imidacloprid TRS at day 90. The dotted line is the imidacloprid concentration that is protective of 95% of the aquatic ecosystem (PC95).

A

C

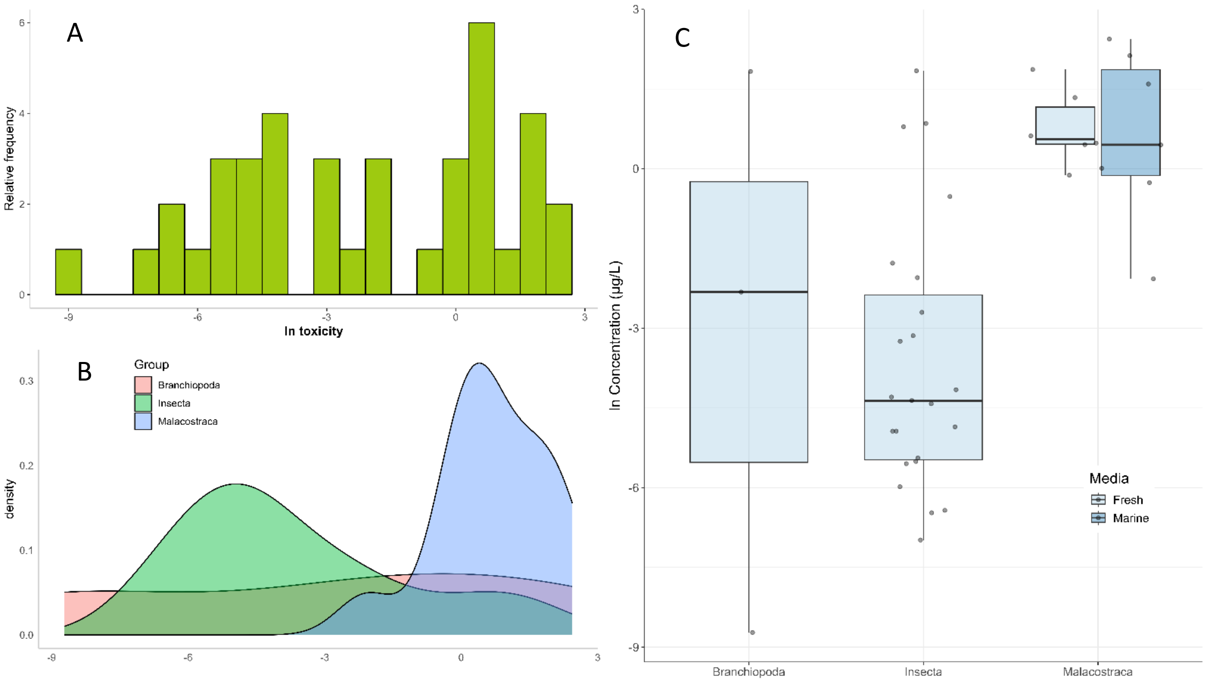

Figure 31 (A) a histogram showing the distribution of temporally adjusted toxicity data used to derive the SSD for day 90 of the TRS, alongside (B) a density plot and (C) box and whiskers plot that illustrates the relative sensitivity of each organism Class and media type to imidacloprid.

Day 100

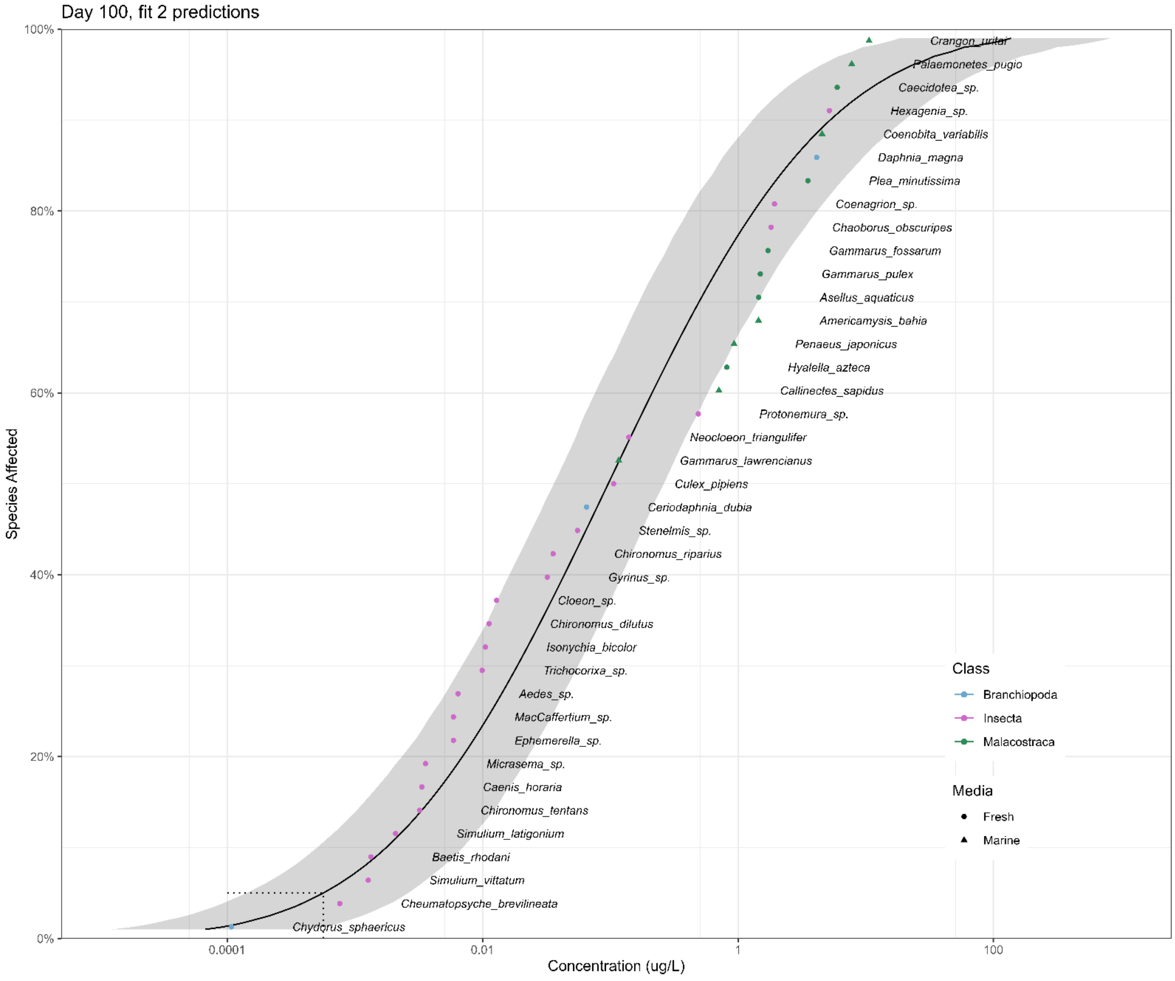

Figure 32 The species sensitivity distribution, generated with the R package ssdtools (73), for the imidacloprid TRS at day 100. The dotted line is the imidacloprid concentration that is protective of 95% of the aquatic ecosystem (PC95).

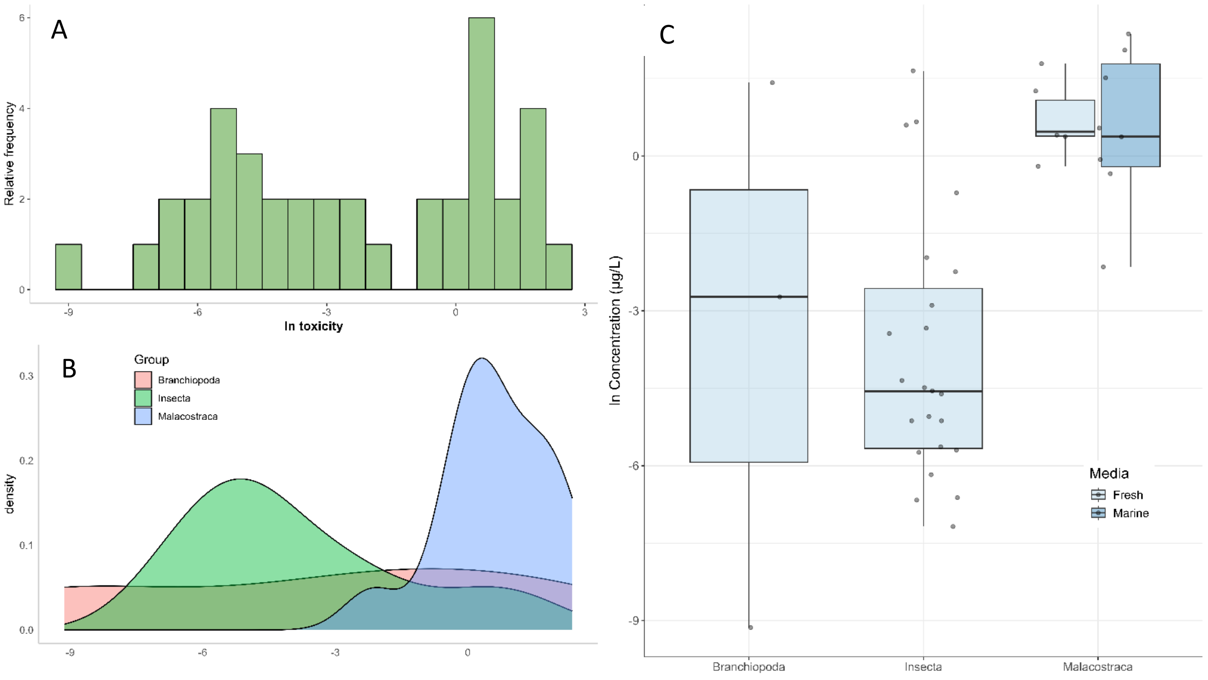

Figure 33 (A) a histogram showing the distribution of temporally adjusted toxicity data used to derive the SSD for day 100 of the TRS, alongside (B) a density plot and (C) box and whiskers plot that illustrates the relative sensitivity of each organism Class and media type to imidacloprid.

S10: Ecosystem impacts of imidacloprid exposure: literature review and TRS analysis

This SI section contains a literature review that investigates the link between long-term imidacloprid exposure and actual ecosystem outcomes. The findings allow for further validation of the imidacloprid Temporal Response Surface (TRS) method and illustration of its use within a probabilistic risk assessment framework. The review is organised into the following sections:

1. Evidence of cumulative effects at the organism level from pulsed exposure to imidacloprid. This is further separated into toxicokinetic/toxicodynamic (TK/TD) studies, and non-TK/TD studies;
2. Evidence of delayed recovery of organisms and ecosystems resulting from exposure to imidacloprid; and
3. A comparison of TRS estimates of risk with the findings of micro- and mesocosm studies.

Points 1 and 2 above are included to provide additional evidence of the cumulative toxicity and delayed recovery of imidacloprid, further justifying the TRS approach which takes these factors into account. Where possible, toxicity estimates and effects are compared with those of the TRS to validate the model. Section 3 (meso- and microcosm studies) is included to assess whether significant ecosystem-level effects are observed at concentrations and exposure durations that are likely to occur in the Great Barrier Reef catchment area. Where these effects are the result of constant (not pulsed) exposure, an assessment is made whether the TRS PC values provide sufficient protection under the same exposure conditions. Literature on other neonicotinoids are occasionally discussed under the assumption that the study outcomes are relevant to imidacloprid due similar modes of action and time-cumulative-toxicity characteristics.

Evidence of cumulative effects

Toxicokinetic/toxicodynamic (TK/TD) studies

Toxicokinetic/toxicodynamic (TK/TD) studies combine experimental bioassay data with computational models to understand the absorption, distribution, metabolism, and excretion of toxic substances at the organism level. The models are generally complex and species-specific. Once calibrated, they provide a detailed understanding of how toxic substances behave in the body and the resulting toxic effects, enabling more detailed and accurate risk assessment (51, 74). They can be used to test the effects of a wide range of concentration and exposure scenarios, including pulsed

and chronic exposure. They can also be used to understand the internal metabolic processes driving the toxicity patterns observed in bioassay, meso-, and microcosm studies. In this way, TK/TD models have a purpose similar to the TRS in terms of enabling risk assessment under conditions that would otherwise be virtually impossible to test for (51). This literature review focusses on the use of TK/TD models to further validate the toxicity estimates of the TRS at the species level

In terms of validating the findings of the current study, a study by Li, Zhang, et al. (37) found that, while *Daphnia magna* is tolerant to short exposures of imidacloprid (96-h LC50 of 8.5 mg/L), pulsed exposure led to significant delayed and carry-over toxicity as a result of slow damage recovery (~45 days). This finding emphasizes the importance of incorporating delayed and carry-over toxicity measurements when evaluating the risks posed by neonicotinoids to aquatic invertebrates, even for tolerant species like *D. magna*. Their findings validate the increase in toxicity observed in *D. magna* in the current study, where the predicted LC50 fell to 1.01 µg/L following 100 days of continuous exposure (see Figure 2B of manuscript). Mangold-Döring, Buddendorf, et al. (43) conducted a TK/TD study testing the effects of prolonged imidacloprid exposure on the amphipod *Gammarus pulex*. They found that constant exposure to 0.4 µg/L resulted in the entire population of *G. pulex* becoming extinct part way through the 10-year modelled exposure period. The *G. pulex* bioassay datum used in the current study was 8.02 µg/L which is a geomean of two 96-hour EC50 immobilisation data points (6, 78). When adjusted using the Malacostraca regression model, the predicted EC50 became 1.50 µg/L following 100 days of continuous exposure. The Mangold-Döring, Buddendorf, et al. (43) study modelled complete extinction (i.e. LC100) and the current study predicted 50% immobilisation of the test population (i.e. EC50), so the difference in concentration values seems reasonable. Huang, van den Brink, et al. (28) used a combination of acute bioassay tests and TK/TD modelling to investigate the time-cumulative-toxicity effects of imidacloprid and a metabolite (IMI-ole) on the mayfly *Cleon dipterum* and the amphipod *Gammarus pulex*. They found that much of the delayed toxicity of imidacloprid could be attributed to the biotransformation of imidacloprid to IMI-ole, which was as toxic as the parent compound but poorly eliminated by both organisms. *C.dipterum* was more sensitive than *G.pulex* due to a combination of faster metabolism of the parent compound and slower elimination of the metabolite. However, IMI-ole was persistent within the body tissue of both species. The authors conclude that there may be no reasonable long-term threshold for *C.dipterum* and other similarly sensitive organisms, as poor elimination results in an ongoing increase in toxicity over time. Mayflies appear to be particularly sensitive, with chronic NOEC values from other studies in the ng/L range (69). This ongoing increase in toxicity was evident for the mayflies included in the TRS, even *Hexagenia sp.* which is relatively insensitive compared to the other species (**Error! Reference source not found.**). When adjusted to 100 days of continuous exposure using the Insecta regression model the effect concentration for all mayfly species dropped by approximately 3 orders of magnitude.

Othe cumulative studies

Hayasaka, Korenaga, et al. (25) applied imidacloprid and fipronil to a rice paddy ecosystem through two pulsed exposures one year apart, via treated rice seedlings. While soil concentrations increased between the two years, the aqueous concentrations remained relatively constant in both years. However, impacts were far greater for water surface-dwelling and water-borne arthropods in the second year. There was no recovery of the zooplankton composition and structure in both insecticide-treated mesocosms throughout the second experimental period. Likewise, Hayasaka, Kobashi, et al. (23) found that changes in community composition and structure of aquatic insects were much greater in the second year of imidacloprid application in a similar study of rice paddies in Japan. This pattern was particularly evident for species with long life cycles, as these were likely exposed to persistent residues of imidacloprid in the soil that became soluble over time. In sub-tropical freshwater microcosms in Bangladesh, Sumon, Ritika, et al. (72) repeated applied weekly pulses of imidacloprid at three different treatment levels, and all of them severely impacted severely the aquatic community, particularly mayflies (*Cloeon* sp.), copepods *(Diaptomus* sp.) and rotifers (*Keratella* sp.).

Evidence of delayed recovery

In a study by Alexander, Culp, et al. (3), mayflies and oligochaetes were subjected to a 24-hour pulsed exposure of imidacloprid, which resulted in significant suppression of feeding rate that lasted for at least four days post-exposure. These effects occurred at concentrations known to occur in rivers and creeks in Australia (80) (0.1 µg/L for the mayfly *Epeorus longimanus*, and 0.5 µg/L for the Oligochaete *Lumbriculus variegatus*). Other studies show that while some

aquatic organisms do not die immediately with a single pulse imidacloprid exposure, mortality can occur for extended periods after the organisms are transferred to clean water (37). For some, their populations disappear completely after a few weeks and do not recover unless there is external recruitment into the system (23, 38, 66). This delayed effect may in part be due to the gradual metabolic transformation of imidacloprid to toxic metabolites such as imidacloprid-olefin and hydroxy-imidacloprid that induce mortality (4, 27, 78). Beketov, Schäfer, et al. (10) found that recovery time was dependant on the life-cycle characteristics of individual species, not toxicant concentration. Short-lived (multivoltine) species recovered after 10 weeks following thiacloprid contamination, whereas long-living (uni- and semivoltine) species did not recover until the end of the experiment (7 months). They conclude that realistic prediction of community recovery dynamics requires consideration of the life-cycle traits of individual species. Delayed recovery is generally not considered or captured in current regulatory approaches that are based on standardised bioassay approaches (37).

Comparison with micro- and mesocosm studies

A literature review was conducted to identify meso- and microcosm study outcomes that could be compared to the data derived in the current study for further validation of the TRS predictions. Field-based, micro- and mesocosm data are produced using methods distinct from those employed for laboratory-based data, as they aim to reflect more environmentally realistic conditions (79). It was therefore difficult to directly compare estimates of toxicity derived from these studies to those of the TRS, as there were several key differences in experimental design. Many of the meso- and microcosm studies involved single dose or multiple dose regimes rather than continuous exposure. Continuous exposure, as it occurs in parts of the Great Barrier Reef catchment area (80, 82), may accelerate or increase the toxicity of imidacloprid to some taxa due to the lack of recovery time between pulses (71). In addition, although many studies differentiate between direct and indirect effects, it is generally difficult to isolate these patterns. A decrease in population density of a species following insecticide application may be considered a direct effect by some authors; however, it could also be the result of an indirect effect due to shifts in species interactions within the ecosystem. A high variability in reported toxicity values was also observed in the literature, in line with the findings of Wijngaarden, Brock, et al. (85) who conducted a review of threshold levels of insecticide effects in freshwater ecosystems. They attribute this variation to '*ecological properties of the test systems, the experimental set-up and frequency of observations used, organisms studied and taxonomic level of identification, and ecotoxicological profile of the insecticide'*.

That being said, meso-and microcosm studies still provide several valuable 'lines of evidence' for sanity-checking the results of the current study. This is because the TRS uses a chronic SSD with the aim of identifying the concentrations needed to protect long-term ecosystem structure and function from imidacloprid exposure. If long-term ecosystem-level effects are observed in meso- and microcosm studies using neonicotinoids, then this validates the use of the SSD approach for the TRS. Thus, meso- and microcosm studies can be used to determine whether the protective concentrations that are derived from the TRS are protective enough under experimentally realistic conditions. The protective concentrations derived from the TRS should therefore be similar to the experimental chronic IC10/EC10/EC10/NEC or NOEC data reported for individual species.

A number of studies were identified that could be used for broad comparisons under a 'multiple lines of evidence' approach to validate results of the current study, although the list below is not exhaustive. In the first semi-field study with imidacloprid, a single application in rice mesocosms caused substantial impacts on aquatic communities at concentrations above 1 µg/L, while populations of some arthropod species never recovered after 118 days of post-exposure even when water concentrations were below the detection limit of 0.02 µg/L (64). The lowest level of effect (LOEC) in another rice semi-field study was determined at approximately 1 µg/L (24). As discussed above, Hayasaka, Kobashi, et al. (23) found evidence of the cumulative toxicity of imidacloprid in rice mesocosms treated with a single application of this insecticide in two consecutive years. Zooplankton and arthropod community impacts were felt at concentrations of imidacloprid below 1 µg/L in their 4-month studies. Using mesocosms under Mediterranean conditions, Rico, Arenas-Sánchez, et al. (61) found that Cyclopoid copepods, the mayfly *Cloeon dipterum* and midges (Chironomini) showed the highest sensitivity to neonicotinoids, with calculated NOECs below 0.2 µg/L for individual species. Similar results were observed in sub-tropical freshwater microcosms in Bangladesh, where the zooplankton community NOEC was consistently 0.03 µg/L imidacloprid for all three treatments over a 28-day period (72). Using the

TRS method, a concentration of 0.03 µg/L for 28 days would result in approximately 12% of the ecosystem potentially affected. This higher estimate is likely due to the TRS including toxicity data for more sensitive taxa (e.g. mayflies), as well as crustacea. In a tropical mesocosm study by Merga and Van den Brink (46), the aquatic community structure in Ethiopia was significantly affected at ≥ 0.02 µg/L of imidacloprid, 112-day time-weighted average (TWA). Our TRS concentration of 0.02 µg/L produces a PAF of 34% following 100 days of continuous exposure, which is likely to produce both direct and indirect ecosystem effects. These observations therefore appear reasonable. Being below the effect concentration observed in the study, we can deem the TRS PC95 at 100 days to be protective of that particular ecosystem. Pestana, Alexander, et al. (55) investigated the effect of pulsed imidacloprid application on the structural and functional responses of benthic invertebrate assemblages in outdoor stream mesocosms. They found that even a low dose of 2 µg/L resulted in a 30% reduction in the abundance of Ephemeroptera, Plecoptera, and Trichoptera species. Beketov, Schäfer, et al. (10) found that a single thiacloprid pulse of 3.2 µg/L caused a significant alteration in a stream community structure that included 35 macroinvertebrate taxa. The effect persisted until the experiment was terminated and was more pronounced for long-lived species (at least 7 months to recover) than short-lived species (10 weeks to recover). In their mesocosm, the initial dose of thiacloprid was still detectable at a concentration of 0.05 µg/L after 9 days, which equates to a TWA concentration of 1.625 µg/L. A similar outcome was reported by Liess and Beketov (38), who found that short-lived aquatic species showed transient effects when the mesocosm was dosed with 3.3 µg/L of thiacloprid. Long-lived species were impacted at 0.1 µg/L and did not recover in the 12 months following contamination. Schmidt, Miller, et al. (68) reported top-down cascading effects linked to mixtures of imidacloprid and clothianidin in lotic mesocosms in a laboratory setting. These authors also found a synergistic effect of the binary mixture of these two neonicotinoids at low concentrations (0.5 µg/L + 0.5 µg/L) on the abundance of mayflies. Duchet, Hou, et al. (17) also found that mixtures of environmentally relevant neonicotinoid concentrations induced a top-down cascading effect on insect predators and zooplankton, which indirectly increased the phytoplankton community due to lack of grazing; however, no endpoints were derived for community effects. These studies illustrate that ecosystem impacts (in the form of altered community structure and function) are possible at concentrations that can reasonably be expected to occur in some parts of the Great Barrier Reef catchment area (40). This means that the chronic SSD approach that forms the basis of the TRS is justified. In addition, PC95 values were extracted from the TRS at exposure durations that aligned to the meso- and microcosm exposures. These were consistently more protective than the concentrations at which the ecosystem-level effects were observed.

References

(1) Ahlers, J.; Riedhammer, C.; Vogliano, M.; Ebert, R.-U.; Kühne, R.; Schüürmann, G. Acute to chronic ratios in aquatic toxicity-variation across trophic levels and relationship with chemical structure. *Environmental Toxicology and Chemistry* **2006**, *25* (11), 2937-2945. DOI: 10.1897/05-701R.1.

(2) Ahmed, M. A. I.; Othman, A. A.-E. Piperonyl Butoxide Enhances the Insecticidal Toxicity of Nanoformulation of Imidacloprid on Culex pipiens (Diptera: Culicidae) Mosquito. *Vector Borne Zoonotic Dis* **2020**, *20* (2), 134-142. DOI: 10.1089/vbz.2019.2474.

(3) Alexander, A. C.; Culp, J. M.; Liber, K.; Cessna, A. J. Effects of insecticide exposure on feeding inhibition in mayflies and oligochaetes. *Environmental Toxicology and Chemistry* **2007**, *26* (8), 1726-1732. DOI: 10.1897/07-015R.1.

(4) Anatra-Cordone, M.; Durkin, P. Imidacloprid - Human Health and Ecological Risk Assessment–Final Report (Prepared for: USDA, Forest Service). **2005**.

(5) ANZG. Australian and New Zealand Guidelines for Fresh and Marine Water Quality'. (Australian and New Zealand Governments and Australian State and Territory Governments, Canberra, Australia.). 2018.

(6) Ashauer, R.; Hintermeister, A.; Potthoff, E.; Escher, B. I. Acute toxicity of organic chemicals to Gammarus pulex correlates with sensitivity of Daphnia magna across most modes of action. *Aquat Toxicol* **2011**, *103* (1), 38-45. DOI: 10.1016/j.aquatox.2011.02.002.

(7) Bartlett, A. J.; Hedges, A. M.; Intini, K. D.; Brown, L. R.; Maisonneuve, F. J.; Robinson, S. A.; Gillis, P. L.; de Solla, S. R. Acute and chronic toxicity of neonicotinoid and butenolide insecticides to the freshwater amphipod, Hyalella azteca. *Ecotoxicol Environ Saf* **2019**, *175*, 215-223. DOI: 10.1016/j.ecoenv.2019.03.038.

(8) Batley, G.; Van Dam, R.; Warne M St J; Chapman, J.; Fox, D.; Hickey, C.; Stauber, J. Technical rationale for changes to the method for deriving Australian and New Zealand water quality guideline values for toxicants. *Australian Government Standing Council on Environment and Water, Canberra* **2014**.

(9) Beketov, M. A.; Liess, M. Potential of 11 Pesticides to Initiate Downstream Drift of Stream Macroinvertebrates. *Arch Environ Contam Toxicol* **2008**, *55* (2), 247-253. DOI: 10.1007/s00244-007-9104-3.

(10) Beketov, M. A.; Schäfer, R. B.; Marwitz, A.; Paschke, A.; Liess, M. Long-term stream invertebrate community alterations induced by the insecticide thiacloprid: Effect concentrations and recovery dynamics. *Sci Total Environ* **2008**, *405* (1), 96-108. DOI: 10.1016/j.scitotenv.2008.07.001.

(11) Brüggemann, M.; Hund-Rinke, K.; Böhmer, W.; Schaefers, C. Development of an Alternative Test System for Chronic Testing of Lotic Macroinvertebrate Species: A Case Study with the Insecticide Imidacloprid. *Environ Toxicol Chem* **2021**, *40* (8), 2229-2239. DOI: 10.1002/etc.5070.

(12) *Statistical software package to generate trigger values for local conditions within Australia*; CSIRO (http://www.csiro.au): 2016. [Online] Available from: https://research.csiro.au/software/burrlioz/. (accessed.

(13) Calabrese, E. J.; Baldwin, L. A. *Performing ecological risk assessments*; Lewis Publishers, 1993.

(14) Camp, A. A.; Buchwalter, D. B. Can't take the heat: Temperature-enhanced toxicity in the mayfly Isonychia bicolor exposed to the neonicotinoid insecticide imidacloprid. *Aquatic toxicology* **2016**, *178*, 49-57. DOI: 10.1016/j.aquatox.2016.07.011.

(15) Cavallaro, M. C.; Morrissey, C. A.; Headley, J. V.; Peru, K. M.; Liber, K. Comparative chronic toxicity of imidacloprid, clothianidin, and thiamethoxam to Chironomus dilutus and estimation of toxic equivalency factors. *Environ Toxicol Chem* **2017**, *36* (2), 372-382. DOI: 10.1002/etc.3536.

(16) *shinyssdtools: A web application for fitting Species Sensitivity Distributions (SSDs), version 1.0.6*; 2021. [Online] Available from: https://bcgov-env.shinyapps.io/ssdtools/ (accessed.

(17) Duchet, C.; Hou, F.; Sinclair, C. A.; Tian, Z.; Kraft, A.; Kolar, V.; Kolodziej, E. P.; McIntyre, J. K.; Stark, J. D. Neonicotinoid mixture alters trophic interactions in a freshwater aquatic invertebrate community. *Science of The Total Environment* **2023**, *897*, 165419. DOI: https://doi.org/10.1016/j.scitotenv.2023.165419.

(18) Fox, D.; van Dam, R.; Batley, G.; Fisher, R.; Thorley, J. Improved SSD software coming soon for the ANZG guidelines. *Australasian Bulletin of Ecotoxicology and Environmental chemistry letters* **2023**, *Volume 9, Pages 1-3*.

(19) Fox, D. R.; van Dam, R. A.; Fisher, R.; Batley, G. E.; Tillmanns, A. R.; Thorley, J.; Schwarz, C. J.; Spry, D. J.; McTavish, K. Recent Developments in Species Sensitivity Distribution Modeling. *Environmental Toxicology and Chemistry* **2021**, *40* (2), 293-308. DOI: https://doi.org/10.1002/etc.4925 (acccessed 2023/07/12).

(20) Freeman, J. B.; Dale, R. Assessing bimodality to detect the presence of a dual cognitive process. *Behav Res Methods* **2013**, *45* (1), 83-97. DOI: 10.3758/s13428-012-0225-x.

(21) Green, R. H. Estimation of Tolerance over an Indefinite Time Period. *Ecology (Durham)* **1965**, *46* (6), 887-887. DOI: 10.2307/1934028.

(22) Hano, T.; Ito, K.; Ohkubo, N.; Sakaji, H.; Watanabe, A.; Takashima, K.; Sato, T.; Sugaya, T.; Matsuki, K.; Onduka, T.; Ito, M.; Somiya, R.; Mochida, K. Occurrence of neonicotinoids and fipronil in estuaries and their potential risks to aquatic invertebrates. *Environmental Pollution* **2019**, *252*, 205-215. DOI: https://doi.org/10.1016/j.envpol.2019.05.067.

(23) Hayasaka, D.; Kobashi, K.; Hashimoto, K. Community responses of aquatic insects in paddy mesocosms to repeated exposures of the neonicotinoids imidacloprid and dinotefuran. *Ecotoxicology and Environmental Safety* **2019**, *175*, 272-281. DOI: https://doi.org/10.1016/j.ecoenv.2019.03.051.

(24) Hayasaka, D.; Korenaga, T.; Sánchez-Bayo, F.; Goka, K. Differences in ecological impacts of systemic insecticides with different physicochemical properties on biocenosis of experimental paddy fields. *Ecotoxicology* **2012**, *21* (1), 191-201. DOI: 10.1007/s10646-011-0778-y.

(25) Hayasaka, D.; Korenaga, T.; Suzuki, K.; Saito, F.; Sánchez-Bayo, F.; Goka, K. Cumulative ecological impacts of two successive annual treatments of imidacloprid and fipronil on aquatic communities of paddy mesocosms. *Ecotoxicol Environ Saf* **2012**, *80*, 355-362. DOI: 10.1016/j.ecoenv.2012.04.004.

(26) Heger, W.; Jung, S. J.; Martin, S.; Peter, H. Acute and prolonged toxicity to aquatic organisms of new and existing chemicals and pesticides. 1. Variability of the acute to prolonged ratio 2. Relation to logPow and Water Solubility. *Chemosphere (Oxford)* **1995**, *31* (2), 2707-2726. DOI: 10.1016/0045-6535(95)00127-T.

(27) Huang, A.; Mangold-Döring, A.; Focks, A.; Zhang, C.; Van den Brink, P. J. Comparing the acute and chronic toxicity of flupyradifurone and imidacloprid to non-target aquatic arthropod species. *Ecotoxicology and Environmental Safety* **2022**, *243*, 113977. DOI: https://doi.org/10.1016/j.ecoenv.2022.113977.

(28) Huang, A.; van den Brink, N. W.; Buijse, L.; Roessink, I.; van den Brink, P. J. The toxicity and toxicokinetics of imidacloprid and a bioactive metabolite to two aquatic arthropod species. *Aquat Toxicol* **2021**, *235*, 105837-105837. DOI: 10.1016/j.aquatox.2021.105837.

(29) Huryn, D. Temperature-dependent growth and life cycle of Deleatidium (Ephemeroptera: Leptophlebiidae) in two high-country streams in New Zealand. *Freshwater biology* **1996**, *36* (2), 351-361. DOI: 10.1046/j.1365-2427.1996.00098.x.

(30) Ieromina, O.; Peijnenburg, W. J. G. M.; de Snoo, G.; Müller, J.; Knepper, T. P.; Vijver, M. G. Impact of imidacloprid on Daphnia magna under different food quality regimes. *Environ Toxicol Chem* **2014**, *33* (3), 621-631. DOI: 10.1002/etc.2472.

(31) James, G.; Witten, D.; Hastie, T.; Tibshirani, R.; Tibshirani, R.; Hastie, T.; Witten, D. *An Introduction to Statistical Learning: with Applications in R*; Springer Nature, 2013. DOI: 10.1007/978-1-4614-7138-7.

(32) Kenaga, E. E. Predictability of chronic toxicity from acute toxicity of chemicals in fish and aquatic invertebrates. *Environmental Toxicology and Chemistry* **1982**, *1* (4), 347-358. DOI: 10.1002/etc.5620010410.

(33) Key, P.; Chung, K.; Siewicki, T.; Fulton, M. Toxicity of three pesticides individually and in mixture to larval grass shrimp (Palaemonetes pugio). *Ecotoxicol Environ Saf* **2007**, *68* (2), 272-277. DOI: 10.1016/j.ecoenv.2006.11.017.

(34) King, O. C.; Smith, R. A.; Mann, R. M.; Warne M St J. Proposed aquatic ecosystem protection guideline values for pesticides commonly used in the Great Barrier Reef catchment area: Part 1 (amended) – 2,4-D, Ametryn, Diuron, Glyphosate, Hexazinone, Imazapic, Imidacloprid, Isoxaflutole, Metolachlor, Metribuzin, Metsulfuron-methyl, Simazine and Tebuthiuron. **2018**, 296 pages.

(35) Knysh, K. M.; Courtenay, S. C.; Grove, C. M.; van den Heuvel, M. R. The Differential Effects of Salinity Level on Chlorpyrifos and Imidacloprid Toxicity to an Estuarine Amphipod. *Bull Environ Contam Toxicol* **2021**, *106* (5), 753-758. DOI: 10.1007/s00128-021-03157-z.

(36) Kumar, A.; Correll, R.; Grocke, S.; Bajet, C. Toxicity of selected pesticides to freshwater shrimp, Paratya australiensis (Decapoda: Atyidae): Use of time series acute toxicity data to predict chronic lethality. *Ecotoxicol Environ Saf* **2010a**, *73* (3), 360-369. DOI: 10.1016/j.ecoenv.2009.09.001.

(37) Li, H.; Zhang, Q.; Su, H.; You, J.; Wang, W.-X. High Tolerance and Delayed Responses of Daphnia magna to Neonicotinoid Insecticide Imidacloprid: Toxicokinetic and Toxicodynamic Modeling. *Environmental Science & Technology* **2021**, *55* (1), 458-467. DOI: 10.1021/acs.est.0c05664.

(38) Liess, M.; Beketov, M. Traits and stress: keys to identify community effects of low levels of toxicants in test systems. *Ecotoxicology* **2011**, *20* (6), 1328-1340. DOI: 10.1007/s10646-011-0689-y.

(39) Macaulay, S. J.; Buchwalter, D. B.; Matthaei, C. D. Water temperature interacts with the insecticide imidacloprid to alter acute lethal and sublethal toxicity to mayfly larvae. *New Zealand journal of marine and freshwater research* **2020**, *54* (1), 115-130. DOI: 10.1080/00288330.2019.1614961.

(40) Macaulay, S. J.; Hageman, K. J.; Alumbaugh, R. E.; Lyons, S. M.; Piggott, J. J.; Matthaei, C. D. Chronic Toxicities of Neonicotinoids to Nymphs of the Common New Zealand Mayfly Deleatidium spp. *Environ Toxicol Chem* **2019**, *38* (11), 2459-2471. DOI: 10.1002/etc.4556.
(41) Major, K. M.; Brander, S. M.; Eljarrat, E.; Eljarrat, E. Pyrethroid Insecticides. Vol. 92; Springer International Publishing AG, 2020; pp 109-148.
(42) Malev, O.; Klobučar, R. S.; Fabbretti, E.; Trebše, P. Comparative toxicity of imidacloprid and its transformation product 6-chloronicotinic acid to non-target aquatic organisms: Microalgae Desmodesmus subspicatus and amphipod Gammarus fossarum. *Pesticide biochemistry and physiology* **2012**, *104* (3), 178-186. DOI: 10.1016/j.pestbp.2012.07.008.
(43) Mangold-Döring, A.; Buddendorf, W. B.; van den Brink, P. J.; Baveco, J. M. How relevant are temperature corrections of toxicity parameters in population models for environmental risk assessment of chemicals? *Ecological modelling* **2024**, *498*, 110880. DOI: 10.1016/j.ecolmodel.2024.110880.
(44) May, M.; Drost, W.; Germer, S.; Juffernholz, T.; Hahn, S. Evaluation of acute-to-chronic ratios of fish and Daphnia to predict acceptable no-effect levels. *Environ Sci Eur* **2016**, *28* (1), 16-16. DOI: 10.1186/s12302-016-0084-7.
(45) Mayer, F. L.; Krause, G. F.; Ellersieck, M. R.; Lee, G.; Buckler, D. R. Predicting chronic lethality of chemicals to fishes from acute toxicity test data: Concepts and linear regression analysis. *Environmental Toxicology and Chemistry* **1994**, *13* (4), 671-678. DOI: 10.1002/etc.5620130418.
(46) Merga, L. B.; Van den Brink, P. J. Ecological effects of imidacloprid on a tropical freshwater ecosystem and subsequent recovery dynamics. *The Science of the total environment* **2021**, *784*, 147167-147167. DOI: 10.1016/j.scitotenv.2021.147167.
(47) Minton, K. A. The role of temperature variation in neonicotinoid toxicity and species sensitivity distributions to temperature. Texas Tech University Library: Electronic Theses and Dissertations, 2015. http://hdl.handle.net/2346/63658. Date accessed: 22/11/2021.
(48) Morrissey, C. A.; Mineau, P.; Devries, J. H.; Sanchez-Bayo, F.; Liess, M.; Cavallaro, M. C.; Liber, K. Neonicotinoid contamination of global surface waters and associated risk to aquatic invertebrates: A review. *Environment International* **2015**, *74*, 291-303. DOI: https://doi.org/10.1016/j.envint.2014.10.024.
(49) Negri, A.; Templeman, S.; Flores, F.; Van Dam, J.; Thomas, M.; McKenzie, M.; Stapp, L.; Kaserzon, S.; Mann, R.; Smith, R. Ecotoxicology of pesticides on the Great Barrier Reef for guideline development and risk assessments. *Final report to the National Environmental Science Program. Reef and Rainforest Research Centre Limited, Cairns (P 120). Available from* **2020**.
(50) Njattuvetty Chandran, N.; Fojtova, D.; Blahova, L.; Rozmankova, E.; Blaha, L. Acute and (sub)chronic toxicity of the neonicotinoid imidacloprid on Chironomus riparius. *Chemosphere* **2018**, *209*, 568-577. DOI: 10.1016/j.chemosphere.2018.06.102.
(51) Ockleford, C.; Aiaanse, P.; Berny, P.; Brock, T.; Duquesne, S.; Grilli, S.; Hernandez-Jerez, A. F.; Bennekou, S. H.; Klein, M.; Kuhl, T.; Laskowski, R.; Machera, K.; Pelkonen, O.; Pieper, S.; Smith, R. H.; Stemmer, M.; Sundh, I.; Tiktak, A.; Topping, C. J.; Wolterink, G.; Cedergreen, N.; Charles, S.; Focks, A.; Reed, M.; Arena, M.; Ippolito, A.; Byers, H.; Teodorovic, I. Scientific Opinion on the state of the art of Toxicokinetic/Toxicodynamic (TKTD) effect models for regulatory risk assessment of pesticides for aquatic organisms. *EFSA journal* **2018**, *16* (8), e05377-n/a. DOI: 10.2903/j.efsa.2018.5377.
(52) OECD, Organisation for Economic, Co-operation; Development, Q. f. E. C.-o.; Development. *Current Approaches in the Statistical Analysis of Ecotoxicity Data A guidance to application (annexes to this publication exist as a separate document)*; OECD Publishing, 2006.
(53) Osterberg, J. S. Ecotoxicology of natural and anthropogenic extreme environments. ProQuest Dissertations Publishing, 2010.
(54) Overmyer, J. P.; Mason, B. N.; Armbrust, K. L. Acute toxicity of imidacloprid and fipronil to a nontarget aquatic insect, Simulium vittatum Zetterstedt cytospecies IS-7. *Bull Environ Contam Toxicol* **2005**, *74* (5), 872-879. DOI: 10.1007/s00128-005-0662-7.
(55) Pestana, J. L. T.; Alexander, A. C.; Culp, J. M.; Baird, D. J.; Cessna, A. J.; Soares, A. M. V. M. Structural and functional responses of benthic invertebrates to imidacloprid in outdoor stream mesocosms. *Environ Pollut* **2009**, *157* (8), 2328-2334. DOI: 10.1016/j.envpol.2009.03.027.
(56) Pietrzak, B.; Bednarska, A.; Markowska, M.; Rojek, M.; Szymanska, E.; Slusarczyk, M. Behavioural and physiological mechanisms behind extreme longevity in Daphnia. *Hydrobiologia* **2013**, *715* (1), 125-134. DOI: 10.1007/s10750-012-1420-6.

(57) Raby, M.; Nowierski, M.; Perlov, D.; Zhao, X.; Hao, C.; Poirier, D. G.; Sibley, P. K. Acute toxicity of 6 neonicotinoid insecticides to freshwater invertebrates. *Environ Toxicol Chem* **2018**, *37* (5), 1430-1445. DOI: 10.1002/etc.4088.
(58) Raby, M.; Zhao, X.; Hao, C.; Poirier, D. G.; Sibley, P. K. Chronic toxicity of 6 neonicotinoid insecticides to Chironomus dilutus and Neocloeon triangulifer: Chronic toxicity of neonicotinoids to aquatic invertebrates. *Environmental toxicology and chemistry* **2018**, *37* (10), 2727-2739. DOI: 10.1002/etc.4234.
(59) Raimondo, S.; Montague, B. J.; Barron, M. G. Determinants of variability in acute to chronic toxicity ratios for aquatic invertebrates and fish. *Environmental Toxicology and Chemistry* **2007**, *26* (9), 2019-2023. DOI: 10.1897/07-069R.1.
(60) Ranatunga, M.; Kellar, C.; Pettigrove, V. Toxicological impacts of synthetic pyrethroids on non-target aquatic organisms: A review. *Environmental advances* **2023**, *12*, 100388. DOI: 10.1016/j.envadv.2023.100388.
(61) Rico, A.; Arenas-Sánchez, A.; Pasqualini, J.; García-Astillero, A.; Cherta, L.; Nozal, L.; Vighi, M. Effects of imidacloprid and a neonicotinoid mixture on aquatic invertebrate communities under Mediterranean conditions. *Aquat Toxicol* **2018**, *204*, 130-143. DOI: 10.1016/j.aquatox.2018.09.004.
(62) Roessink, I.; Merga, L. B.; Zweers, H. J.; Van den Brink, P. J. The neonicotinoid imidacloprid shows high chronic toxicity to mayfly nymphs. *Environ. Toxicol. Chem* **2013**, *32* (5), 1096-1100. DOI: 10.1002/etc.2201.
(63) Sánchez-Bayo, F. From simple toxicological models to prediction of toxic effects in time. *Ecotoxicology* **2009**, *18* (3), 343-354. DOI: 10.1007/s10646-008-0290-1.
(64) Sánchez-Bayo, F.; Goka, K. Ecological effects of the insecticide imidacloprid and a pollutant from antidandruff shampoo in experimental rice fields. *Environmental Toxicology and Chemistry* **2006**, *25* (6), 1677-1687. DOI: 10.1897/05-404R.1.
(65) Sánchez-Bayo, F.; Goka, K. Influence of light in acute toxicity bioassays of imidacloprid and zinc pyrithione to zooplankton crustaceans. *Aquat Toxicol* **2006**, *78* (3), 262-271. DOI: 10.1016/j.aquatox.2006.03.009.
(66) Sánchez-Bayo, F.; Goka, K.; Hayasaka, D. Contamination of the aquatic environment with neonicotinoids and its implication for ecosystems. *Frontiers in environmental science* **2016**, *4*. DOI: 10.3389/fenvs.2016.00071.
(67) Sánchez-Bayo, F.; Tennekes, H. A. Time-cumulative toxicity of neonicotinoids: Experimental evidence and implications for environmental risk assessments. *Int J Environ Res Public Health* **2020**, *17* (5), 1629. DOI: 10.3390/ijerph17051629.
(68) Schmidt, T. S.; Miller, J. L.; Mahler, B. J.; Van Metre, P. C.; Nowell, L. H.; Sandstrom, M. W.; Carlisle, D. M.; Moran, P. W.; Bradley, P. M. Ecological consequences of neonicotinoid mixtures in streams. *Sci Adv* **2022**, *8* (15), eabj8182-eabj8182. DOI: 10.1126/sciadv.abj8182.
(69) Smit, C. E.; Posthuma-Doodeman, C. J. A. M.; van Vlaardingen, P. L. A.; de Jong, F. M. W. Ecotoxicity of Imidacloprid to Aquatic Organisms: Derivation of Water Quality Standards for Peak and Long-Term Exposure. *Human and ecological risk assessment* **2015**, *21* (6), 1608-1630. DOI: 10.1080/10807039.2014.964071.
(70) Sprague, J. B. Measurement of pollutant toxicity to fish I. Bioassay methods for acute toxicity. *Water Research* **1969**, *3* (11), 793-821. DOI: 10.1016/0043-1354(69)90050-5.
(71) Stoughton, S. J.; Liber, K.; Culp, J.; Cessna, A. Acute and Chronic Toxicity of Imidacloprid to the Aquatic Invertebrates Chironomus tentans and Hyalella azteca under Constant- and Pulse-Exposure Conditions. *Arch Environ Contam Toxicol* **2008**, *54* (4), 662-673. DOI: 10.1007/s00244-007-9073-6.
(72) Sumon, K. A.; Ritika, A. K.; Peeters, E. T. H. M.; Rashid, H.; Bosma, R. H.; Rahman, M. S.; Fatema, M. K.; Van den Brink, P. J. Effects of imidacloprid on the ecology of sub-tropical freshwater microcosms. *Environmental Pollution* **2018**, *236*, 432-441. DOI: https://doi.org/10.1016/j.envpol.2018.01.102.
(73) *ssdtools: An R package to fit Species Sensitivity Distributions, version 1.0.6*; Journal of Open Source Software, 2018. [Online] Available from: https://cran.r-project.org/web/packages/ssdtools/index.html, user manual: https://cran.r-hub.io/web/packages/ssdtools/vignettes/ssdtools-manual.html (accessed 01/07/2023).
(74) Thursby, G.; Sappington, K.; Etterson, M. Coupling toxicokinetic–toxicodynamic and population models for assessing aquatic ecological risks to time-varying pesticide exposures. *Environ Toxicol Chem* **2018**, *37* (10), 2633-2644. DOI: 10.1002/etc.4224.
(75) Tomizawa, M.; Lee, D. L.; Casida, J. E. Neonicotinoid Insecticides: Molecular Features Conferring Selectivity for Insect versus Mammalian Nicotinic Receptors. *J. Agric. Food Chem* **2000**, *48* (12), 6016-6024. DOI: 10.1021/jf000873c.
(76) ECOTOX User Guide: ECOTOXicology Database System. Version 4.0. . http://cfpub.epa.gov/ecotox/ (accessed 22/11/2021).
(77) Office of Pesticide Programs (OPP) Pesticide Ecotoxicity Database. Ecological Fate and Effects Division, Office of Pesticide Programs. https://cfpub.epa.gov/ecotox/ (accessed 22/11/2021).

(78) Van den Brink, P. J.; Van Smeden, J. M.; Bekele, R. S.; Dierick, W.; De Gelder, D. M.; Noteboom, M.; Roessink, I. Acute and chronic toxicity of neonicotinoids to nymphs of a mayfly species and some notes on seasonal differences. *Environmental Toxicology and Chemistry* **2016**, *35* (1), 128-133, https://doi.org/10.1002/etc.3152. DOI: https://doi.org/10.1002/etc.3152 (acccessed 2023/06/07).
(79) Warne M St J; Batley, G. E.; van Dam, R. A., Chapman, J.C., ; Fox, D. R.; Hickey, C. W.; Stauber, J. L. Revised method for deriving Australian and New Zealand water quality guideline values for toxicants – update of 2015 version. Prepared for the revision of the Australian and New Zealand Guidelines for Fresh and Marine Water Quality. **2018**, 48.
(80) Warne M St J; Turner, R. D. R.; Davis, A. M.; Smith, R.; Huang, A. Temporal variation of imidacloprid concentration and risk in waterways discharging to the Great Barrier Reef and potential causes. *Sci Total Environ* **2022**, *823*, 153556-153556. DOI: 10.1016/j.scitotenv.2022.153556.
(81) Warne M St J; Westbury, A.-M.; Sunderam, R. A compilation of toxicity data for chemicals to Australasian aquatic species. Part 1: Pesticides. *Australasian Journal of Ecotoxicology* **1998**, (4), 93-144.
(82) Catchment Loads Monitoring Program Pesticide Reporting Portal. Available: https://storymaps.arcgis.com/collections/9a61cdb7ff1143bd9eec98eccbc3b50e?item=2 (accessed 20/02/2025).
(83) Water Quality & Investigations. Great Barrier Reef Catchment Loads Monitoring Program: Program Design (methods) document,. Science, Q. D. o. E. a., Ed.; 2023.
(84) Wen, Y. H. Life history and production of Hyalella azteca (Crustacea: Amphipoda) in a hypereutrophic prairie pond in southern Alberta. *Canadian Journal of Zoology* **1992**, *70* (7), 1417-1424.
(85) Wijngaarden, R. P. A. V.; Brock, T. C. M.; Brink, P. J. V. D. Threshold Levels for Effects of Insecticides in Freshwater Ecosystems: A Review. *Ecotoxicology* **2005**, *14* (3), 355-380. DOI: 10.1007/s10646-004-6371-x.
(86) Yates, L. A.; Aandahl, Z.; Richards, S. A.; Brook, B. W. Cross validation for model selection: A review with examples from ecology. *Ecological monographs* **2023**, *93* (1), n/a. DOI: 10.1002/ecm.1557.
(87) Yokoyama, A.; Ohtsu, K.; Iwafune, T.; Nagai, T.; Ishihara, S.; Kobara, Y.; Horio, T.; Endo, S. A useful new insecticide bioassay using first-instar larvae of a net-spinning caddisfly, Cheumatopsyche brevilineata (Trichoptera: Hydropsychidae). *J. Pestic. Sci.* **2009**, *34* (1), 13-20. DOI: 10.1584/jpestics.G08-26.
